# Subtle alteration in transcriptional memory governs the lineage-level cell cycle duration heterogeneities of mammalian cells

**DOI:** 10.1016/j.isci.2025.112981

**Published:** 2025-06-21

**Authors:** Kajal Charan, Sandip Kar

**Affiliations:** 1 Department of Chemistry, IIT Bombay, Powai, Mumbai 400076, India

**Keywords:** Organizational aspects of cell biology, In silico biology, Biological constraints, Transcriptomics

## Abstract

Cell cycle duration heterogeneities within a malignant tumor significantly reduce the therapeutic efficacy of cancer treatment. However, identifying factors governing such heterogeneities remains challenging. Herein, we perform a computational modeling study to demonstrate that correlated fluctuations of the inherited transcription rates during cell cycle progression and their random resetting in mitosis are sufficient to account for the experimentally observed cell cycle duration correlation patterns for cell lineage pairs. The model elucidates that the variations in the transcriptional inheritance pattern dictate the extent of the cousin-mother inequality phenomenon in a cell-type-dependent manner. Intriguingly, the model predicts that a reduction in cousin-mother inequality for a fixed mean cell cycle duration can lead to a lowering of the cell cycle duration variabilities at the population level which may have a therapeutic implication. Overall, our study elucidates that correlated transcriptional fluctuations can be the sole governing factor in orchestrating the cell cycle duration heterogeneities.

## Highlights

• Correlated transcription rate fluctuations govern the cell lineage variabilities
• High transcription rate fluctuations lead to higher daughter-daughter correlations
• ⁠Transcriptional inheritance pattern alters the extent of cousin-mother inequality
• ⁠⁠Population-level variabilities can be lowered by reducing cousin-mother inequality

## Graphical abstract

**Figure:**
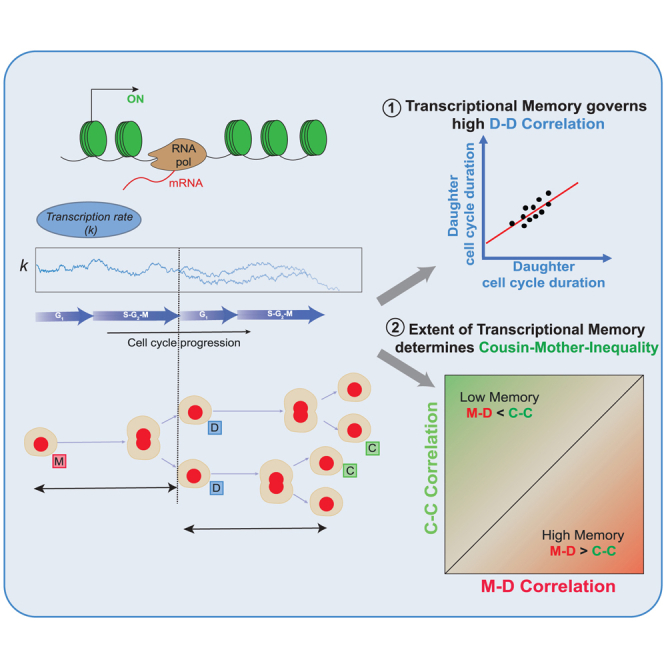

## Teaser

Organizational aspects of cell biology; In silico biology; Biological constraints; Transcriptional memory

## Introduction

Cells within a malignant tumor demonstrate a high degree of heterogeneity in their proliferation pattern.^1^^,^^2^^,^^3^^,^^4^ This makes the majority of chemotherapies ineffective in eradicating cancerous cells.^5^^,^^6^^,^^7^^,^^8^ Thus, understanding the origin of such heterogeneities is imperative to uncover the governing factors influencing the proliferative nature of mammalian cells. Over the last few decades, researchers have engaged themselves in quantifying the temporal dynamics of mammalian cell cycle regulation by tracking single cells for several cell lineages with the help of novel cell cycle reporter systems.^9^^,^^10^^,^^11^^,^^12^^,^^13^^,^^14^ In these studies, the cell cycle period and phase durations were calculated for different cell lineages to quantify the correlation among these timings for mother-daughter (M-D), sister-sister (D-D), and cousin-cousin (C-C) cell pairs.^10^^,^^11^^,^^12^^,^^14^ It has been observed that even under culture conditions, mammalian cells display significant variations in their cell cycle period and phase durations.^1^^,^^15^^,^^16^ These studies^10^^,^^11^^,^^12^^,^^13^^,^^14^^,^^17^ reveal that the sister pairs (D-D) correlate highly in their cell cycle duration, while the mother-daughter (M-D) and cousin-cousin (C-C) pairs exhibit relatively poorer correlations in comparison to the sister pairs. However, the origin of such kind of lineage-level correlation patterns in cell cycle duration remains unclear. Sandler et al.^10^ first proposed that these lineage-level correlations can be caused by background circadian rhythms present in mammalian cells and other subsequent studies support this concept.^17^^,^^18^ Kuchen et al.^14^ argued that it is manifested due to specific cell size and cell cycle speed inheritance. Hughes et al.^18^ suggested that it occurs due to the inheritance of a few cell cycle factors, while Govindaraj et al.^11^ proposed that these correlations can even be observed due to the inheritance of transcriptional fluctuations across generations. What do daughter cells inherit and how does such inheritance govern these lineage-level cell cycle duration heterogeneities remains elusive.

Intriguingly, in some of these experimental studies involving various bacterial^19^^,^^20^ as well as cancerous cell types,^10^^,^^11^^,^^17^ it was shown the C-C cell cycle duration correlation is higher compared to the M-D pairs. This phenomenon is known as cousin-mother inequality (**CMI**). ^10^^,^^11^^,^^17^ However, in other studies involving mouse embryonic fibroblasts^12^ and other cancerous cell lines,^14^ it was reported that the M-D correlation is either higher or comparable to the C-C correlations. ^12^^,^^14^^,^^18^ Thus, the occurrence of **CMI** is highly cell-type–dependent,^10^^,^^11^^,^^12^^,^^14^^,^^17^ and how such kind of dependency arises remains poorly understood. Moreover, Govindaraj et al.^11^ suggested in their study that the lineage-level correlations and population-level heterogeneities in cell cycle period and phase duration are intricately related to each other. These observations lead to many interesting questions. For example: (1) Why do we observe such kind of cell cycle duration heterogeneities? (2) Why are the sister pairs highly correlated? (3) How can C-C correlations exist when M-D pairs are poorly correlated in the first place? (4) Why does **CMI** not consistently hold for all cell types? (5) How is the presence or absence of **CMI** related to population-level heterogeneity, especially in the context of overall cell cycle duration for mammalian cells? Answering these questions will unravel why such kind of lineage-level correlations are conserved across different mammalian cell types and what causes such subtle variations in these correlation patterns. However, one can ask why we bother about these lineage-level correlations and what is the relevance of knowing them accurately for a specific cell type. Herein, we hypothesize that identifying the lineage-level correlation pattern for a specific cell type with a certain cell cycle period under ambient conditions will provide crucial initial information about how to perturb the respective cell cycle dynamics of a specific cell type (for example, a cancerous cell type) to reduce the population-level heterogeneities of the same by eventually making these cells therapeutically more sensitive. In this regard, can we get any insight from theoretical/computational modeling studies?

In the literature, various modeling approaches have been employed to explain the origin of such lineage-level correlation patterns for specific cell types to explain specific experimental findings, such as high degree of D-D correlations,^10^^,^^11^^,^^12^^,^^13^^,^^14^ presence^10^^,^^11^^,^^17^ and absence^12^^,^^14^ of CMI in a cell-type dependent manner, etc. However, the origin of such kind of duration heterogeneities at the lineage level remains vaguely understood. Indeed, each of these studies has put forward different reasonings to elucidate a universal phenomenon without addressing several other associated intricate features of the cell cycle duration heterogeneities. In a recent study, Hughes et al.^18^ explained the correlation pattern as a universal feature across various cell types. They suggested that some heritable cell cycle regulatory factors play the governing role in generating diverse correlation patterns in lineage pairs. How exactly such factors are inherited and how this kind of inheritance is modulated during the cell cycle to impact the correlation patterns remains unclear.

In this article, we analyze a simple cell cycle model that elucidates all the correlation-related aspects of the cell cycle duration heterogeneities at the lineage level in a comprehensive way. The model hypothesizes that the daughter cells within a lineage inherit the transcription rate of various cell cycle regulatory genes from their mother,^21^^,^^22^^,^^23^^,^^24^ and that these transcription rates then fluctuate in a correlated manner during cell cycle progression. Our model demonstrates that the variations of noise strengths and correlation times of such kind of correlated transcriptional fluctuations can explain the complex lineage-level cell cycle duration correlation patterns as observed experimentally for various mammalian cell types. The model further delineates how the cell cycle period and the individual phase durations affect the cell cycle duration heterogeneities in the presence of correlated transcriptional fluctuations.

### Model

**Figure 1:**
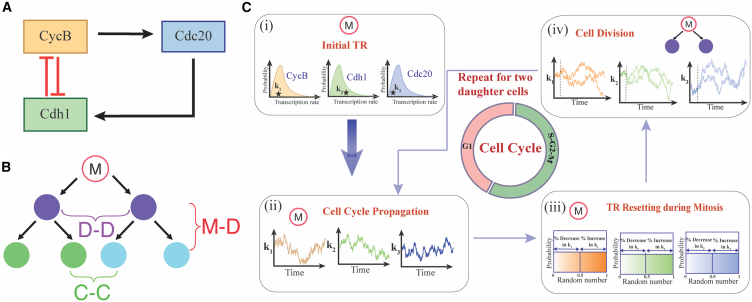
The proposed generic model and the simulation protocol to capture the lineage level cell cycle duration variabilities (A) The proposed cell cycle regulatory network.^25^ (B) A representative cell lineage tree. (C) The schematic numerical protocol for simulating cell cycle lineages by introducing transcriptional fluctuations in cell lineages and during the cell cycle progression. Step-(i) - Drawing the transcription rates (TR) $k_{1}$, $k_{2}$, and $k_{3}$ for each transcript (CycB, Cdh1, and Cdc20) from respective log-normal distributions of transcription rates; Step-(ii) – Simulating cell cycle dynamics assuming correlated transcription rate fluctuations for each transcript: Step-(iii) – Transcription rate resetting during the mitosis event for each transcripts: Step-(iv) – Implementing cell division event (indicated by the dotted vertical lines) and continuing simulating both the daughter cells by assuming inheritance of transcription rates from respective mother cell.

Our model is an improved version of a generic mammalian cell cycle model developed by Tyson and Novak.^25^ This model includes three regulatory proteins, CycB, Cdh1, and Cdc20 (Figure 1A), whose interactions control the entry into mitosis and subsequent exit from the cell cycle. Specifically, CycB and Cdh1 have an antagonistic relationship that maintains a low CycB level during the early G_1_ phase as the Cdh1 sustains a high concentration level. In the current model, we have assumed that an increase in the externally added growth factor (GF) activates CycB which can phosphorylate Cdh1 to make it inactive. Cells will then eventually transit from G_1_-phase to S-phase.^26^^,^^27^ However, a higher level of CycB activates the transcription of Cdc20 which dephosphorylates Cdh1 and facilitates the cell cycle exit (Detailed model in Figure S1A). Here, we have made sure that the period of the cell cycle does not vary much with change in the GF and produces high amplitude oscillations via an SNIC bifurcation (Figure S1B) which is generally observed in the earlier mammalian cell cycle models.^25^^,^^28^^,^^29^ Here, we endeavor to computationally follow several lineages of cells (Figure 1B) for a specified period (for instance, 72-h) as per live-cell imaging experiments.^11^^,^^14^^,^^17^ We have implemented the cell division event when CycB reaches a certain threshold level during late mitosis following previous literature^11^^,^^25^ (Figure S1C). Moreover, we have assumed that all the cellular components in the mother cell get equally distributed between the two daughter cells during the division event.

Importantly, we have included the dynamics of mRNAs (Tables S1–S3) for each regulatory protein in the model proposed by Tyson et al. to introduce the concept of correlated transcriptional inheritance across cellular generations.^21^^,^^22^^,^^30^^,^^31^^,^^32^^,^^33^ Here, it is important to note that there is evidences in the literature which suggest that across generations (in different cell types), daughter cells indeed inherit various aspects from their respective mothers. For example: (1) Sigal et al. have shown that in mammalian cells these correlation timescales in protein production can be even longer than one cell cycle duration and vary in a gene-dependent manner.^21^ The proteins considered by Sigal et al.^21^ (Table S4) control various processes such as transcriptional regulation, chromatin remodeling, cell proliferation and differentiation, alternative splicing, recruiting RNA polymerase to promoters, deubiquitination, regulating histones, nuclear membrane structure, apoptosis, etc. (2) Phillips et al.^22^ have observed that despite significant gene-level fluctuations, the mean transcriptional activity of various genes is restored in daughter cells from mothers within lineages in mouse embryonic stem cells.^22^ All these genes control critical processes of transcription and translation of all the genes present within the cell. This implies that the transcriptions of other genes involved in various regulatory processes (such as cell cycle, apoptosis, differentiation, etc.) of any cell will be affected in a correlated manner. (3) Rosenfeld et al.^30^ have demonstrated that slow fluctuations in protein production rates have a correlation timescale equivalent to the duration of a cell cycle in *Escherichia coli*.^30^ In a similar spirit, the transcriptional inheritance across generations in the mammalian cells may lead to correlated protein production rates that will have a correlation timescale equivalent to or greater than cell cycle period. Thus, we took forward this idea of correlated transcriptional regulation in our modeling work to understand the origin of lineage correlation in daughter cells and the **CMI** phenomenon. In our model (Tables S1–S3) we have assumed that daughter cells inherit the transcription rates of all genes from their mother. However, these transcription rates have certain correlated fluctuations during the cell cycle progression with non-zero correlation timescales. Although we start with the premise that the correlation times can be of the order cell cycle duration or more than that, in reality, we performed most of our simulation studies by keeping the transcription rate correlation times at max of the duration of cell cycle time or much lesser than cell cycle period, which seems to be a reasonable assumption, to begin with.

The time series of this correlated noise is generated using the Ornstein-Uhlenbeck process as described in Equation 1.^33^^,^^34^^,^^35^

(Equation 1) $$\frac{d\varepsilon_{ou}}{dt}=-\frac{1}{\tau_{au}}\varepsilon_{ou}+\frac{\varepsilon_{gn}}{\tau_{au}}$$

where $\varepsilon_{gn}$ is the delta correlated white noise with zero mean, i.e.,

$$\left\langle \varepsilon_{gn}\left(t\right)\right\rangle =0\;\&\;\left\langle \varepsilon_{gn}\left(t\right)\varepsilon_{gn}\left(t^{\prime }\right)\right\rangle =2D^{\prime }\delta \left(t-t^{\prime }\right)$$

Here, $\varepsilon_{ou}$ is the exponentially correlated noise that has a positive autocorrelation time $\tau_{au}$ and follows the relations $\left\langle \varepsilon_{ou}\left(t\right)\right\rangle =0$ & $\left\langle \varepsilon_{ou}\left(t\right)\varepsilon_{ou}\left(t^{\prime }\right)\right\rangle =\frac{D^{\prime }}{\tau_{au}}\exp\left(-\frac{t{-t}^{\prime }}{\tau_{au}}\;\right)$ . In our simulation, we generated the Gaussian white noise by picking random numbers from the Gaussian distribution which have noise strength $D=4D^{\prime }\Delta t$ (Δt is the time step of simulation).^34^

The overall simulation strategy can be divided into four steps. To initiate the lineage-level simulations at t=0, in Step-(i), the transcription rates of the CycB, Cdh1, and Cdc20 mRNAs are drawn from the respective lognormal distributions (with deterministic mean transcription rate and 5% coefficient of variation (CV)^36^^,^^37^) for a representative mother cell for a specific lineage (Figure 1C(i). In Step-(ii) of the simulation (Figure 1C(ii)), these transcription rates are propagated with colored noise having non-zero correlation timescales during cell cycle progression.^33^^,^^34^ This continues until we reach mitosis, where we have introduced the concept of transcription rate resetting for all the transcripts (Figure 1C(iii)) regulating the cell cycle in Step-(iii). Transcriptional resetting is implemented in our simulation by picking up random numbers from uniformly distributed random numbers between 0 and 1 for each transcript during mitosis. The respective transcription rate for each transcript is then either increased (∼10%) or decreased (∼10%) proportionately by finding whether the randomly drawn number is greater than or less than 0.5, respectively. The stochastic simulations are then continued with the newly obtained transcription rates. This transcriptional resetting in our simulations accounts for the transcription rate variations that happen during the mitotic phase, resulting from chromatids segregation into the two daughter cells.^23^^,^^38^^,^^39^ In Step-(iv), we have implemented the cell division event (Figure 1C(iv)) once the CycB protein level crosses the certain lower threshold level set for the cell division event.^11^^,^^25^ Upon cell division, the two daughter cells inherit all transcription rates from their mother.^21^ We repeat the same simulation protocol (Step-(ii) to Step-(iv)) for both the daughter cells until we reach the simulation end time (72 h).

## Results and discussions

### Alteration in transcriptional memory reconciles diverse cell cycle duration correlation patterns in lineage pairs

**Figure 2:**
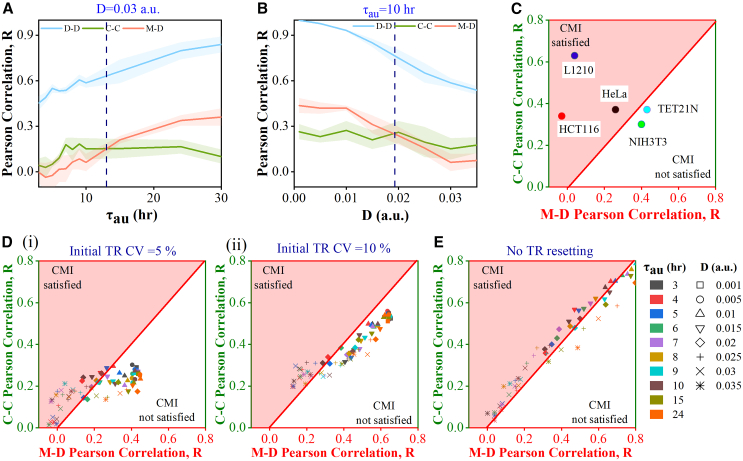
Different lineage correlation patterns emerge due to variabilities in transcriptional memory (A–E) Correlation in D-D, M-D, and C-C with change in (A) $\tau_{au}$ = 3-h to 30-h (fixed D = 0.03) and (B) change in D = 0 to $D_{\max}$ = 0.035 (fixed $\tau_{au}$ = 10-h). The dotted vertical line corresponds to the $\tau_{critical}$ (13-h) and $D_{critical}\;\left(0.0192\right)$, respectively. Plot of M-D cell cycle duration correlation against C-C correlation; (C) from experimental literature for different cell lines.,^10^^,^^11^^,^^12^^,^^14^^,^^17^ (D) from our simulations for $\tau_{au}$ ranging from 3-h to 30-h at different D for initial transcription rate (TR) distributions with CV (i) 5% and (ii) 10%, and (E) from our simulations without TR resetting at mitosis. The shaded area shows the region where **CMI** is satisfied. All simulations are performed for 3 replicates (under each combination of D and $\tau_{au}$) each for 300 cells. The error bar cloud in Figures 2A and 2B represents the S.E.M. (standard error of the mean) of 3 replicates.

Following the proposed simulation scheme (Figure 1C), we have been able to capture the diversity in the cell cycle duration heterogeneities across cell lineages (Figure 2). First, we demonstrate how transcriptional memory can generate correlation among lineage pairs by assuming the cell cycle duration to be 24-h (with 10-h of G_1_ and 14-h of S-G_2_-M durations). Our simulation at a fixed noise strength (D=0.03) with varying autocorrelation time ($\tau_{au}$) indicates that as $\tau_{au}$ increases, there is a rise in correlation among D-D and M-D lineage pairs but for the C-C pair, the level of the correlation saturates after an initial rise (Figure 2A). These observations can be attributed to the increasing extent of transcriptional memory. Interestingly, an intriguing pattern emerges as $\tau_{au}$ increases. At lower values of $\tau_{au}$, the M-D correlation is less than the C-C correlation, however, beyond a threshold value of $\tau_{au}$, the M-D correlation supersedes the C-C correlation (Figure 2A). This suggests that enhancement in transcriptional memory can diminish the initially observed **CMI** by having a greater impact on the correlation between M-D pairs than the correlation between C-C pairs. Experimentally, it has been shown that^21^ the autocorrelation time and the extent of variability in transcription rates vary notably across various genes. Thus, we simulate a similar simulation by varying the noise strength (D) while keeping the $\tau_{au}$ fixed at 10 h. The simulations reveal that with increasing D, all the lineage pair correlations decrease, and beyond a specific D value, there is an emergence of **CMI** (Figure 2B). It is important to emphasize that we have performed these simulations assuming a 24-h cell cycle period (with 10-hour G_1_ and 14-hour S-G_2_-M durations).

Experimentally, the presence and absence of **CMI** have been observed in a cell-type-dependent manner^10^^,^^11^^,^^12^^,^^14^^,^^17^ and we have summarized those experimental observations in Figure 2C. Will our model simulations be able to reproduce these variations in **CMI** by simply considering the varied extent of transcription rate variability? To answer this question, we have performed simulation studies to comprehensively understand the collective influence of $\tau_{au}$ and D on the variabilities in lineage pair correlations across a spectrum of $\tau_{au}$ and D values (Figure 2Di). From Figure 2Di, we can observe that some of the points lie above the red line and in the red zone which indicates that for these sets of $\tau_{au}$ and D values, we will observe **CMI** where C-C correlations are higher than the M-D correlation. However, there are other combinations of $\tau_{au}$ and D values for which we will observe M-D correlations to be higher than the C-C correlation (Figure 2Di). Thus, our simulations qualitatively reconcile the experimentally observed correlation pattern among M-D and C-C lineage pairs for different cell types (Figure 2C).^10^^,^^11^^,^^12^^,^^13^^,^^18^ Figure 2Di further delineates that for certain combinations of $\tau_{au}$ and D, we will observe **CMI** as seen in experimental studies.^10^^,^^11^^,^^18^ We must mention that we have numerically simulated nearly 300 cells as experimentally we can normally observe these many cells.^10^^,^^11^ However, we have confirmed that increasing the number of cells in our simulations does not change the qualitative nature of our results (Figure S2). Further, it has been observed that the CV for the initial log-normal distributions for various transcription rates (from which the initial set of transcription rates are randomly selected for the starting mother cells in each lineage) plays a substantial role in determining the correlations of cell cycle duration in D-D, M-D and C-C pairs.^22^

Next, we investigate how the lineage-level correlations in cell cycle durations get affected if the width (CV) of the log-normal distributions (from where we are picking the transcription rates for various cell cycle regulators) is increased by maintaining the same deterministic transcription rates for all the transcripts, respectively. At the population level, this increase leads to differences in the variabilities of the overall cell cycle period, G1 and S-G2-M phase durations (Figure S3). Intriguingly, our simulations predict that increasing the CV values for all the log-normal distributions for the transcription rates leads to greater Pearson’s correlation (See STAR Methods Section) in all lineage pairs (Figure 2D(ii)). This increase in Pearson correlations can be attributed to the greater linear rise of the covariance term over the increase in variances of cell cycle period and phase durations (Figure S3) as depicted by Equations 11 and 12 (STAR Methods Section). This suggests that for cancerous cells with a greater possibility of transcription rate variability across cellular populations, we will observe more correlated cell cycle durations in the lineage pairs.

At this point, we further analyze the effect of the additional sources of variability alongside the correlated transcriptional fluctuations. For example, we have performed simulation by randomly changing the initial conditions of the cell cycle regulatory genes (Figure S4) and found that this does not alter the correlation patterns as observed earlier (Figures 2A–2D). This suggests that our simulation results are quite robust and are independent of the initial levels of the cell cycle regulators in the newly born daughter cells. Additionally, in our algorithm, we have considered the transcription rate variations during mitosis (as described in the model section), which quantitatively accounts for the epigenetic modifications occurring during sister chromatid separation among the two daughter cells.^23^^,^^24^^,^^38^^,^^39^ Our simulations suggest that the transcription rate resetting during mitosis increases the variances in cell cycle period and phase durations (Figure S5) in a similar manner when we picked the transcription rates of various transcripts from a distribution with higher CV values (Figure S3). Thus, the transcriptional resetting also affects the lineage level correlations and brings it closer to experimentally observed D-D, M-D, and C-C correlations. Interestingly, we found that the exclusion of transcriptional resetting in our simulation does not affect the D-D correlation appreciably; however, it increases the M-D and C-C correlations significantly and brings both of these correlations to the level of D-D correlations, which is not observed in the experiments (Figure 2E). This foretells that such kind of random transcriptional variation during mitosis plays an essential role in reducing the M-D and C-C correlations without influencing the high D-D correlations by making the cell cycle dynamics of two daughter cells different (and also from their respective mother) during the mitosis.

Thus, our model simulations provide qualitative insight into how the fine-tuning of the transcriptional memory across lineage and during the cell cycle generates diverse lineage-level correlation patterns. Importantly, our simulation study reveals that the effect of autocorrelation time in transcriptional memory is more profound in D-D and M-D correlations than in C-C correlations (Figure 2A) which ultimately explains the origin of CMI (Figure 2D). This is quite a non-intuitive model prediction.

### Lineage-level correlation decreases with increasing cell cycle duration leading to an extended region of CMI

In Figure 2, simulations are performed assuming a ∼24-h cell cycle duration. However, the cell cycle duration in mammalian cells can vary from ∼16 to 40-h, which can lead to distinct correlation signatures. Herein, we investigate how alterations in cell cycle duration can influence these correlation patterns under specific transcriptional memory-related parameters.

**Figure 3:**
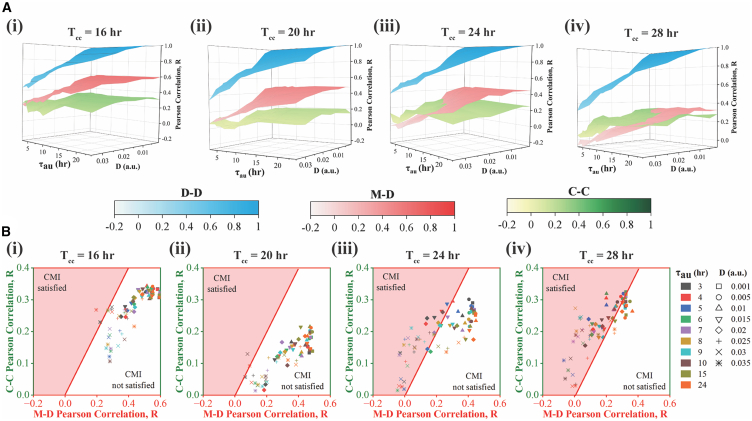
Lineage pair correlation patterns for varied cell cycle durations (A) Surface plots of cell cycle duration correlation patterns (D-D, M-D, and C-C) as a function of $\tau_{au}$ and D for (i) 16-h, (ii) 20-h, (iii) 24-h, and (iv) 28-h cell cycle period. (B) M-D vs. C-C correlation plot as a function of $\tau_{au}$ and D for (i) 16-h, (ii) 20-h, (iii) 24-h, and (iv) 28-h cell cycle period. All simulations are performed for 3 replicates (under each combination of D and $\tau_{au}$) each for 300 cells.

The simulation results indicate that correlation decreases among all lineage pairs as the cell cycle duration is increased monotonically from 16-h to 28-h maintaining a proportional increase in G_1_ and S-G_2_-M phase durations (Figure 3A). This drop in correlation occurs because the effect of transcriptional memory diminishes with increasing cell cycle durations. These findings qualitatively corroborate the experimental study by Kuchen et al.^14^ where the authors prolonged the cell cycle duration by suppressing Myc expression in neuroblastoma cells, which resulted in a decrease in the correlation of lineage pairs. Intriguingly, Figure 3 depicts that for cells having shorter cell cycle periods (16-h or 20-h, Figure 3A(i-ii)), the M-D correlation consistently remains higher than the C-C correlation, and **CMI** (Figure 3B(i-ii)) is almost non-existent. However, for cell types having longer cell cycle durations (24-h or 28-h, Figure 3A(iii-iv)), **CMI** appears (Figure 3B(iii-iv)) for a wider range of D and $\tau_{au}$.

Overall, our simulations foretell that in cell types with higher cell cycle durations, all the lineage correlations will decrease. However, the relative decrease in M-D correlation will be higher, which causes the distinct appearance of **CMI**.

### Modulation in G_1_ and S-G_2_-M phase durations alters lineage-level correlation patterns

**Figure 4:**
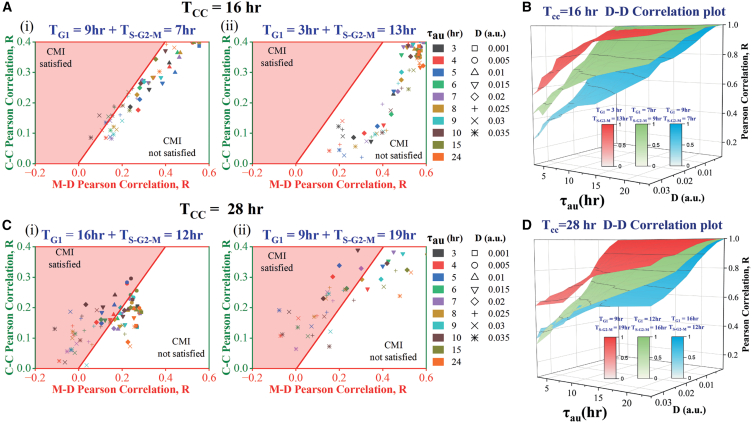
Change in the relative proportion of G_1_ and S-G_2_-M phase durations alters the correlation patterns (A) The plot of M-D and C-C correlations for a cell cycle duration of 16-h with G_1_ and S-G_2_-M durations of (i) 9-h and 7-h, and (ii) 3-h and 13-h, respectively. (B) D-D correlation plots with varying $\tau_{au}$ and D for different proportions of G_1_ and S-G_2_-M phases for a 16-h cell cycle period. (C) Plot of M-D and C-C correlations for a cell cycle duration of 28-h with G_1_ and S-G_2_-M durations of (i) 16-h and 12-h, and (ii) 9-h and 19-h, respectively. (D) D-D correlation plots with varying $\tau_{au}$ and D for different proportions of G_1_ and S-G_2_-M phases for the 28-h cell cycle period. All simulations are performed for 3 replicates (under each combination of D and $\tau_{au}$) each for 300 cells.

In the previous section, we explored how variations in cell cycle duration impact heterogeneity among cell lineage pairs by proportionately adjusting the G_1_ and S-G_2_-M phase durations to vary the cell cycle period. How does the alteration in G_1_ and S-G_2_-M phase durations for a fixed cell cycle period influence the lineage-level correlation patterns? We investigated this question by performing a simulation for two representative cell cycle periods (16-h and 28-h) with two different combinations of phase durations (low G_1_ and high S-G_2_-M, and high G_1_ and low S-G_2_-M). Our findings indicate that an extended S-G_2_-M phase (independently of the cell cycle period) increases the M-D correlation (Figures 4A and 4C). This phenomenon is due to mothers spending more time with the same set of transcription rates that are subsequently inherited by daughter cells following transcription rate variations at late M-phase. In contrast, cousin correlation remained unaffected by the variation in phase durations for shorter cell cycle periods (Figure 4A); however, with increasing S-G_2_-M duration for the longer cell cycle period, the C-C correlation seems to be increasing (Figure 4C). Importantly, the extent of **CMI** decreases (Figure 4A) with increasing S-G_2_-M phase duration for a 16-h cell cycle duration but it reduces marginally (Figure 4C) for a 28-h cell cycle period.

Further, we have observed that for a fixed cell cycle period, shorter S-G_2_-M duration produces a phase plane distribution having a condensed set of C-C and M-D correlation points (Figures 4Ai and 4Bi). This observation is due to the correlations in lineage pairs being less affected by the variations in $\tau_{au}$ and D. As the S-G_2_-M duration increases, the C-C and M-D correlation points in the phase plane become more dispersed (Figures 4A(ii) and 4B(ii)). Accordingly, our model simulations predict that the longer S-G_2_-M phase duration causes greater M-D correlation and destroys the **CMI** (Figures 4A and 4C).

**Figure 5:**
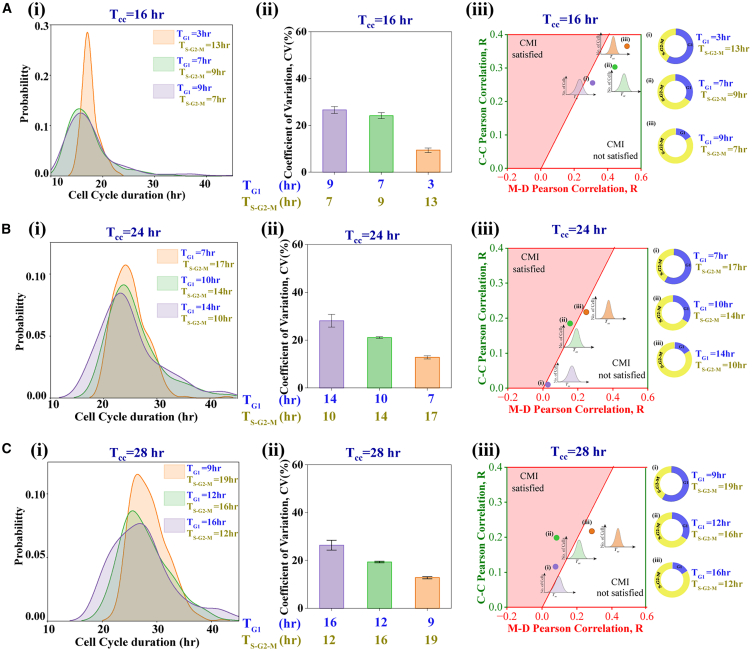
Lineage-level correlations pattern changes in a correlated manner with the population level heterogeneities in cell cycle duration as a function of extended S-G_2_-M phase durations with a fixed cell cycle period The population level ((i) cell cycle period distributions and (ii) Coefficient of variation of the cell cycle period distributions) and lineage level ((iii) plot of C-C Vs. M-D correlations) heterogeneities are quantified by varying the extent of S-G_2_-M phase durations for 3 different cell cycle periods ((A) 16-h, (B) 24-h, and (C) 28-h). The error bar in the middle panel represents the standard deviation of 3 replicates.

Interestingly, the D-D correlation increases with increasing S-G_2_-M phase duration independent of the overall cell cycle period (Figures 4B and 4D). Our simulations reveal that higher S-G_2_-M phase duration (with a fixed cell cycle duration) is indeed lowering the overall variabilities in cell cycle durations at the population level (discussed later in Figure 5), which leads to the enhancement in the D-D and M-D correlations and reduces the **CMI** (Figure 4). These model predictions can be verified experimentally by altering specific phase durations under various stress conditions by following the protocol as proposed by Chao et al.^40^

## Discussion

Identifying the factors regulating the cell cycle duration heterogeneities in mammalian cells is an important and challenging task. In this regard, recent studies at the single-cell level have provided varied explanations for the lineage-level heterogeneities.^10^^,^^11^^,^^12^^,^^13^^,^^14^^,^^17^^,^^18^ Here, we have modified and analyzed a simple generic cell cycle network^25^ model to understand the origin of lineage-level cell cycle duration heterogeneities in mammalian cells.

Employing our modeling analyses, we have been able to address some of the important open-ended questions in the field of cell cycle duration heterogeneity at the lineage level. First, our model unravels that the correlated variation of the inherited transcriptional rates across generations^21^^,^^22^^,^^30^ and its modulation during cell cycle progression is sufficient to qualitatively capture most of the experimentally observed^12^^,^^13^^,^^21^^,^^22^^,^^30^ lineage level correlation patterns (Figure 2) in cell cycle durations without considering any other biological factors. Intriguingly, the model predicts that cell types having higher transcriptional variability across generations (for example, cancerous cell lines or stem cells that are prone to differentiate) will have a greater probability of showing stronger cell cycle duration correlations in lineage pairs (Figure 2D). Thus, a correlated variation of the inherited transcriptional rates makes the cell cycle duration in daughter pairs highly correlated (Figures 2A and 2B); however, the modification of the transcription rates during mitosis^38^^,^^41^ makes M-D and C-C pairs less correlated than D-D pairs (Figures 2D and 2E). Importantly, depending on the nature and extent of the correlated fluctuations of the inherited transcription rates, the CMI as observed in experiments^10^^,^^11^^,^^12^^,^^14^^,^^17^^,^^18^ can be realized in our simulations (Figures 2A and 2B). This suggests that C-C cell cycle duration correlation can still exist due to subtle adjustments in the correlated transcriptional fluctuations during cell cycle progression even if the M-D correlation is low.

Our model further substantiates that the variation in the correlated transcriptional fluctuations fine-tunes the extent of CMI for different mammalian cells^11^^,^^14^^,^^17^ with varied cell cycle duration (Figure 3). It demonstrates that cell types with higher cell cycle periods will show a reduced level of correlation in all lineage pairs (Figure 4).^14^ This is because the inherited transcriptional memory will diminish to a greater extent in these cells due to longer cell cycles, however, it will extend the region of **CMI**. Finally, our model analysis elucidates that a higher extent of S-G_2_-M phase duration will not only increase the D-D correlations but it will also lower the extent of **CMI** by increasing the M-D correlations (Figure 4). These model predictions can be easily tested by employing similar experiments as performed by Chao et al.^40^

We further tried to investigate how the variation in the lineage-level correlation patterns (for example, the existence of **CMI**) be utilized to identify the variabilities in the proliferation pattern at the population level of mammalian cells.

Our simulations considering 3 different mean cell cycle durations (Figures 5A (16-h), 5B (24-h), and 5C (28-h)) indicates that the variabilities in the overall cell cycle duration (${CV}_{T_{cc}})$ decreases (Figures 5Ai, 5Aii, 5Bi, 5Bii, 5Ci, and 5Cii) if the S-G_2_-M phase duration is somehow increased maintaining a fixed cell cycle duration. This idea aligns with experimental findings by Govindaraj et al., who used the p38 inhibitor, which elongates the S-G_2_-M phase and reduces overall heterogeneity in cell cycle duration. Importantly, Our simulations further unravel that the relative increase in the M-D correlation is becoming systematically higher than the C-C correlations (Figures 5A(iii), 5B(iii), and 5C(iii)) which indicates a lowering of **CMI** without much alterations in D-D correlations, as the S-G_2_-M phase duration is increased with fixed cell cycle period. This suggests that such kind of lineage-level correlation change can be used as an indicator to figure out how to reach a situation therapeutically where the variabilities for the cell cycle durations can be systematically altered by perturbing specific physiological conditions before implementing the chemotherapeutic treatment. These predictions can be experimentally verified by altering specific phase durations under different stress conditions.^40^

At this point, one might ask a few relevant questions related to our model assumptions. For example: (1) What is the role played by the variation in the GF introduced in this model? (2) Is it necessary to consider the explicit dynamics of the mRNAs for all the transcripts in the model? Can we consider a simple model, where we introduce the correlated protein synthesis rates and get similar results? To answer the first question, we varied the GF levels in our simulations to assess its effect on lineage-level correlations. We observe that increasing levels of GF slow down the mean cell cycle period to either 23.5-h (GF = 5 a.u.) or 23-h (GF = 10 a.u.) from 24.8-h (GF = 2 a.u.). While the correlation in all lineage pairs increased due to the reduced cell cycle duration, the qualitative pattern of lineage correlations remains largely unaffected (Figure S6) as shown in the earlier experimental studies.^11^ To investigate the 2^nd^ question, we simulated the cell cycle dynamics by incorporating colored noise in the production rates of all the proteins without considering explicitly the mRNA dynamics (Tables S5–S7; Figure S7). However, we have not been able to capture the **CMI** phenomenon (Figure S7B) when we ignored the effect of the correlated transcription rate in the presence of explicit mRNA dynamics of the cell cycle regulatory genes in our model. Moreover, higher variability in the translation rate resetting, made the M-D and C-C correlations go down drastically in comparison to D-D correlations (Figure S7B). Whereas, even under higher variabilities in transcription rate resetting, the **CMI** phenomenon still holds good (Figure S7C). This suggests that our simple cell cycle model inclusive of the mRNA dynamics is more suitable to account for the experimentally observed lineage-level heterogeneities in mammalian cells.

Often circadian rhythm is linked to lineage correlation patterns observed in cell cycle durations.^10^^,^^17^^,^^18^ This hypothesis has its limitations. For example, the effect of circadian oscillation was shown in specific cell lines^10^^,^^17^ where inferences are mostly drawn via statistical models instead of network-based mathematical models. Recently, Nikhat et al.^42^ proposed a coupled model of the cell cycle and circadian regulatory network and showed that the origin of lineage pair correlation patterns might function through circadian control. Their model showed the presence of **CMI** by demonstrating cousin-cousin correlation can be higher than the mother-daughter correlation. However, why under certain circumstances **CMI** is not observed has not been explained by their model as it was dealing with a cell-type-specific observation. Further, these studies have only shown the lineage correlation patterns for some specific cell cycle duration which does not provide any scope to study the effect of the cell cycle duration variability in the context of lineage pair correlation and how under such a scenario coupling of cell cycle with circadian rhythm helps in modifying such correlations. Moreover, the strong dependence of circadian on the cell cycle is often altered in many MYC-driven cancers,^43^^,^^44^^,^^45^^,^^46^ which still exhibit lineage correlation patterns in cell cycle durations.^14^^,^^47^ It remains unclear how to rationalize the existence of lineage pair correlation patterns in these cell lines.

In contrast, our model adopts a simple network-based mathematical model approach and puts forward a simple and more generic concept of correlated transcriptional activity which eventually governs the lineage pair correlations in cell cycle duration for mammalian cells. Importantly, our model explains the CMI generically and demonstrates how changes in the cell cycle period and even the phase durations alter the lineage pair correlations in a specific way. These predictions from our model seem quite in line with experiments performed with various mammalian cell types in the existing literature.^10^^,^^11^^,^^12^^,^^14^ We have achieved this just by considering the idea of correlated transcriptional fluctuation which seems to be a ubiquitous phenomenon happening in all cell types of different origins in comparison to the circadian cycle that may or may not influence cell cycle regulation depending on the context.

Overall, our modeling study exhibits that the inheritance of the transcription rates across cell lineages and their correlated fluctuations during cell cycle progression can explain the diverse lineage-level correlation patterns in mammalian cells. We believe that these insights will be useful in modifying the cell cycle duration heterogeneities and finding therapeutic relevance.

### Limitations of the study

Like any other modeling study, our work also has certain limitations. First, our model and analysis are dependent on the hypothesis that the transcriptional rates are getting modulated across generations and during cell cycle progression in a correlated manner. However, it is hard to provide a physiological interpretation of the quantities like the noise strength (D) and correlation time ($\tau_{au}$) which have been used to model the correlated nature of the transcription in our model. This makes experimental realization of our model predictions a difficult proposition, however, performing experiments to prove our model are conceivable. Second, due to its simple nature, the model fails to quantitatively obtain the exact values of the correlations in a cell-type-specific manner (for example, high C-C and no M-D correlation in case of L1210 cell type.) However, it provides a qualitative explanation under varied cell cycle periods and phase durations which can be tested experimentally. Third, our attempt to connect the lineage-level and population-level heterogeneities still needs more attention as it is an important aspect of this study, however, due to the lack of key regulators in our model which govern the precise durations of G_1_ and S-G_2_-M phases, we are unable to get greater insight from this model. The initial model simulations in this direction (Figure 5) seem promising and we require further in-depth studies to prove our hypothesis more comprehensively.

## Resource availability

### Lead contact

Further information and requests for resources should be directed to and will be fulfilled by the lead contact, Dr. Sandip Kar (sandipkar@iitb.ac.in).

### Materials availability

This study did not generate any new reagents.

### Data and code availability

• All codes used in this manuscript are available at: https://github.com/Kajalcharan27/Cell-cycle-colored-noise.git.
• Any additional information required is available from the lead contact upon request.

## Acknowledgments

Thanks are due to UGC for providing the UGC-CSIR-JRF (NTA Ref. No: 191620004555) fellowship to KC. This work is supported by the funding agency 10.13039/501100001843SERB, India (Grant no. CRG/2023/002165). We thank Dr. Amitava Giri and Dr. G. Vinodhini for their suggestions during various stages of this study.

## Author contributions

K.C. and S.K. designed the research problem together. K.C. developed the mathematical model and simulation methodology with valuable input from S.K. K.C. conducted the simulations and improved the codes. K.C. and S.K. subsequently interpreted the results to make predictions and jointly wrote the manuscript. S.K. supervised the overall work.

## Declaration of interests

The authors declare no competing interests.

## STAR★Methods

### Key resources table

**Table:** 

REAGENT or RESOURCE	SOURCE	IDENTIFIER
**Software and algorithms**		

MATLAB 2020b	The MathWorks	N/A
XPPAUT	https://sites.pitt.edu/∼phase/bard/bardware/xpp/xpp.html	N/A
Lineage Simulation	This Paper	https://github.com/Kajalcharan27/Cell-cycle-colored-noise.git

### Method details

#### Model construction

In our minimalistic mathematical model adapted from Tyson et al. 2001,^25^ we have modified the original network by incorporating the mRNA of each protein and making its growth factor (GF) dependent. The model has three regulatory components of the cell cycle: CycB, Cdh1, and Cdc20. In the cell cycle, Cdh1/APC is expressed during the G1 phase, while CycB/CDKs, the activators of the cell cycle, remain high during the S, G2, and M phases of the cell cycle. We have translated all the molecular interactions depicted in Figure S1A into ordinary differential equations (Equations 2, 3, 4, 5, 6, 7, 8, 9, and 10 in Table S2). Equation 2 shows the dynamics of CycB and cell cycle initiation by growth factor. Here we have included the correlated fluctuations in the transcription rate of CycB mRNA (1^st^ term in Equation 2) and its normal degradation (2^nd^ term in Equation 2).

(Equation 2) $$\frac{dCycBm}{dt}=\frac{\left(k_{1m}+\varepsilon_{ou1}\right)\times GF}{K_{mm}+k_{eff}*GF}-k_{1dm}\times CycBm$$

Equation 3 shows the dynamics of translation of CycB protein (1^st^ term in Equation 3), natural degradation (2^nd^ term in Equation 3), and Cdh1 mediated degradation of CycB (3^rd^ term in Equation 3).

(Equation 3) $$\frac{dCycB}{dt}=k_{1}\times CycBm-k_{2a}\times CycB-k_{2b}\times CycB\times Cdh1$$

Equation 4 captures the dynamics of Cdc20 mRNA; transcription is turned on by CycB independent (1^st^ term in Equation 4) and CycB dependent (2^nd^ term in Equation 4) with Hill function parameterized by J_5_ and n. Degradation of Cdc20 mRNA shown in last term of Equation 4.

(Equation 4) $$\frac{dCdc20m}{dt}=\left(k_{5am}+\varepsilon_{ou2}\right)+\frac{\left(k_{5bm}+\varepsilon_{ou3}\right)}{k_{5cm}+GF\times J_{5c}}\times \frac{CycB^{n}}{J_{5}^{n}+CycB^{n}}-k_{5dm}\times Cdc20m$$

Equations 5 and 6 show the total Cdc20 protein and Cdc20A dynamics, respectively. The concentration of total Cdc20, including both active and inactive forms, has translation and degradation terms Equation 5. Newly synthesized Cdc20 is initially inactive (Cdc20 total – Cdc20 active), then activated by IEP (Shown in Equation 10) with some intermediate steps (1st term in Equation 6). Cdc20 activation ensures the DNA synthesis and chromosome alignment before anaphase if they are not finished on time; then spindle checkpoint protein MAD will inactivate the Cdc20 (2^nd^ term in Equation 6)

(Equation 5) $$\frac{dCdc20T}{dt}=k_{5a}\times Cdc20m-k_{6}\times Cdc20T$$

(Equation 6) $$\frac{dCdc20A}{dt}=k_{7}\times IEP\times \frac{Cdc20T-Cdc20A}{J_{7}+Cdc20T-Cdc20A}-k_{8}\times Mad\times \frac{Cdc20A}{J_{8}+Cdc20A}-k_{6}\times Cdc20A$$

As we have modeled the Cdh1 mRNA in Equation 7, we incorporated the colored noise in the transcription rate (1^st^ term in Equation 7). The last term shows the natural degradation of Cdh1 mRNA.

(Equation 7) $$\frac{dCdhm}{dt}=\left(k_{3m}+\varepsilon_{ou4}\right)-k_{3dm}\times Cdhm$$

Equations 8 and 9 capture the total Cdh1 protein and active Cdh1 protein dynamics, respectively. Equation 8 includes a translation and a degradation term for total Cdh1 protein. Equation 9 has an activation and deactivation mechanism of Cdh1, which is modeled using Michaelis-Menten kinetics. The activation of Cdh1 is driven by Cdc20A (active form) (1^st^ term in Equation 9). Meanwhile, CycB deactivates the active form of Cdh1 through phosphorylation (2^nd^ term in Equation 9).

(Equation 8) $${\frac{dCdht}{dt}=k}_{3a}\times Cdhm-k_{3dt}\times Cdht$$

(Equation 9) $$\frac{dCdh1}{dt}=\left(k_{3}+k_{3b}\times Cdc20A\right)\times \frac{Cdht-Cdh1}{J_{3}+Cdht-Cdh1}-k_{4}\times CycB\times \frac{Cdh1}{J_{4}+Cdh1}-k_{3dt}\times Cdh1$$

Equation 10 includes the dynamics of the hypothetical intermediary enzyme (IEP); IEP generates a time lag between CycB activation and Cdc20A rise. The first term shows the activation of IEP by CycB where total concentration is scaled to 1. The last term depicts the inactivation of IEP.

(Equation 10) $$\frac{dIEP}{dt}=k_{9}\times CycB\times \left(1-IEP\right)-k_{10}\times IEP$$

The structure of these dynamical equations (Equations 2, 3, 4, 5, 6, 7, 8, 9, and 10) remains consistent throughout our simulations. Parameter values for the ODEs were directly taken from Tyson et al. 2001,^25^ and parameters related to mRNAs were chosen to match the protein time profiles from Tyson et al.^25^ (Table S3). To vary the cell cycle durations and cell cycle phase durations, we adjusted a few parameters from those listed in Table S3; these changes are provided in the notes below Table S3.

#### Simulation in cell lineages

The differential equations, parameter values, and initial conditions given in Tables S1–S3, respectively, are used for simulation. These differential equations are solved in MATLAB ode solver (ode15s). To follow cell lineages, we adapted the methodology similar to Govindaraj et al., where we performed simulations for 72-h up to three generations.^11^ At cell division (identified at some specific value of CycB), all variables were divided equally, and the simulation was performed for two cells with similar values.^25^

Cell cycle duration is calculated from the cell’s birth to its division time. To calculate the G1 phase duration, we added an additional variable X ($\frac{dX}{dt}=k_{11}-k_{12}\times CycB\times X-k_{13}\times X$). In our simulation, to calculate the precise G_1_-S transition, we considered X degradation by CycB. To precisely measure the G1 phase duration, we introduced this additional pseudo-variable X, which undergoes CycB-dependent degradation. As a result, X drops sharply as CycB levels increase. The duration of the G_1_ phase is then measured as the time interval from cell division to the peak of X. This indicates that the Cdh1 level is going down just after X reaches its peak and CycB levels will take over which we assumed as the end of G_1_ phase in our model consistently for all of our simulations.

All simulations were performed for 100 lineages, and the cells finishing their cell cycle before simulation time were considered. All pairs of daughter-daughter (D-D), mother-daughter(M-D), and cousin-cousin(C-C) cells were separated and used to calculate correlations from the initial 300 cells of each simulation.

#### Pearson correlation

In mathematics, Pearson correlation (abbreviated as R) is used to understand the strength and direction of the linear relation between two variables. It evaluates whether an increase in one variable is associated with either (i) an increase (positive correlation) or (ii) a decrease (negative correlation) in another variable. It is defined mathematically as:

(Equation 11) $$Pearson\;correlation\;\left(R\right)=\frac{Cov\;\left(X,Y\right)}{\sqrt{var\left(X\right)}\sqrt{var\left(Y\right)}}$$

Where the Cov(X,Y) is the covariance between X and Y, which means the degree to which the change in variable one (X) is associated with the change in another variable (Y). var(X) and var(Y) are the variances in the data associated with variables X and Y, respectively.

(Equation 12) $$Cov\;\left(X,Y\right)=\sum \frac{\left(X_{i}-\bar{X}\right)\left(Y_{i}-\bar{Y}\right)}{N-1}$$

N is the number of data points, and $\bar{X}$ and $\bar{Y}$ are the mean of X and Y datasets, respectively.

Pearson Correlation (R) is a nondimensional quantity which ranges from −1 to +1,

R=1; perfect positive correlation

R=0; No correlation

and R=−1, perfect negative correlation

In the context of biological systems, Pearson correlation measures the degree of correlated changes in two biological variables, such as gene expressions, protein concentrations, physiological measurements, etc. It is a powerful tool for exploring relationships and generating hypotheses about interactions and co-dependences in complex biological systems.

In our model, we utilized the concept of Pearson correlation after performing stochastic simulation for multiple cell lineages, and then we categorized lineage pairs, including Mother-Daughter (M-D), Daughter-Daughter (D-D), and Cousin-Cousin (C-C), within these lineages. Using the Pearson correlation function in MATLAB, we calculated the correlation in these lineage pairs (M-D, D-D, and C-C). The definition for Pearson correlation remains the same for all the concerned lineage pairs; only the corresponding cell cycle periods and phase durations are used while calculating the correlation coefficient R.

#### Significance analysis

We conducted a significance analysis to evaluate whether the observed correlations between specific lineage pairs were significantly different. Specifically, we compared the correlation coefficients (R) among various types of pairs— (i) D-D and M-D, (ii) D-D and C-C, (iii) M-D and C-C (Figures S8–S47)—across three individual replicates, each containing 300 cells. For each cell subpopulation in each replicate, we generated 500 bootstrap random samples and calculated the Pearson correlation coefficient (R). To assess the statistical significance, we applied Fisher’s z-transformation to the Pearson correlation coefficient values and then calculated *p*-values using the Student’s t-test.

The figures (Figures S8–S47) show *p*-value plots, where *p*-values less than 0.05 indicate statistically significant differences in the correlation coefficients of the lineage pairs; conversely, *p*-values greater than 0.05 suggest that the difference in correlation coefficients of lineage pairs are not statistically significant. All the plots in Figures S8–S47 are plotted using the sigstar function in MATLAB. (Rob Campbell (2025). raacampbell/sigstar (https://github.com/raacampbell/sigstar), GitHub. Retrieved March 8, 2025.) Our significance analysis shows that D-D correlations significantly differ from M-D and C-C correlations (*p* < 0.05). Moreover, in most cases, C-C and M-D pairs also show considerable differences (*p* < 0.05) in both scenarios where CMI is satisfied, and CMI is not satisfied.
