# Supplementary material for: Subtle alteration in transcriptional memory governs the lineage-level cell cycle duration heterogeneities of mammalian cells
Source: iScience. 2025 Jun 21;28(7):112981. doi: 10.1016/j.isci.2025.112981 (PMC12271079; doi:10.1016/j.isci.2025.112981)
Supplement: Document S1. Figures S1–S47 and Tables S1–S7 [file mmc1.pdf]

iScience, Volume *28*

Supplemental information

Subtle alteration in transcriptional memory governs the lineage-level cell cycle duration heterogeneities of mammalian cells

Kajal Charan and Sandip Kar

S1 Supplemental figures (S1-S47)

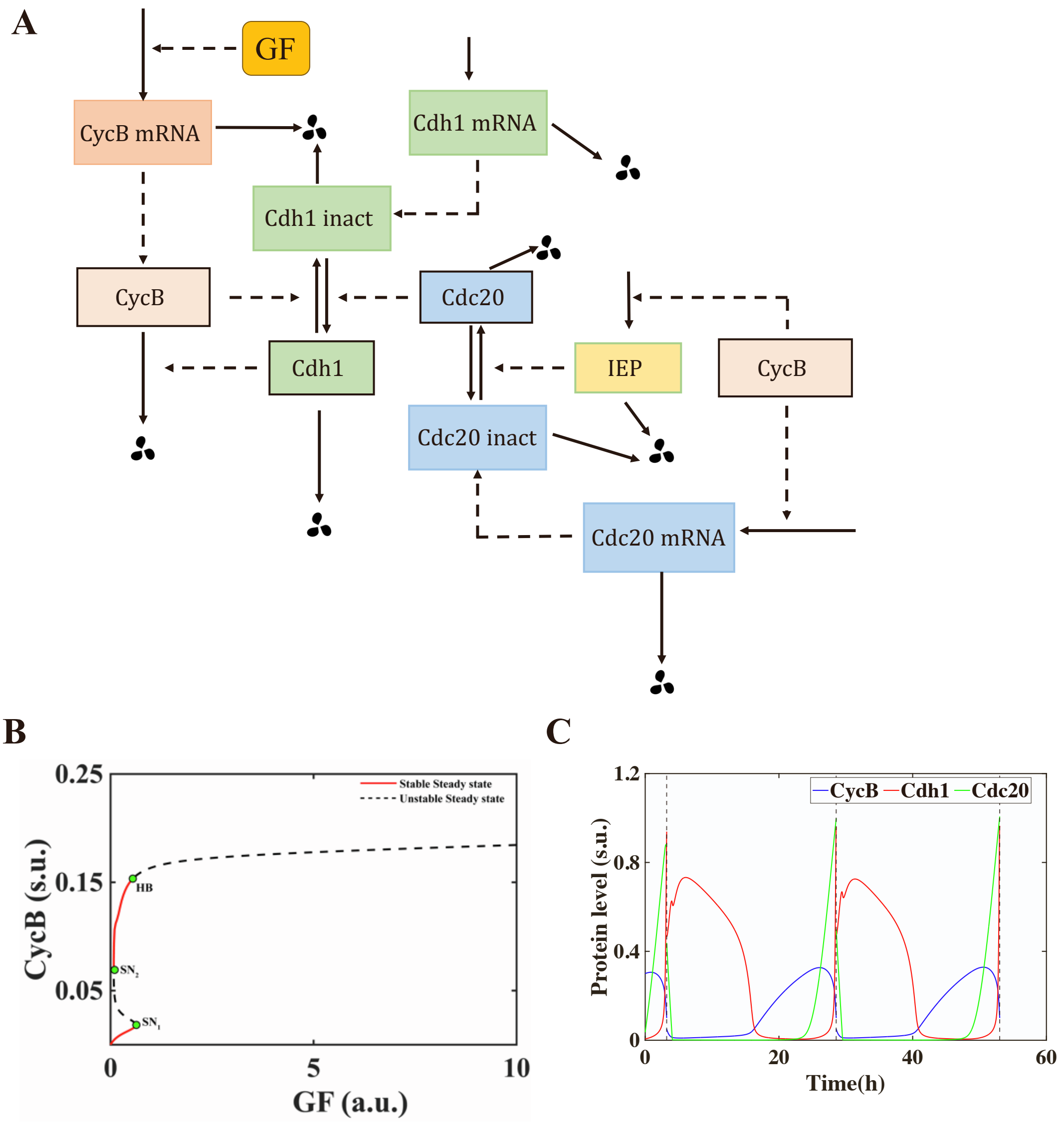

Figure S1. Detailed cell cycle regulatory network and SNIC bifurcation analysis (A) A detailed modified cell cycle regulatory network incorporating growth factor-dependent activation of CycB mRNA along with the mRNA of three key regulatory proteins, CycB, Cdh1, and Cdc20. CycB inhibits Cdh1 via phosphorylation, while Cdh1 facilitates CycB degradation. CycB indirectly activates Cdh1 by promoting Cdc20 activation, thereby completing negative feedback. Here, Dashed and solid arrows represent catalytic activation and biochemical conversion respectively. The solid outwards arrows signify the degradation of corresponding mRNA or protein. (original model adapted from Tyson et al. 2001)[2]. **(B)** Bifurcation diagram of CycB as a function of Growth factor (GF), where HB stands for Hopf Bifurcation point, and SN represents Saddle Node points. **(C)** Temporal dynamics of CycB, Cdh1, and Cdc20 proteins at GF=2. (Vertical dotted line shows the cell division event) (Related to Figure 1A)

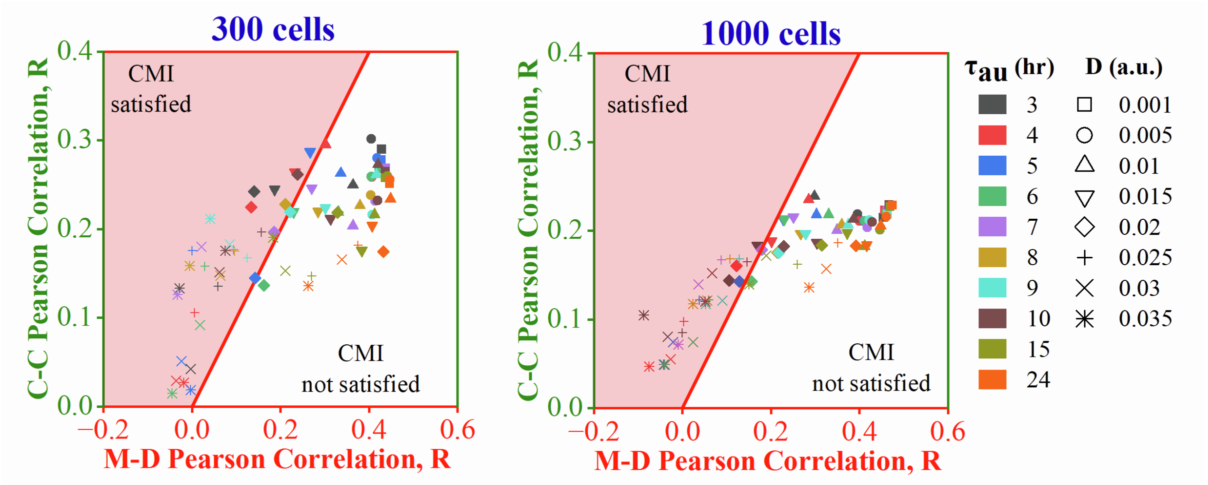

Figure S2. The plot of M-D cell cycle duration correlation as a function of C-C correlation for 300 cells (left) and 1000 cells (right) at different τ_{au} (3hr to 24hr) and different noise strengths $\boldsymbol{D}$ (0.001 to 0.035) (Cell cycle duration is ~ 24-hour here). (Related to Figure 2D)

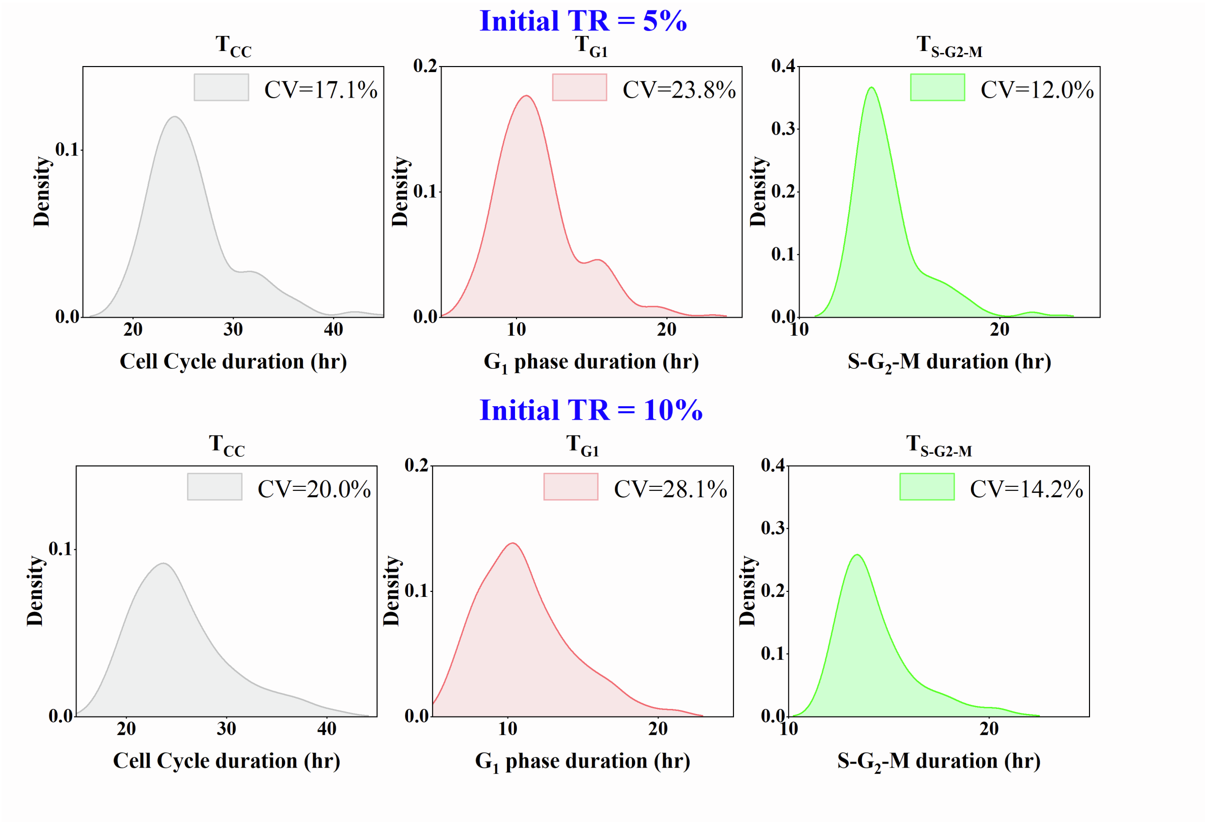

Figure S3. Cell cycle duration (T_{cc}), G_1 phase duration (T_{G1}), and S-G_2-M phase duration ($T_{S\text{-}G2\text{-}M}$) distribution for 5% CV of initial transcription rates (upper panel) and 10% CV of initial transcription rate (lower panel) (plot for $\boldsymbol{D}$ = 0.015, and τ_{au}=10-hour). (Related to Figure 2D)

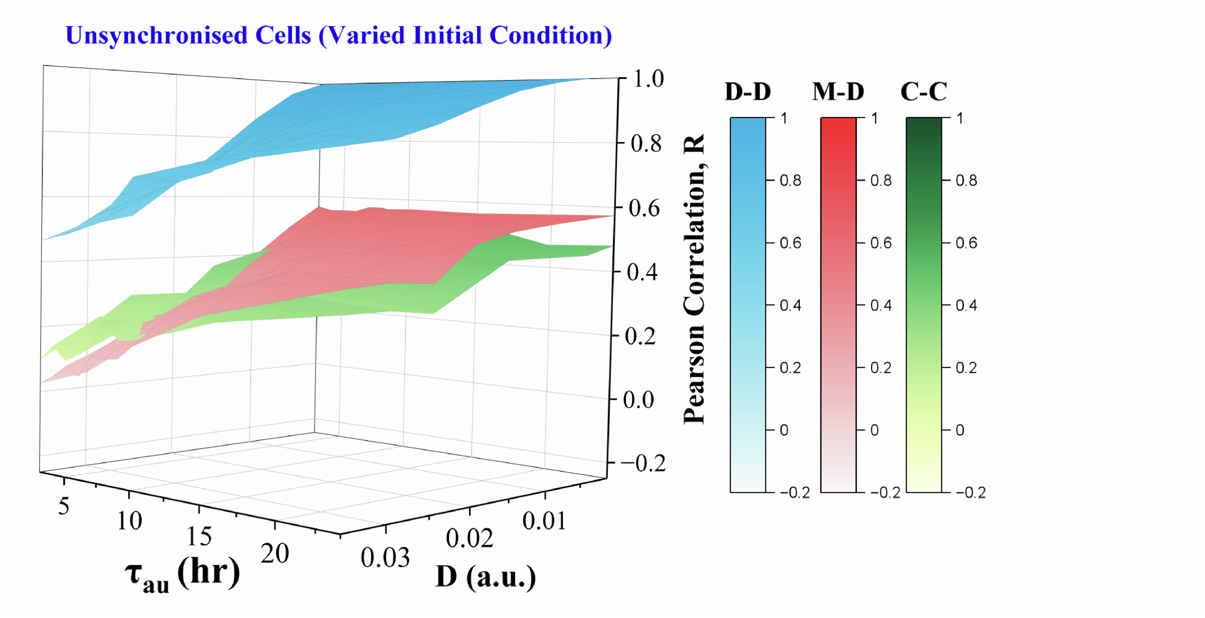

Figure S4. Surface plots of cell cycle duration correlation patterns (D-D, M-D, and C-C) as a function of $\boldsymbol{\tau_{au}}$ and $\boldsymbol{D}$ for cells with varied initial conditions. (Cell cycle duration is ~24-hour). (Related to Figure 2D)

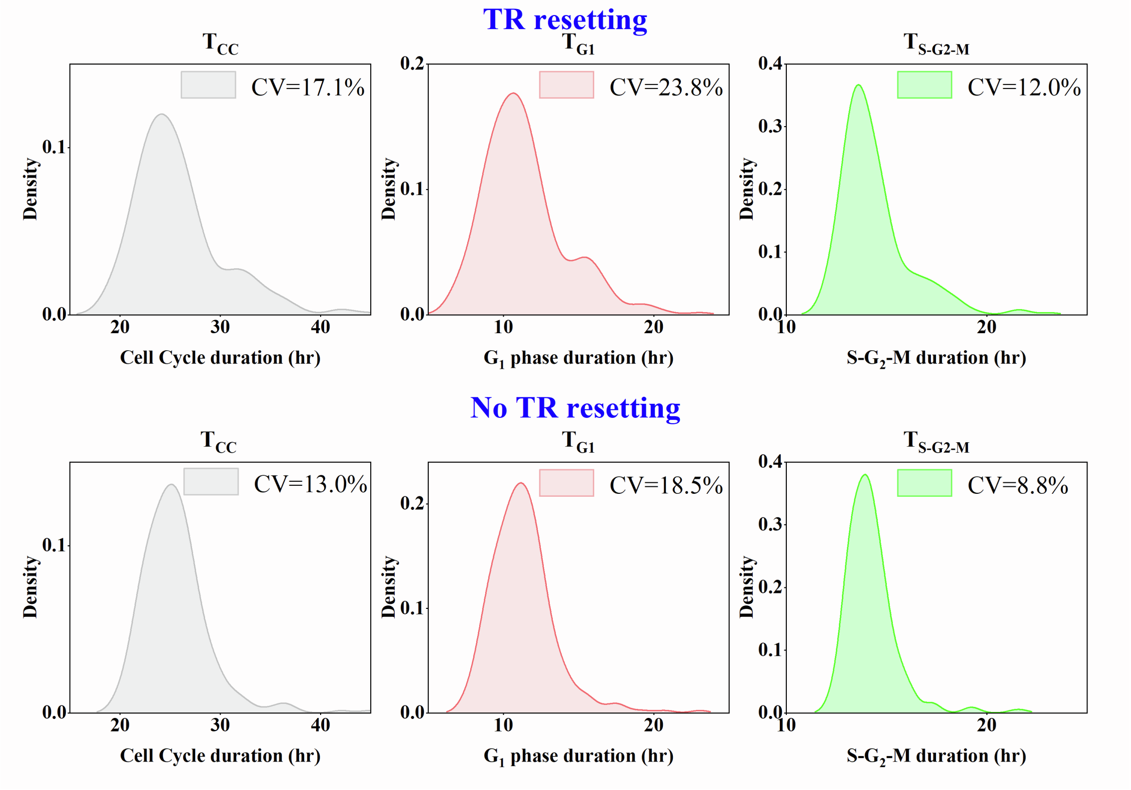

Figure S5. Cell cycle duration (Tcc), G_1 phase duration, and S-G_2-M phase duration distribution for Transcription rate resetting during mitosis (upper panel) and no transcription rate resetting (lower panel) ($\boldsymbol{D}$ = 0.015 and $\boldsymbol{\tau_{au}}$=10-hour). (Related to Figure 2D and 2E)

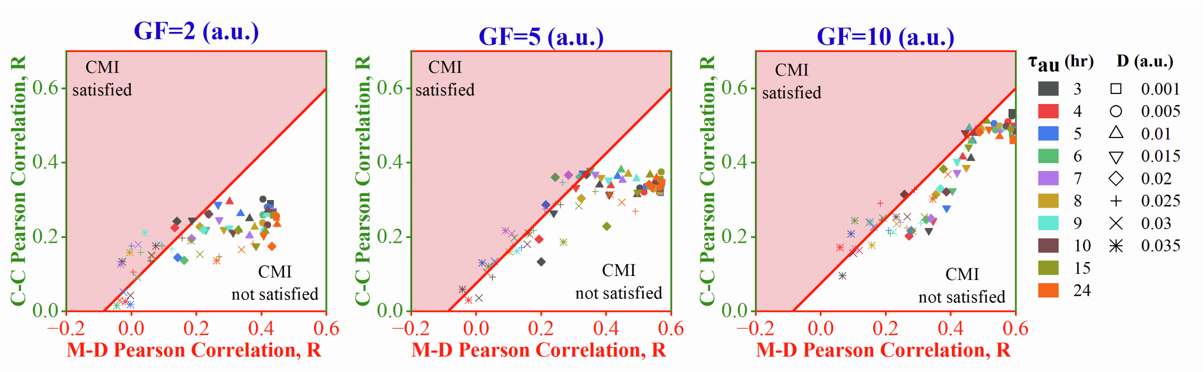

Figure S6. The plot of M-D cell cycle duration correlation as a function of C-C correlation for GF=2 (left), GF=5 (center), and GF=10 (right) at different $\boldsymbol{\tau_{au}}$ (3-hour to 24-hour) and different noise strengths $\boldsymbol{D}$ (0.001 to 0.035). (Related to Figure 2)

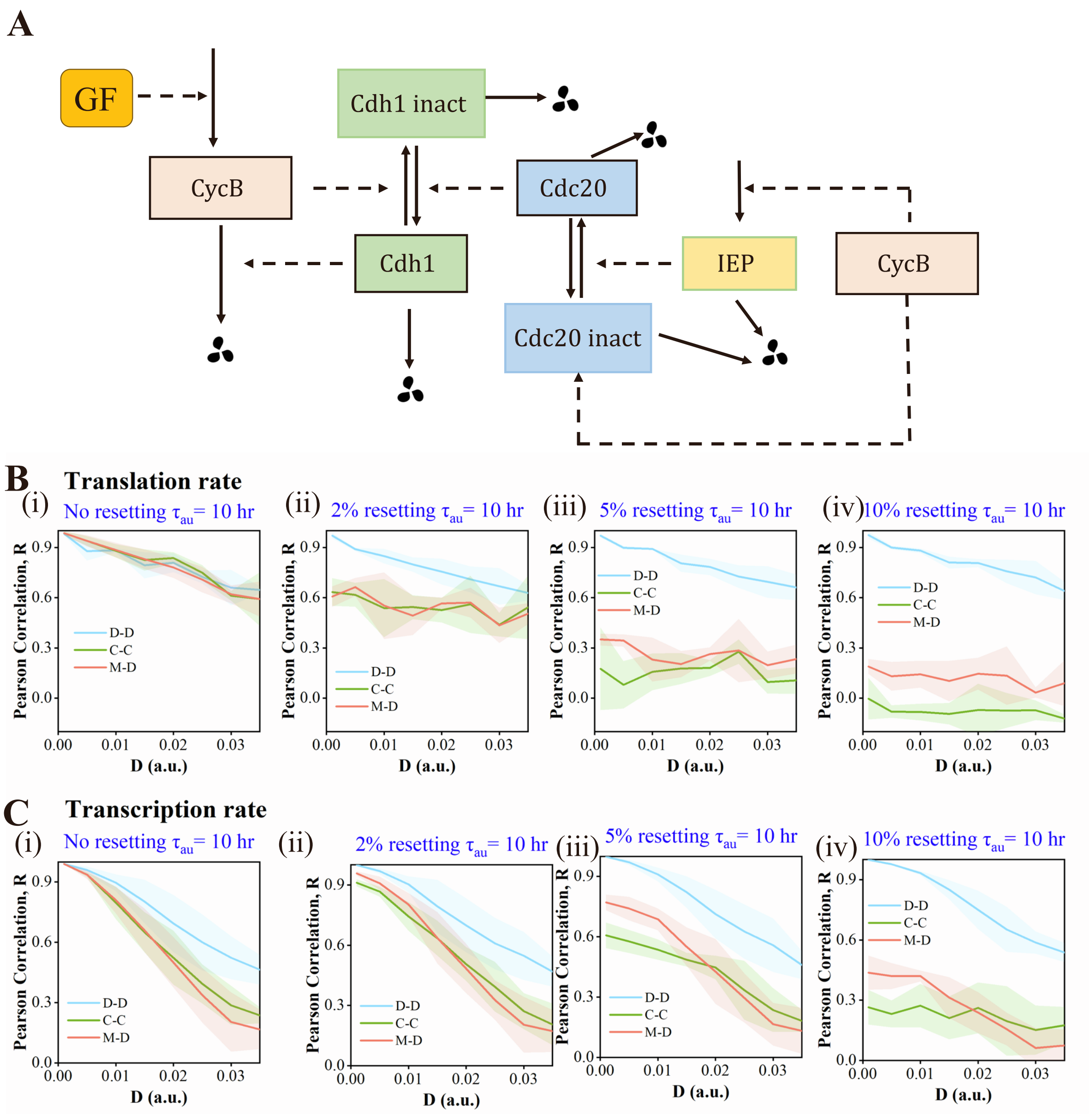

Figure S7. Correlation analysis in lineage pairs in the presence of translational noise (A) Detailed cell cycle regulatory network including growth factor induced activation of CycB. (adapted from Tyson et al. 2001). **(B)** Plot of Cell cycle duration correlation as a function of noise strength $\boldsymbol{D}$ at fixed $\boldsymbol{\tau_{au}}$ =10hr when the colored noise is added to the translation rate of proteins for different translation rate resetting values (i) no resetting (ii) 2% (iii) 5% and (iv) 10%. **(C)** Plot of Cell cycle duration correlation as a function of noise strength $\boldsymbol{D}$ at fixed $\boldsymbol{\tau_{au}}$ =10hr when the colored noise is added to the transcription rate of mRNAs for different transcription rate resetting values (i) no resetting (ii) 2% (iii) 5% and (iv) 10%. The error bar cloud in Figures S7B and S7C represents the standard deviation of 3 replicates. (Related to Figure 2)

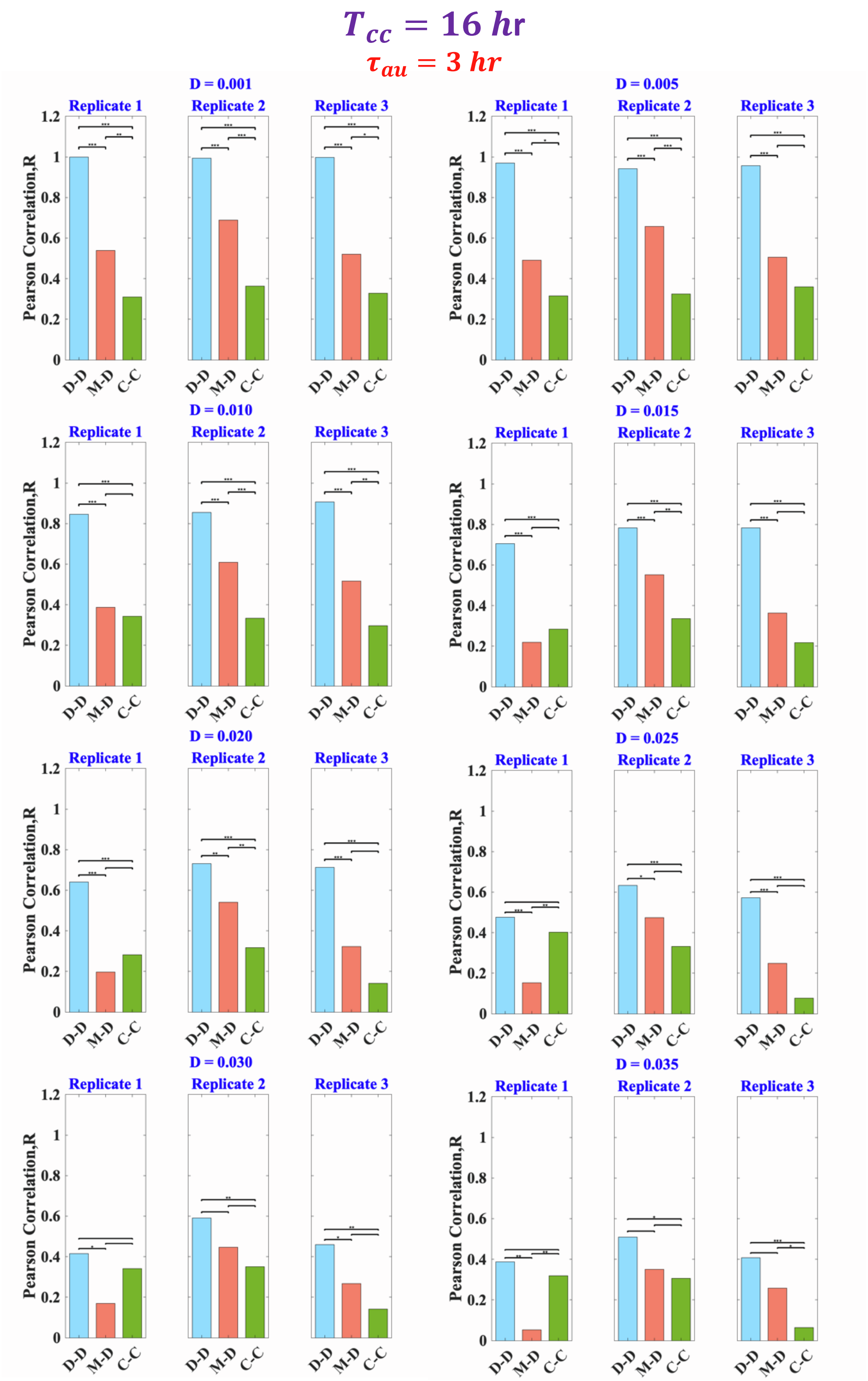

Figure S8 Significance analysis (Related to **Figure 3A(i) & 3B(i)**) of correlations between lineage pairs of individual replicates for τ_{au} value 3hr (***p<0.05**, ****p<0.001, ***p<0.0001**). p indicates the p-value calculated using a Fisher's z transformation of correlation values from lineage pairs. The total cell cycle time is **16 hours** here.

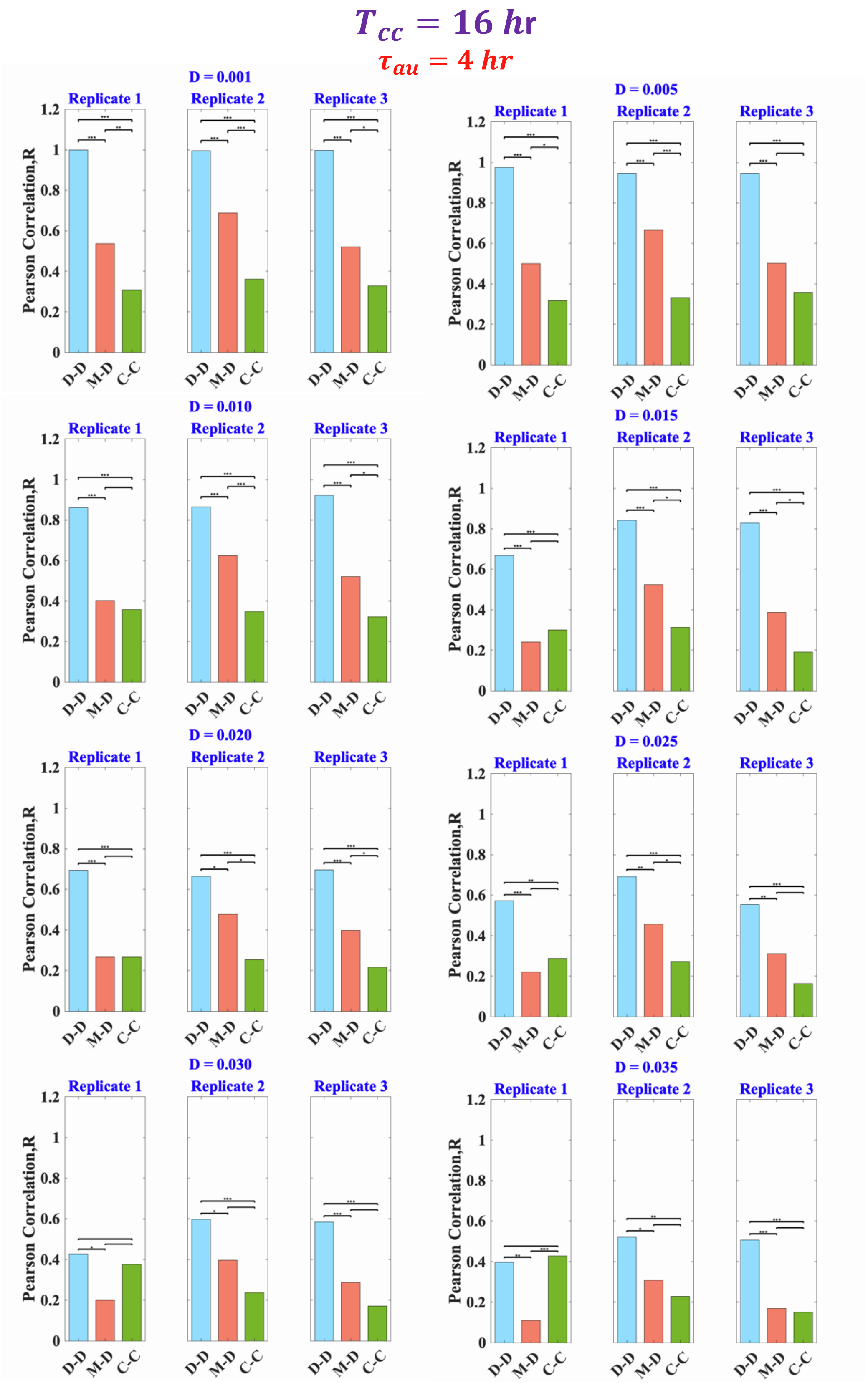

Figure S9 Significance analysis (Related to **Figure 3A(i) & 3B(i)**) of correlations between lineage pairs of individual replicates for τ_{au} value 4hr (***p<0.05**, ****p<0.001, *****p<0.0001**). p indicates the p-value calculated using a Fisher's z transformation of correlation values from lineage pairs. The total cell cycle time is **16 hours** here.

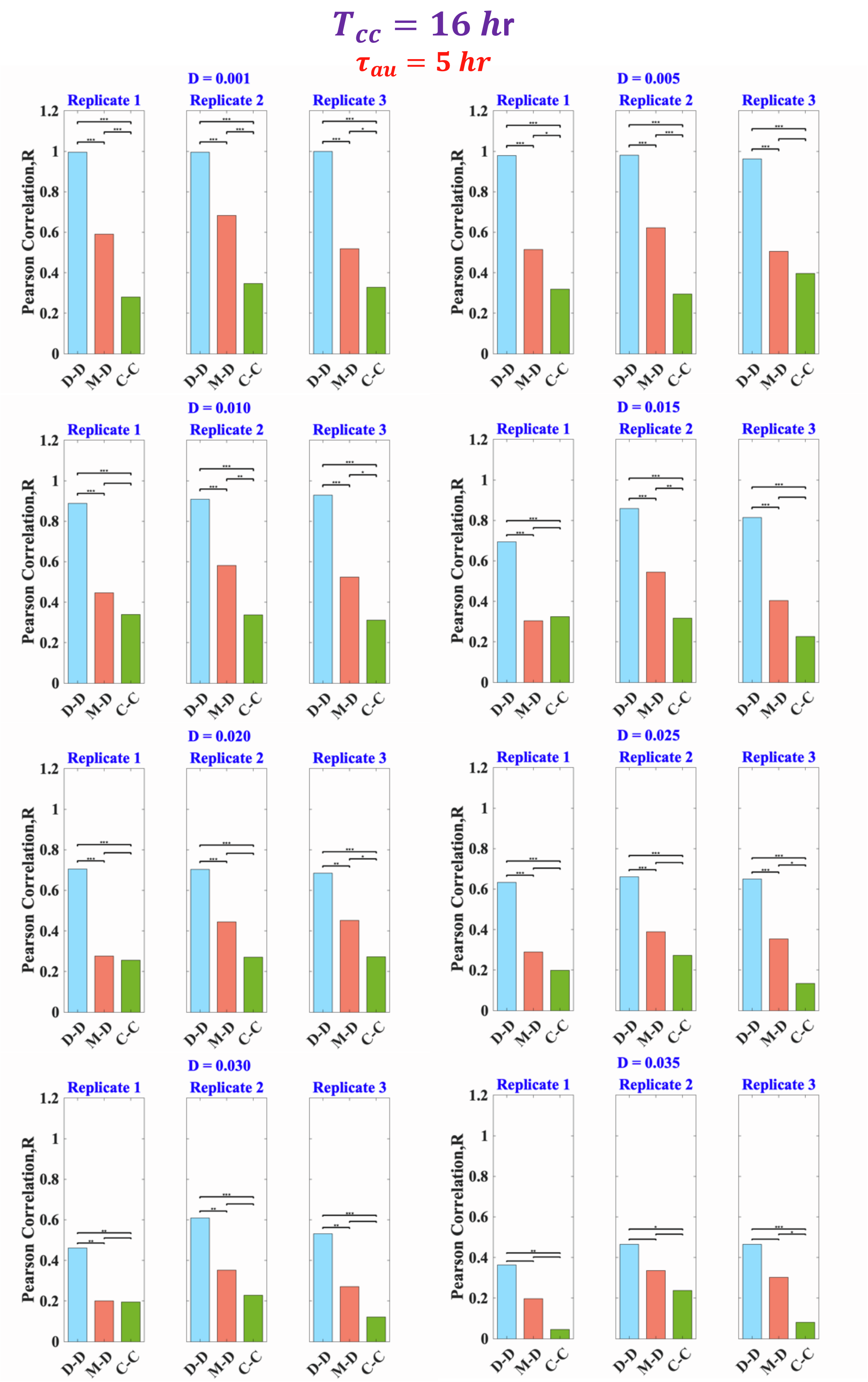

Figure S10 Significance analysis (Related to **Figure 3A(i) & 3B(i)**) of correlations between lineage pairs of individual replicates for τ_{au} value 5hr (***p<0.05**, ****p<0.001, ***p<0.0001**). p indicates the p-value calculated using a Fisher's z transformation of correlation values from lineage pairs. The total cell cycle time is **16 hours** here.

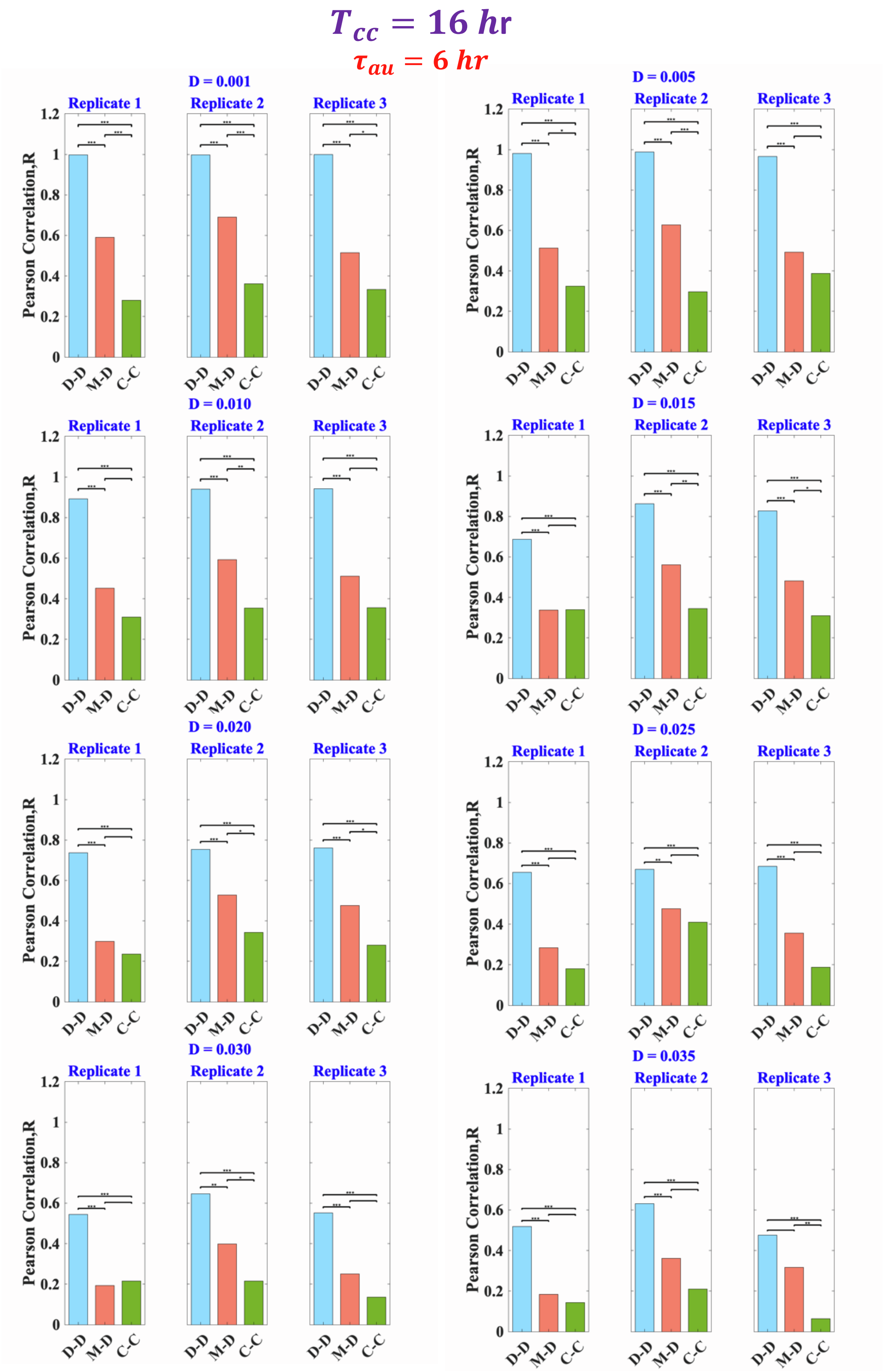

Figure S11 Significance analysis (Related to **Figure 3A(i) & 3B(i)**) of correlations between lineage pairs of individual replicates for τ_{au} value 6hr (***p<0.05**, ****p<0.001, ***p<0.0001**). p indicates the p-value calculated using a Fisher's z transformation of correlation values from lineage pairs. The total cell cycle time is **16 hours** here.

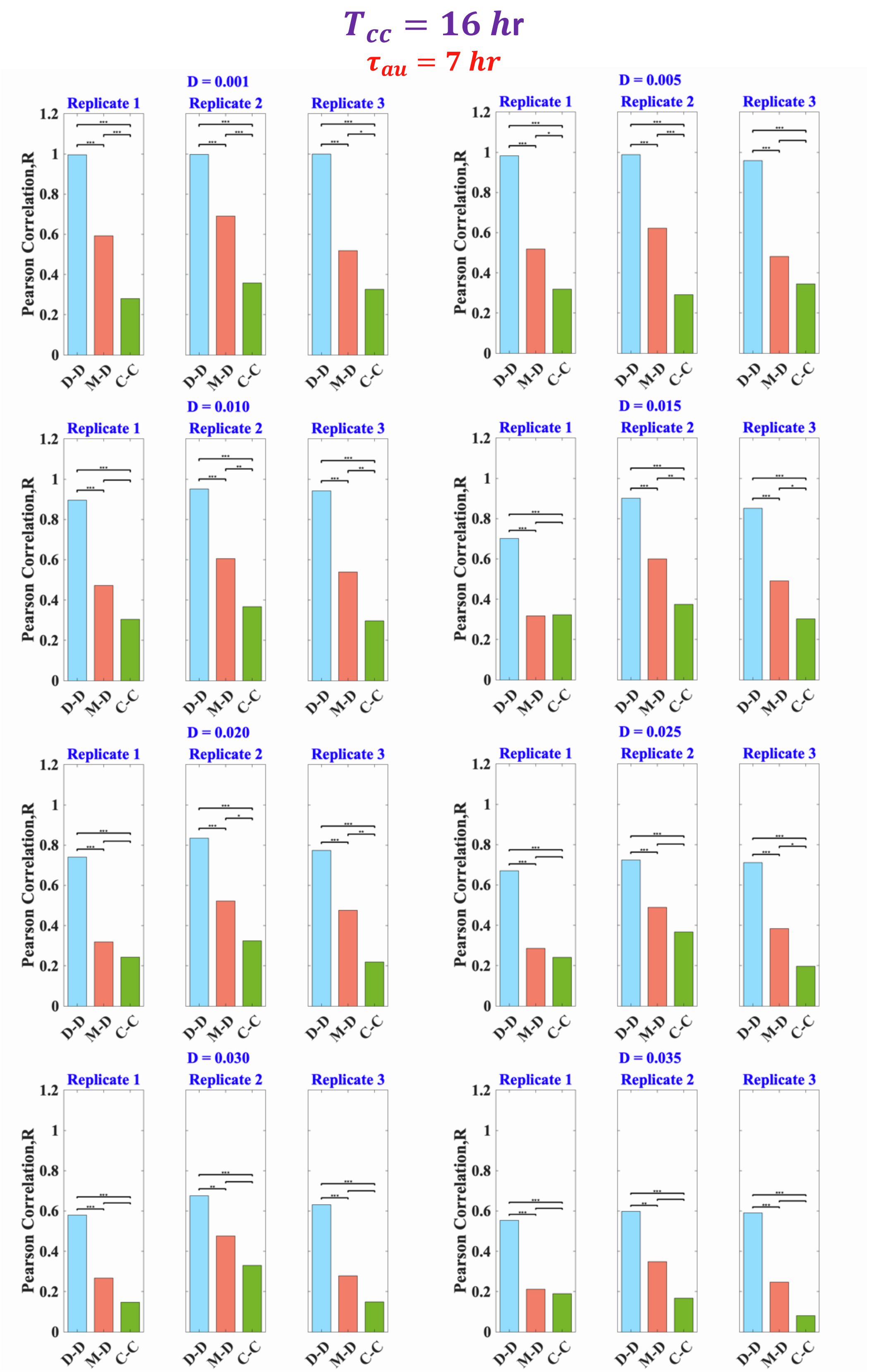

Figure S12 Significance analysis (Related to **Figure 3A(i) & 3B(i)**) of correlations between lineage pairs of individual replicates for τ_{au} value 7hr (***p<0.05**, ****p<0.001, ***p<0.0001**). p indicates the p-value calculated using a Fisher's z transformation of correlation values from lineage pairs. The total cell cycle time is **16 hours** here.

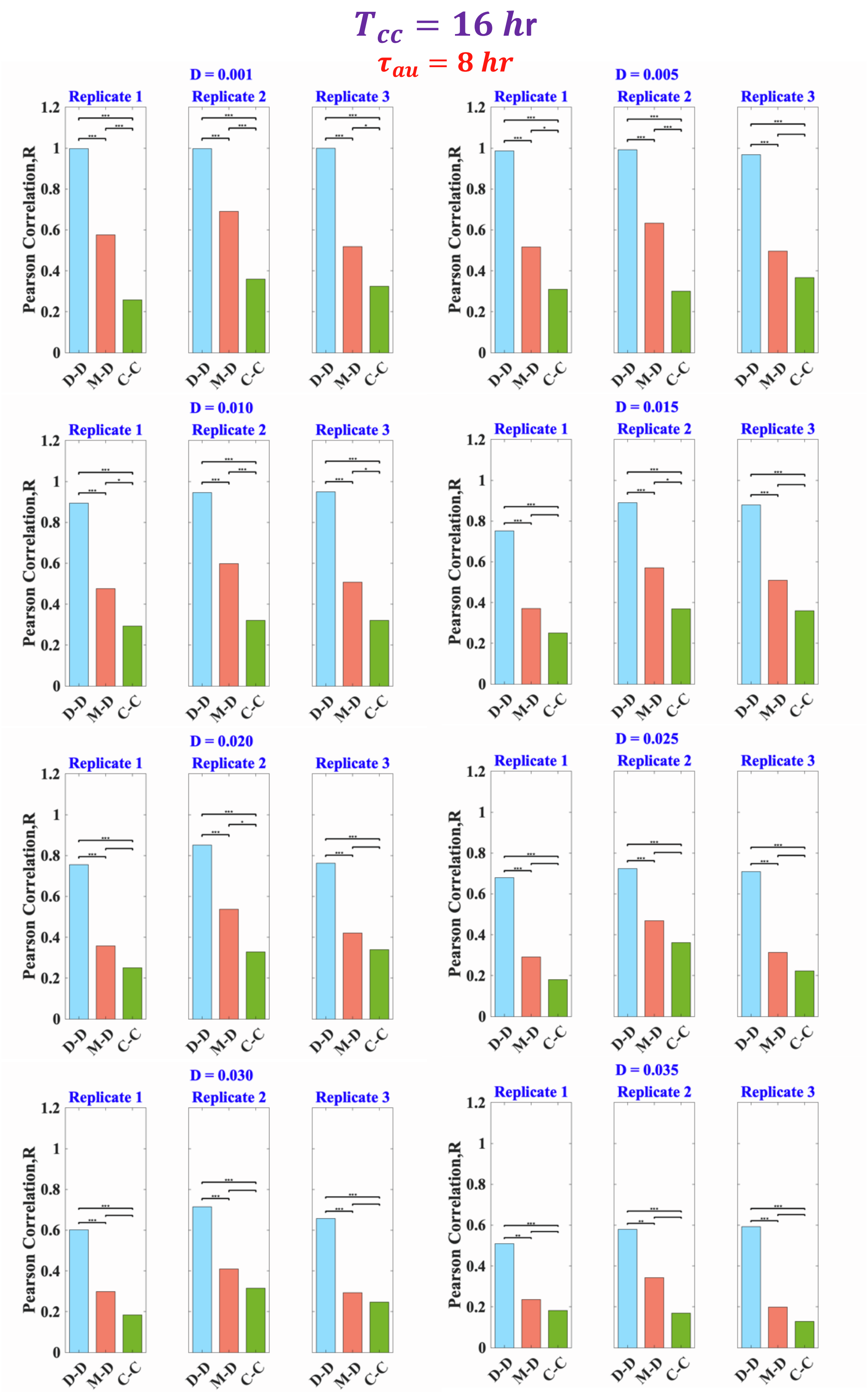

Figure S13 Significance analysis (Related to **Figure 3A(i) & 3B(i)**) of correlations between lineage pairs of individual replicates for τ_{au} value 8hr (***p<0.05**, ****p<0.001, ***p<0.0001**). p indicates the p-value calculated using a Fisher's z transformation of correlation values from lineage pairs. The total cell cycle time is **16 hours** here.

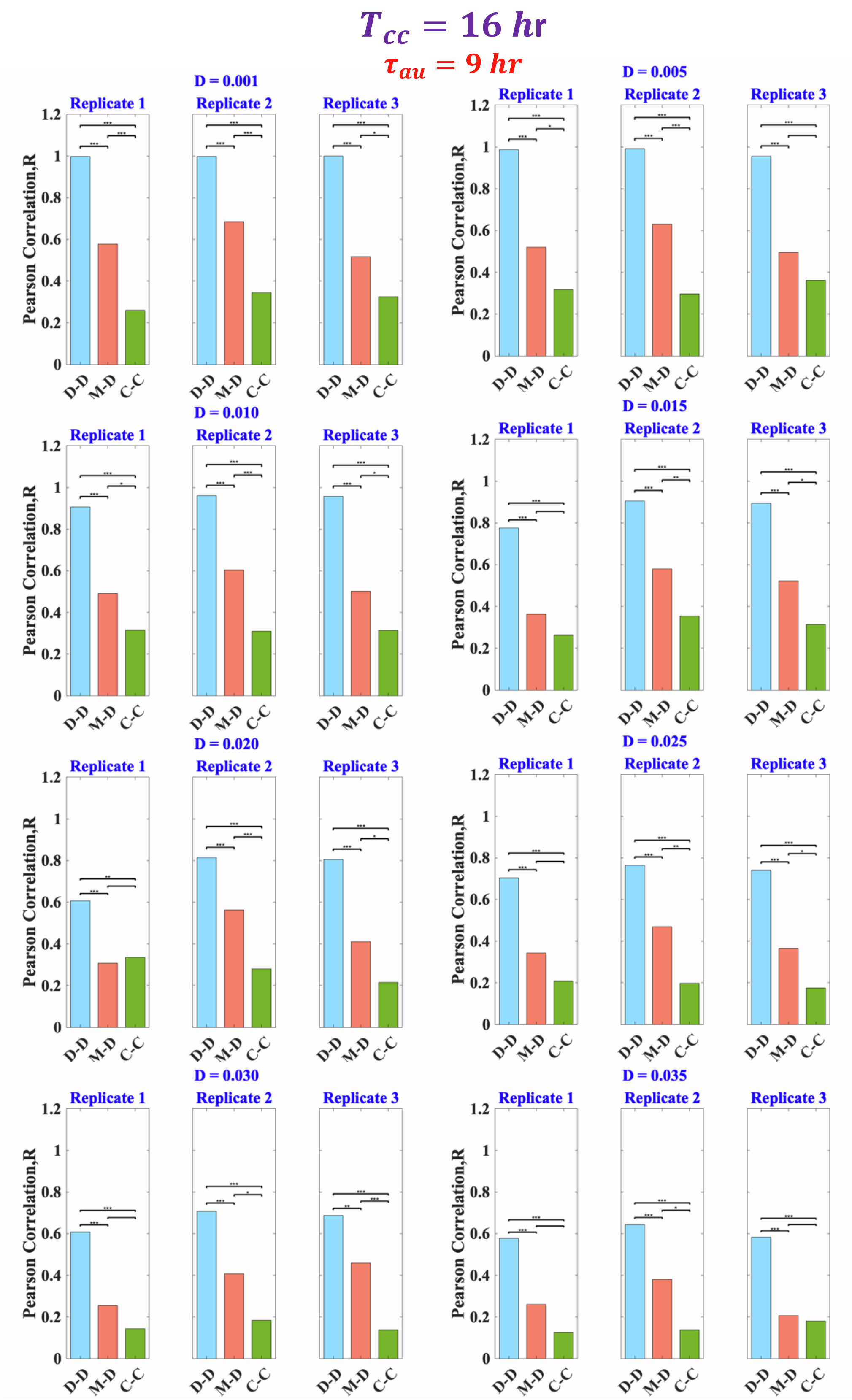

Figure S14 Significance analysis (Related to **Figure 3A(i) & 3B(i)**) of correlations between lineage pairs of individual replicates for τ_{au} value 9hr (***p<0.05**, ****p<0.001, ***p<0.0001**). p indicates the p-value calculated using a Fisher's z transformation of correlation values from lineage pairs. The total cell cycle time is **16 hours** here.

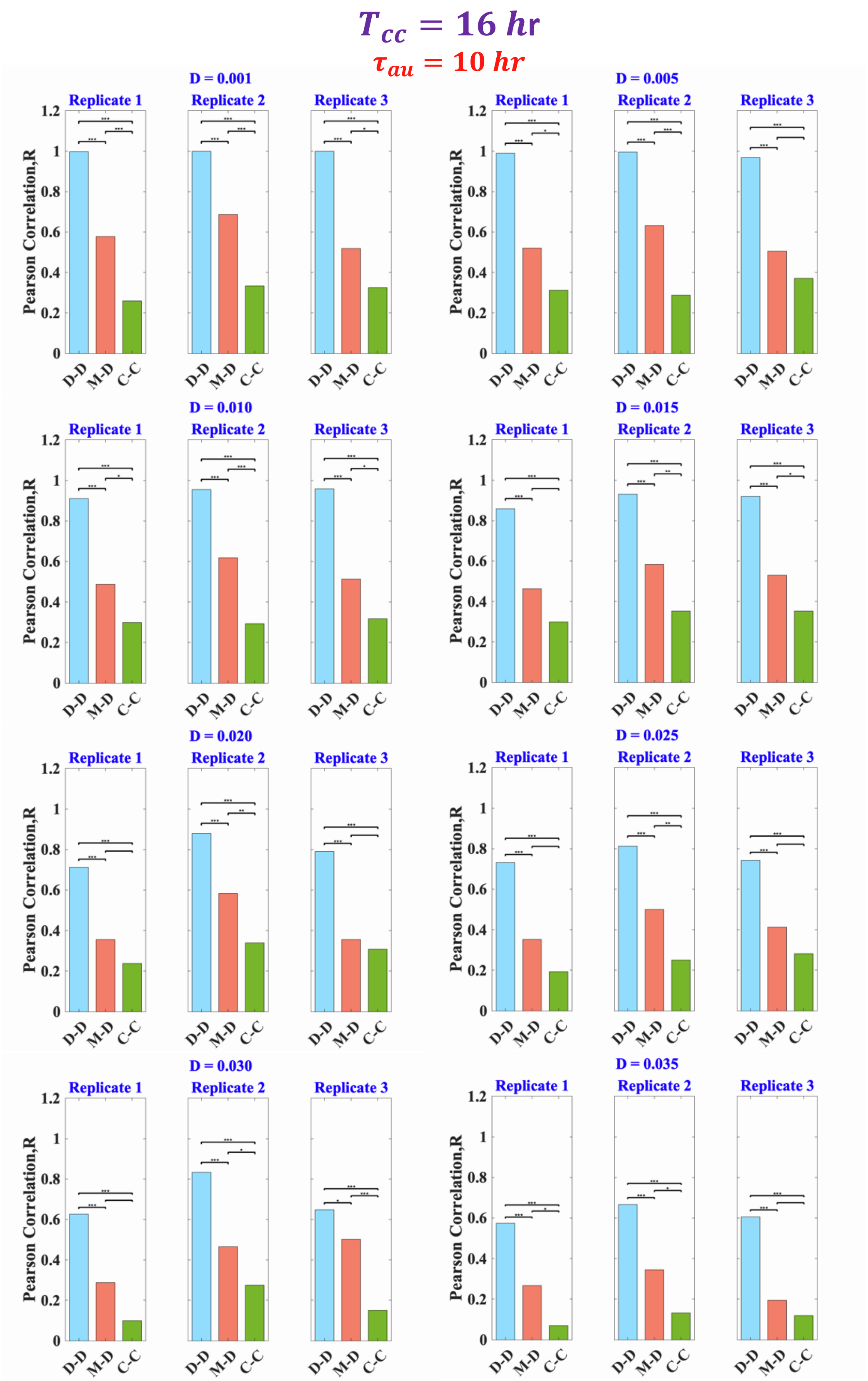

Figure S15 Significance analysis (Related to **Figure 3A(i) & 3B(i)**) of correlations between lineage pairs of individual replicates for τ_{au} value 10hr (***p<0.05**, ****p<0.001, *****p<0.0001**). p indicates the p-value calculated using a Fisher's z transformation of correlation values from lineage pairs. The total cell cycle time is **16 hours** here.

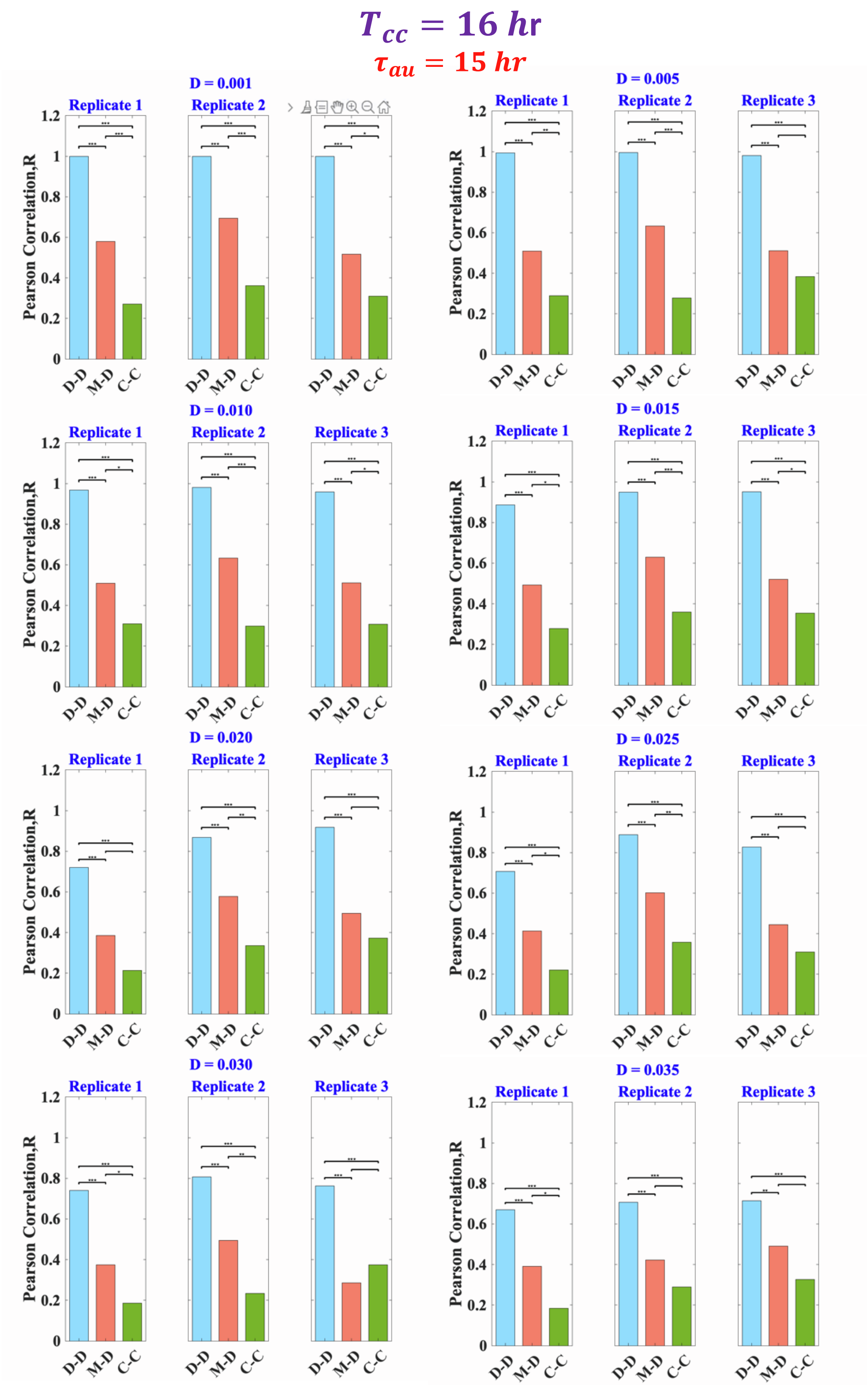

Figure S16 Significance analysis (Related to **Figure 3A(i) & 3B(i)**) of correlations between lineage pairs of individual replicates for τ_{au} value 15hr (***p<0.05**, ****p<0.001, ***p<0.0001**). p indicates the p-value calculated using a Fisher's z transformation of correlation values from lineage pairs. The total cell cycle time is **16 hours** here.

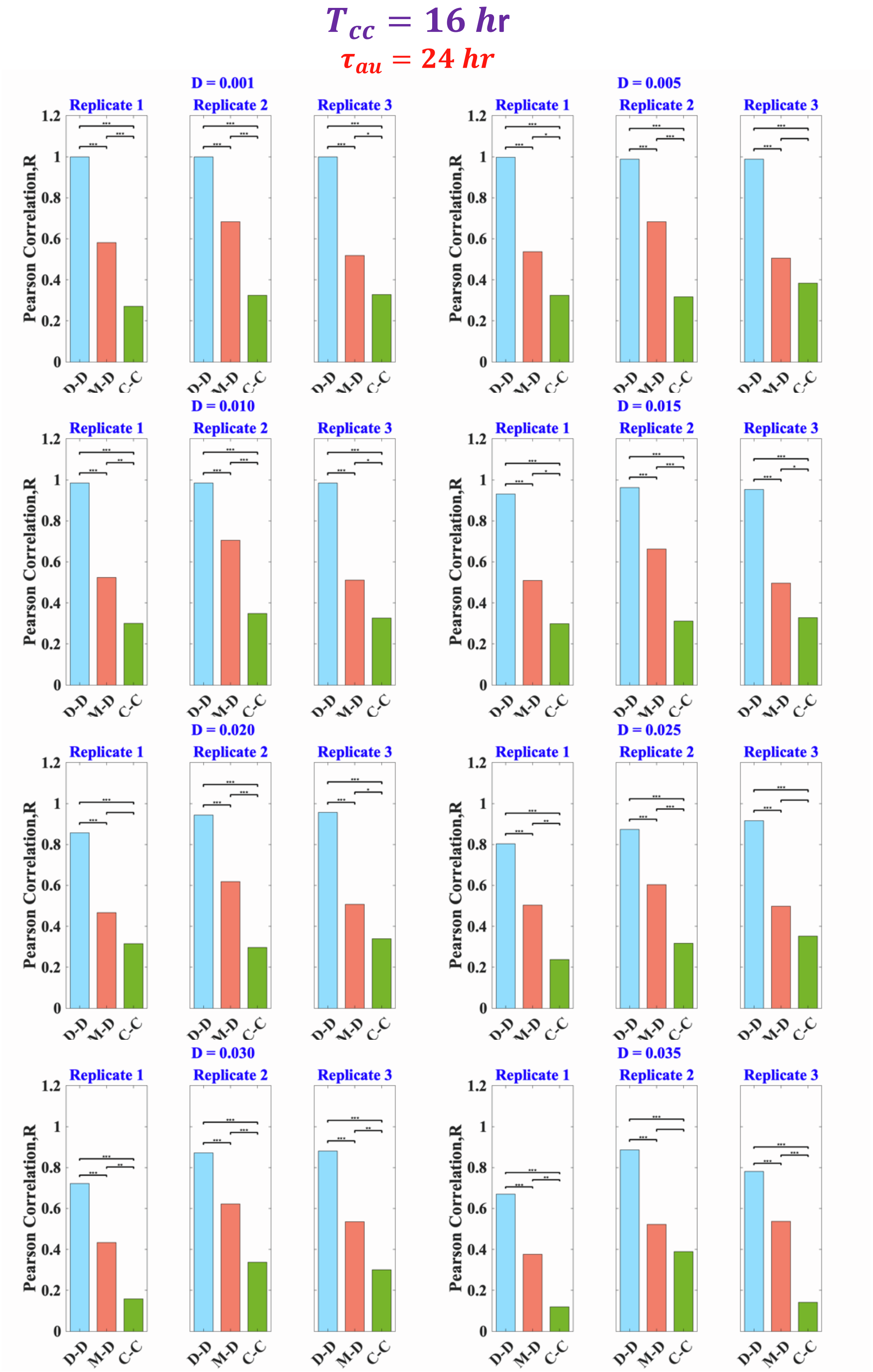

Figure S17 Significance analysis (Related to **Figure 3A(i) & 3B(i)**) of correlations between lineage pairs of individual replicates for τ_{au} value 24hr (***p<0.05**, ****p<0.001, ***p<0.0001**). p indicates the p-value calculated using a Fisher's z transformation of correlation values from lineage pairs. The total cell cycle time is **16 hours** here.

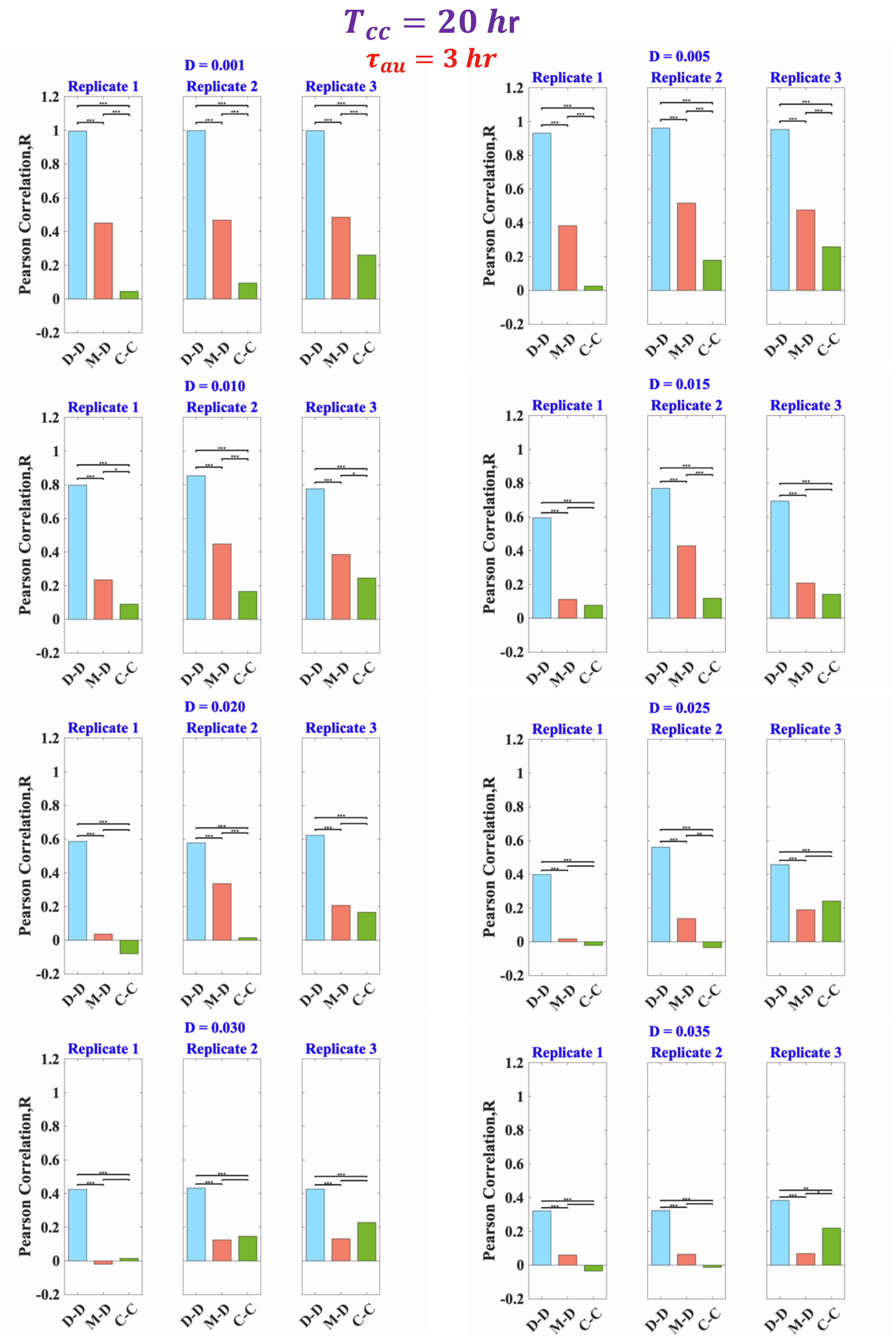

Figure S18 Significance analysis (Related to **Figure 3A(ii) & 3B(ii)**) of correlations between lineage pairs of individual replicates for τ_{au} value 3hr (***p<0.05**, ****p<0.001, *****p<0.0001**). p indicates the p-value calculated using a Fisher's z transformation of correlation values from lineage pairs. The total cell cycle time is **20 hours** here.

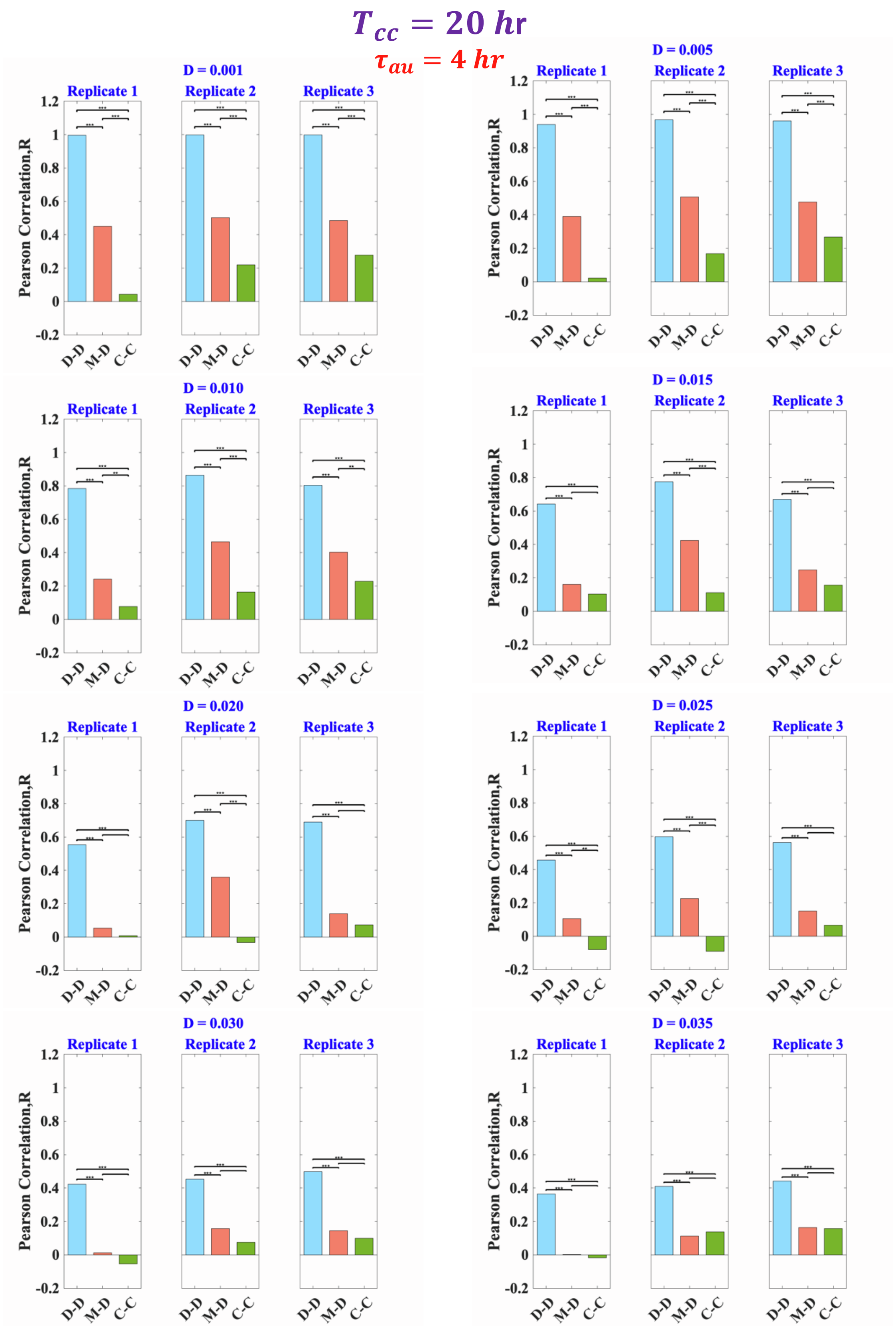

Figure S19 Significance analysis (Related to **Figure 3A(ii) & 3B(ii)**) of correlations between lineage pairs of individual replicates for τ_{au} value 4hr (***p<0.05**, ****p<0.001, ***p<0.0001**). p indicates the p-value calculated using a Fisher's z transformation of correlation values from lineage pairs. The total cell cycle time is **20 hours** here.

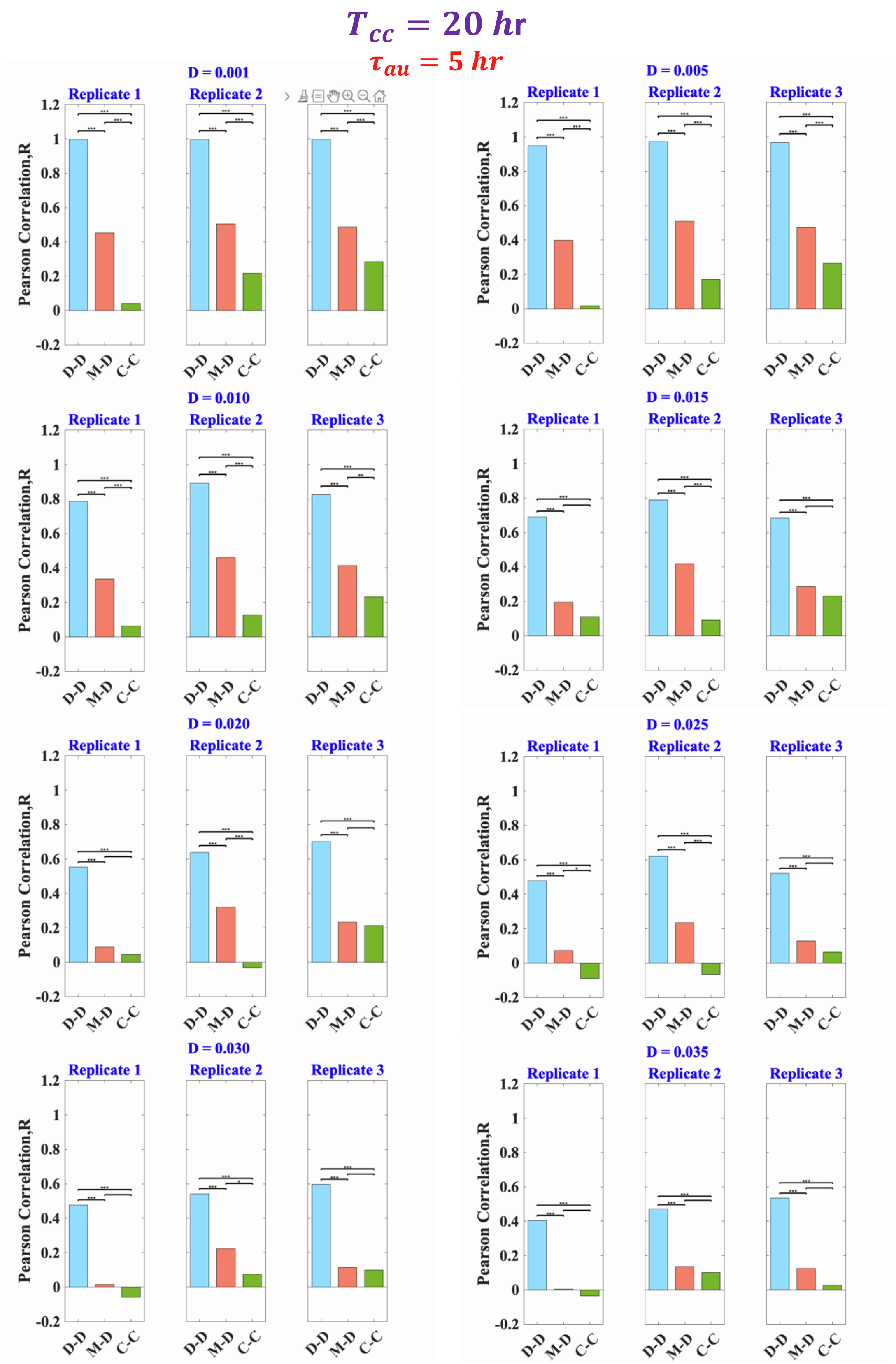

Figure S20 Significance analysis (Related to **Figure 3A(ii) & 3B(ii)**) of correlations between lineage pairs of individual replicates for τ_{au} value 5hr (***p<0.05**, ****p<0.001, *****p<0.0001**). p indicates the p-value calculated using a Fisher's z transformation of correlation values from lineage pairs. The total cell cycle time is **20 hours** here.

$T_{cc} = 20\ hr$

$\tau_{au} = 6\ hr$

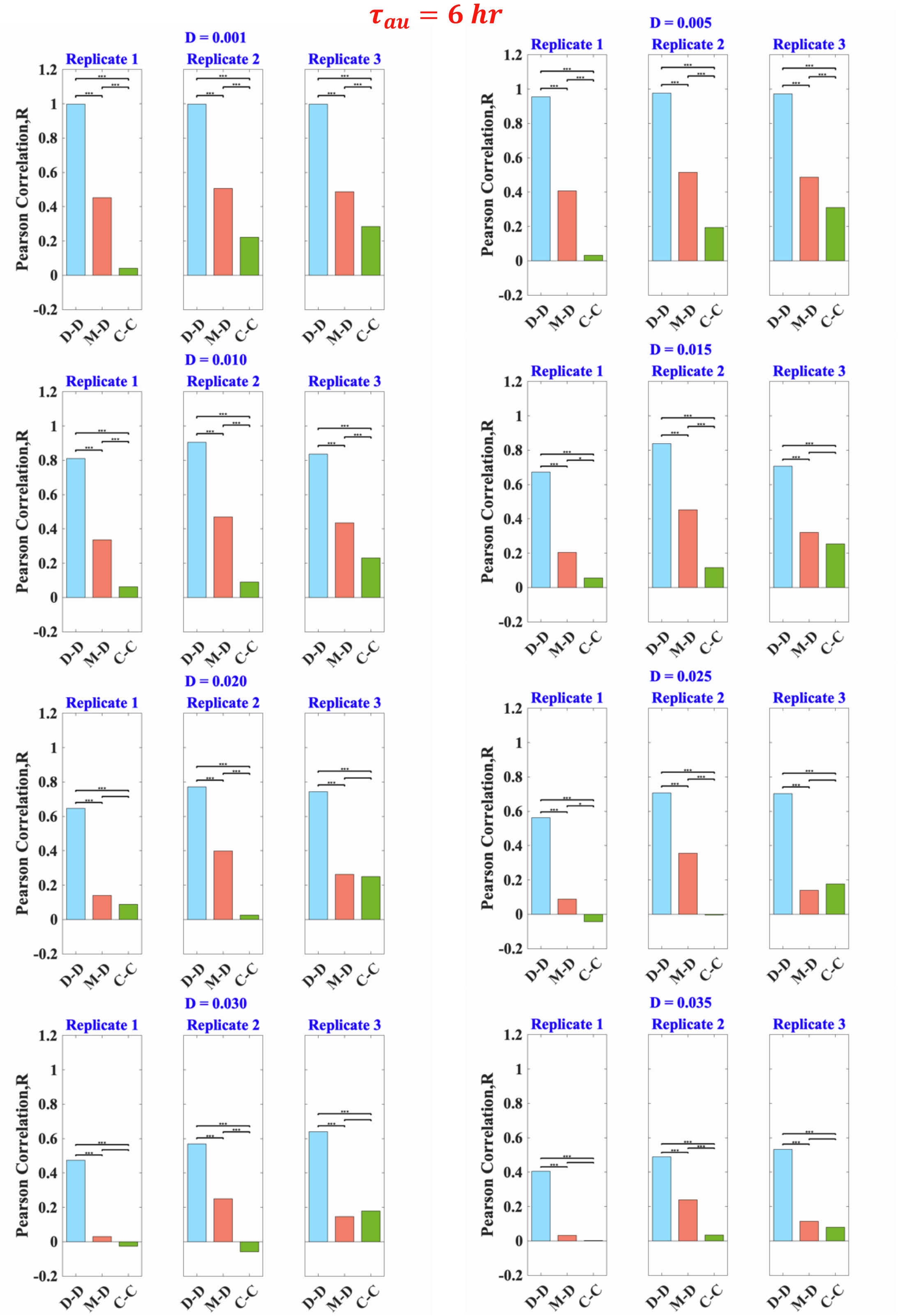

Figure S21 Significance analysis (Related to **Figure 3A(ii) & 3B(ii)**) of correlations between lineage pairs of individual replicates for τ_{au} value 6hr (***p<0.05**, ****p<0.001, *****p<0.0001**). p indicates the p-value calculated using a Fisher's z transformation of correlation values from lineage pairs. The total cell cycle time is **20 hours** here.

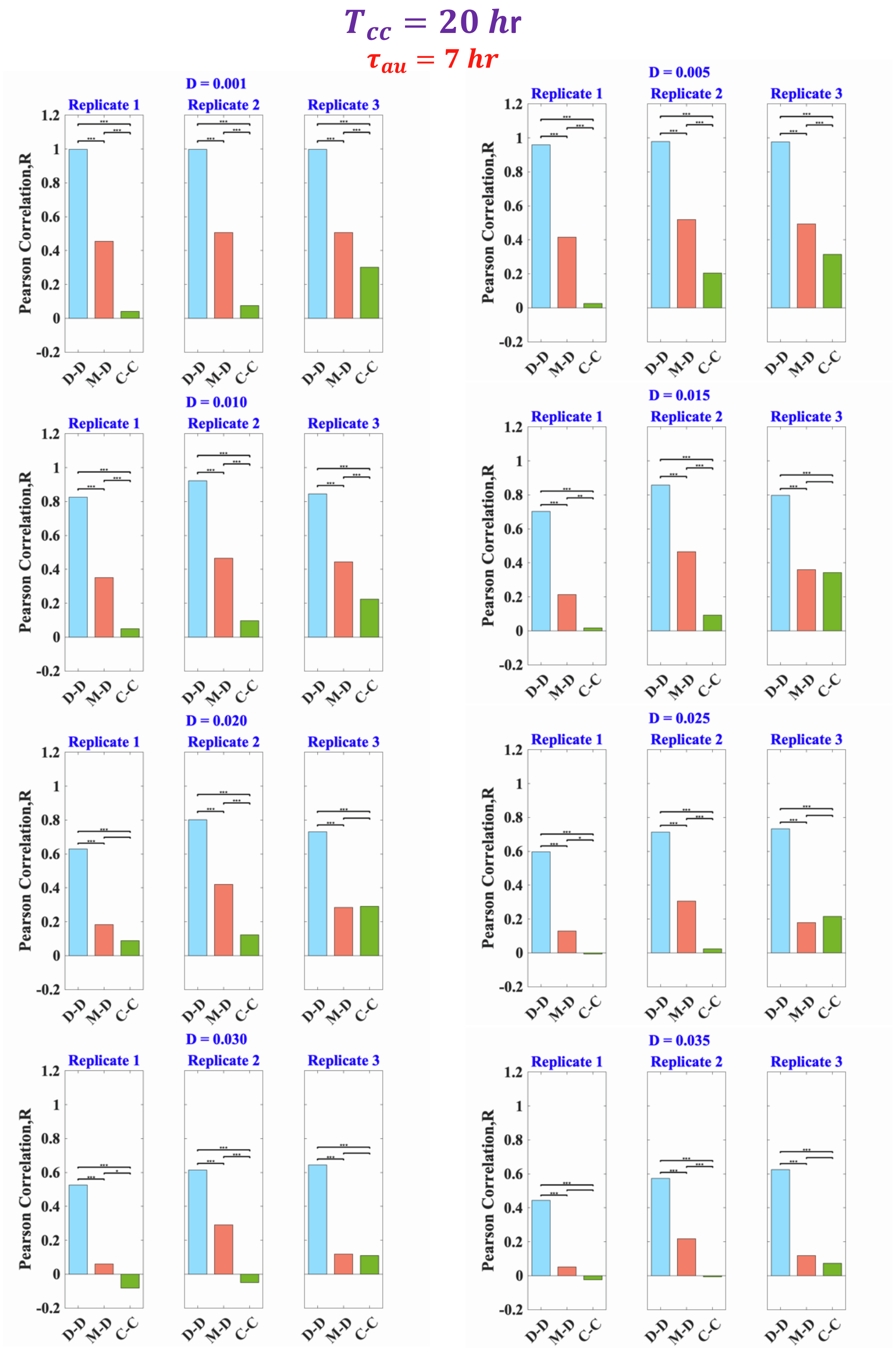

Figure S22 Significance analysis (Related to **Figure 3A(ii) & 3B(ii)**) of correlations between lineage pairs of individual replicates for τ_{au} value 7hr (***p<0.05**, ****p<0.001, ***p<0.0001**). p indicates the p-value calculated using a Fisher's z transformation of correlation values from lineage pairs. The total cell cycle time is **20 hours** here.

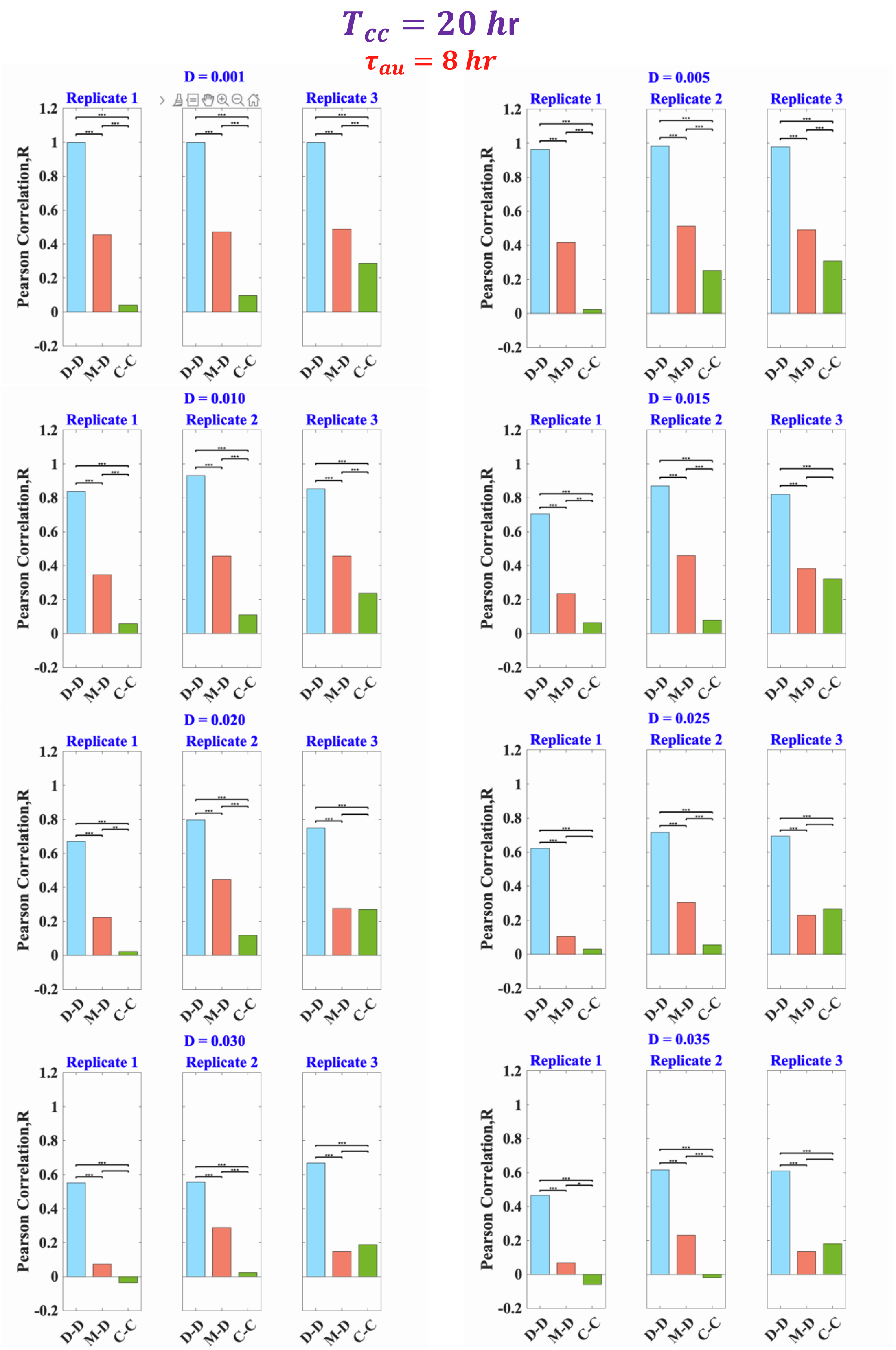

Figure S23 Significance analysis (Related to **Figure 3A(ii) & 3B(ii)**) of correlations between lineage pairs of individual replicates for τ_{au} value 8hr (***p<0.05, **p<0.001, ***p<0.0001**). p indicates the p-value calculated using a Fisher's z transformation of correlation values from lineage pairs. The total cell cycle time is **20 hours** here.

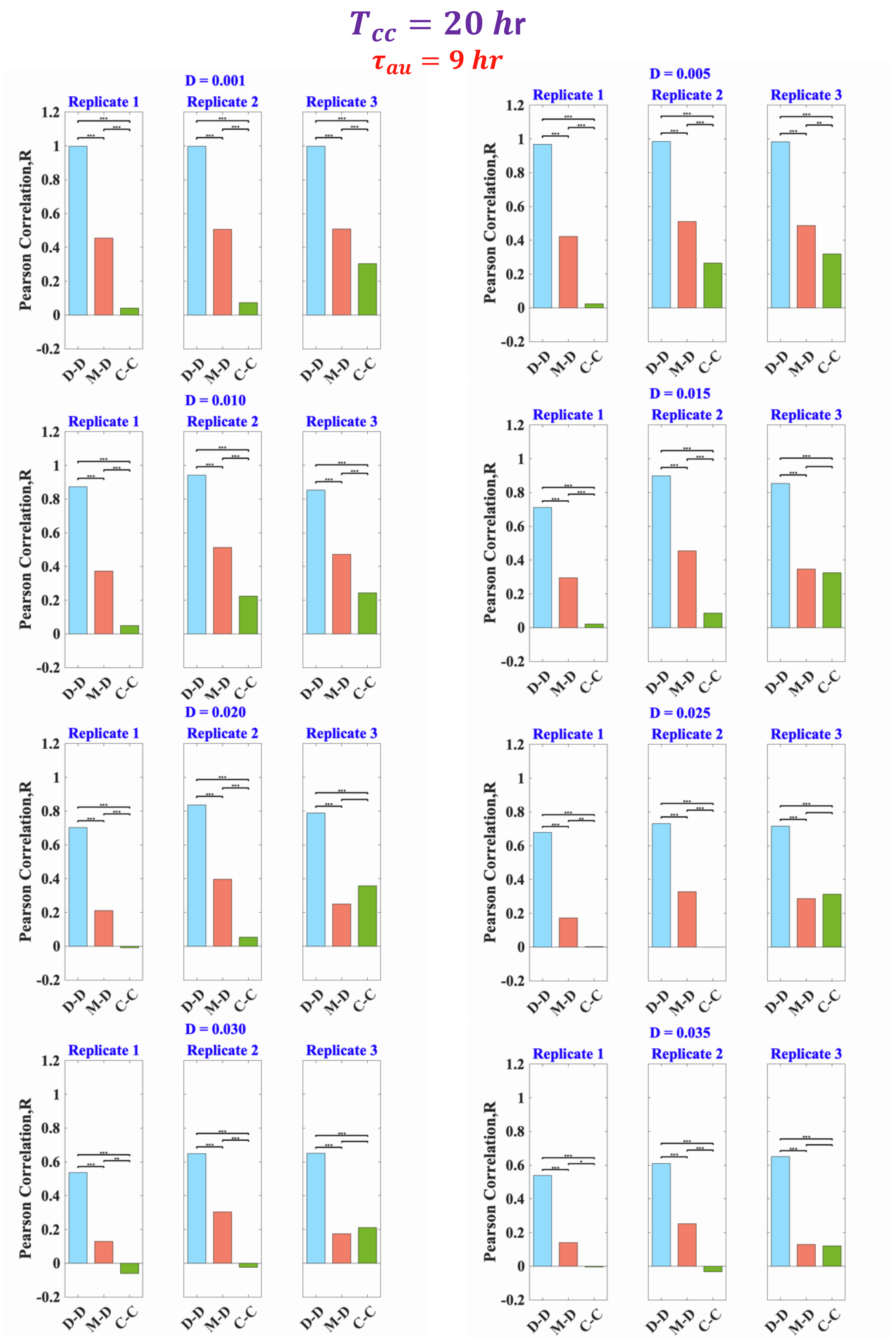

Figure S24 Significance analysis (Related to **Figure 3A(ii) & 3B(ii)**) of correlations between lineage pairs of individual replicates for τ_{au} value 9hr (***p<0.05**, ****p<0.001, ***p<0.0001**). p indicates the p-value calculated using a Fisher's z transformation of correlation values from lineage pairs. The total cell cycle time is **20 hours** here.

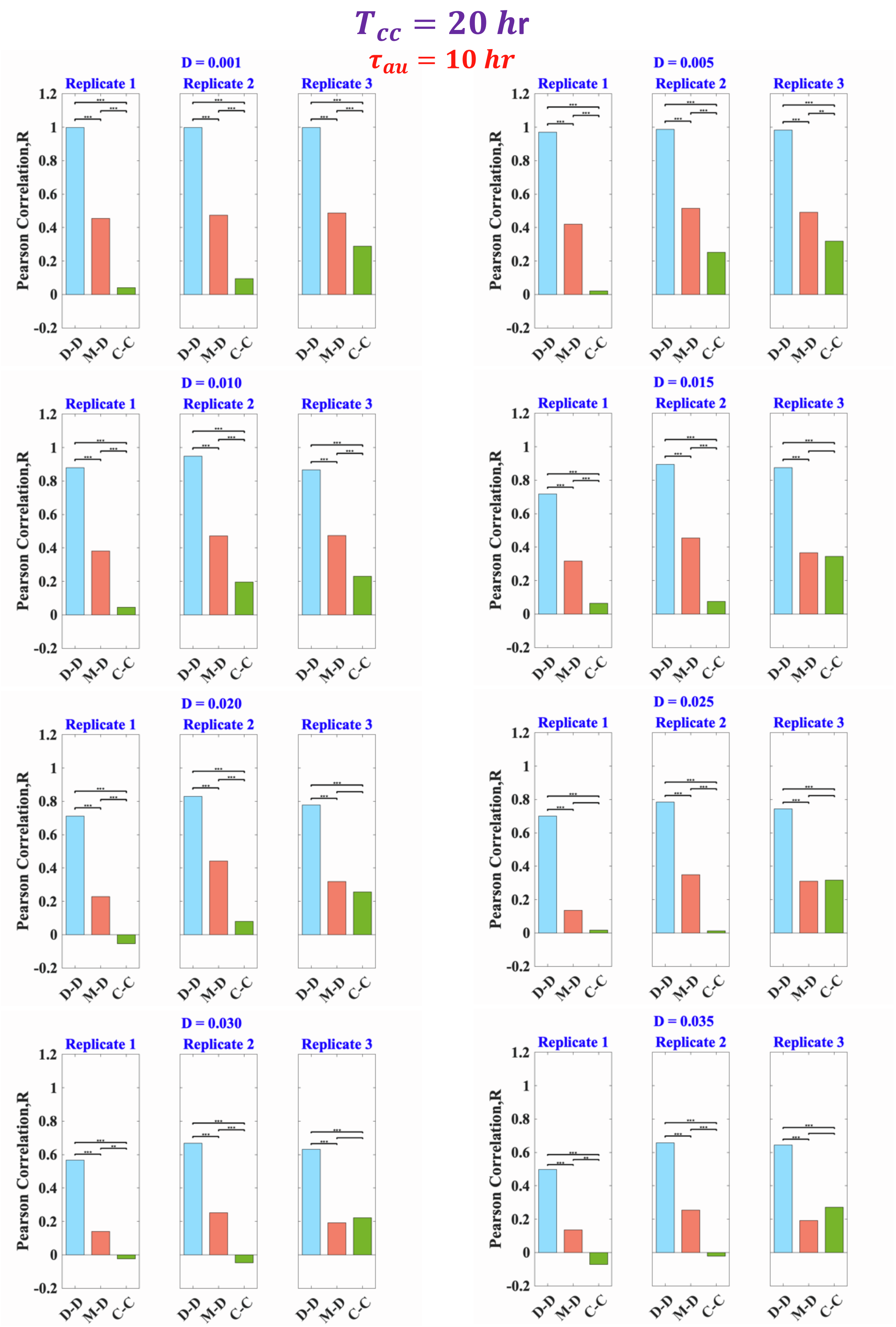

Figure S25 Significance analysis (Related to **Figure 3A(ii) & 3B(ii)**) of correlations between lineage pairs of individual replicates for τ_{au} value 10hr (***p<0.05**, ****p<0.001, *****p<0.0001**). p indicates the p-value calculated using a Fisher's z transformation of correlation values from lineage pairs. The total cell cycle time is **20 hours** here.

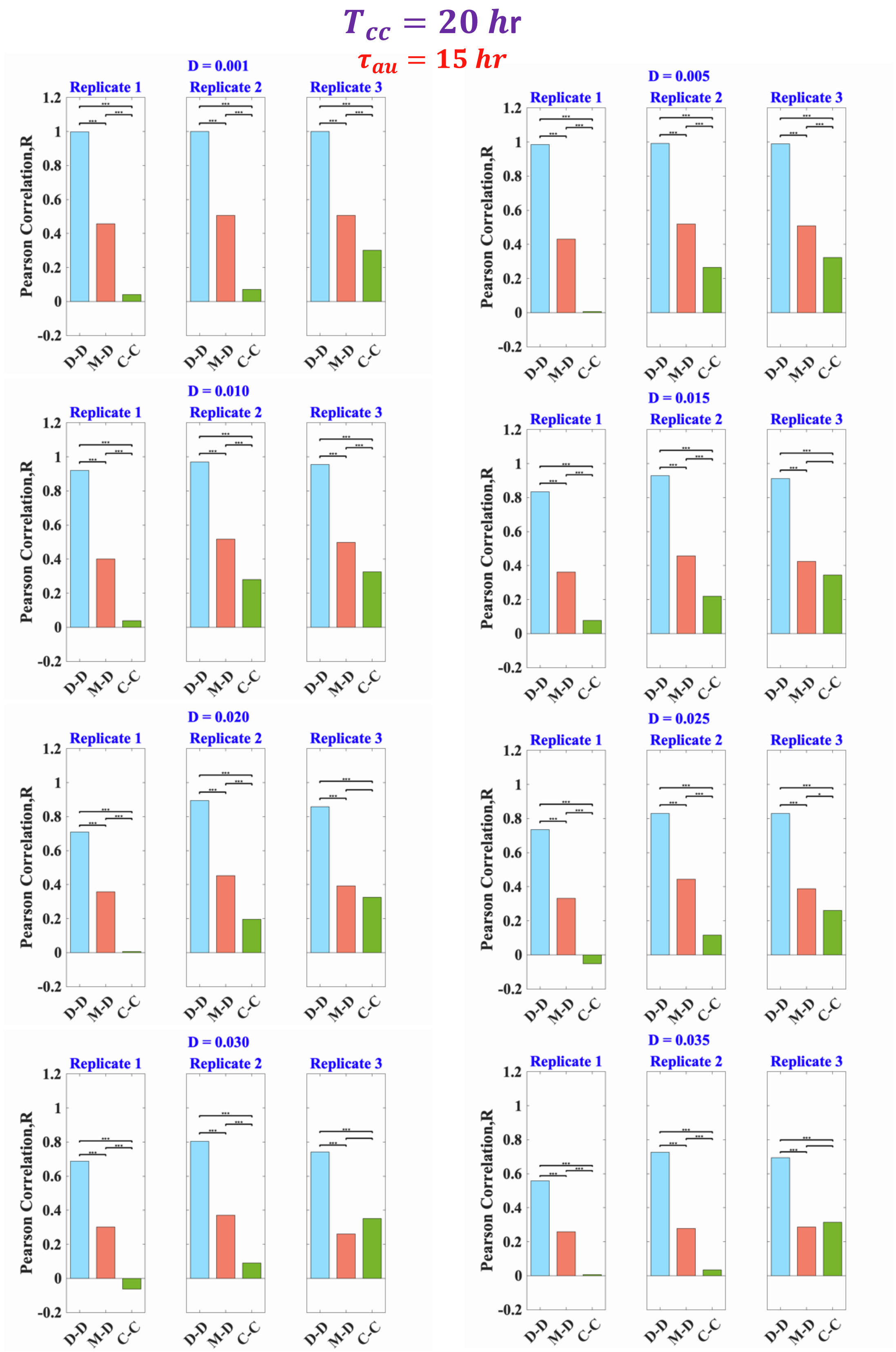

Figure S26 Significance analysis (Related to **Figure 3A(ii) & 3B(ii)**) of correlations between lineage pairs of individual replicates for τ_{au} value 15hr (***p<0.05**, ****p<0.001, *****p<0.0001**). p indicates the p-value calculated using a Fisher's z transformation of correlation values from lineage pairs. The total cell cycle time is **20 hours** here.

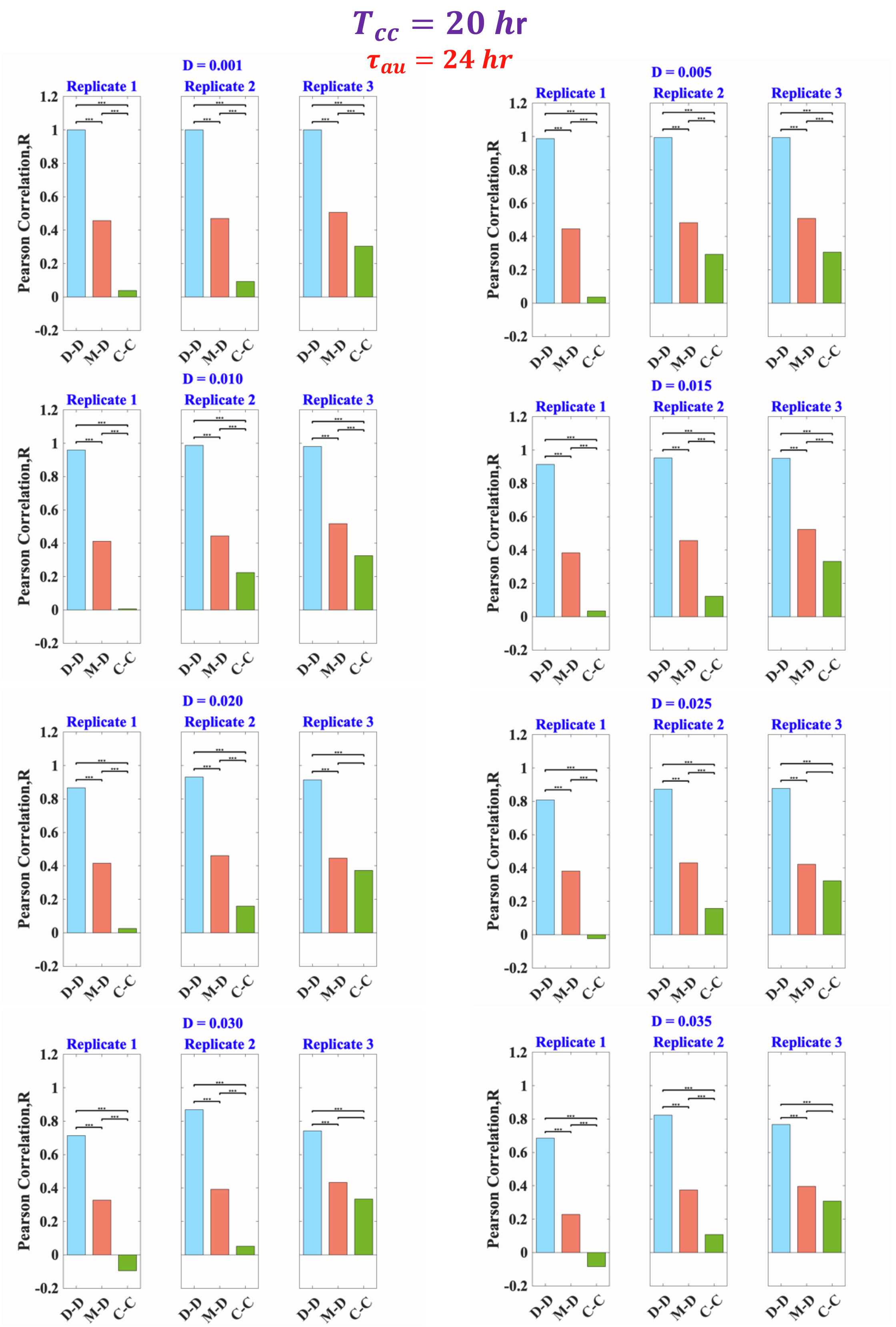

Figure S27 Significance analysis (Related to **Figure 3A(ii) & 3B(ii)**) of correlations between lineage pairs of individual replicates for τ_{au} value 24hr (***p<0.05**, ****p<0.001, *****p<0.0001**). p indicates the p-value calculated using a Fisher's z transformation of correlation values from lineage pairs. The total cell cycle time is **20 hours** here.

$T_{cc} = 24\ hr$

$\tau_{au} = 3\ hr$

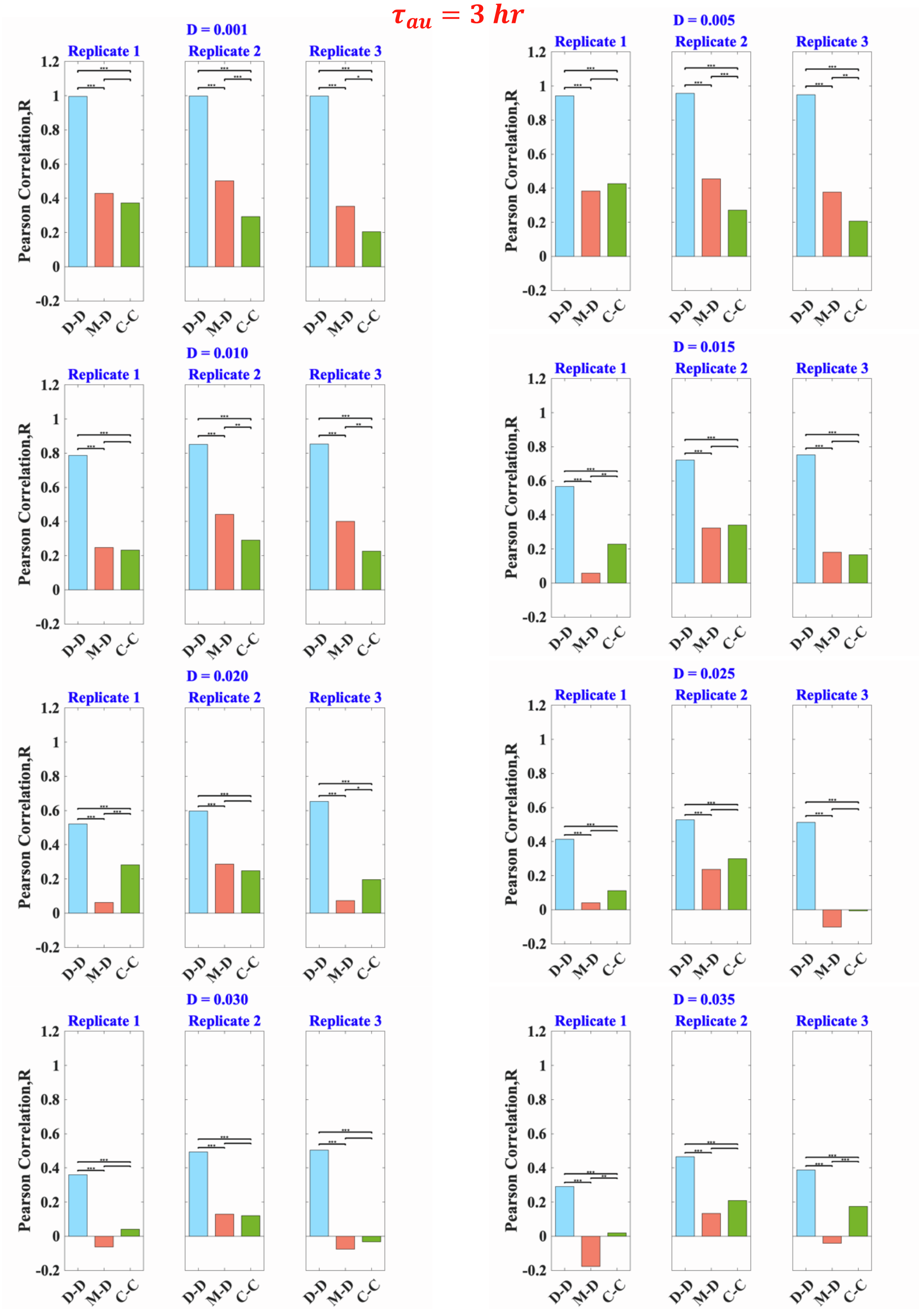

Figure S28 Significance analysis (Related to **Figure 2D(i), Figure 3A(iii) & Figure 3B(iii)**) of correlations between lineage pairs of individual replicates for τ_{au} value 3hr. (***p<0.05**, ****p<0.001, ***p<0.0001**). p indicates the p-value calculated using a Fisher's z transformation of correlation values from lineage pairs. The total cell cycle time is **24 hours** here.

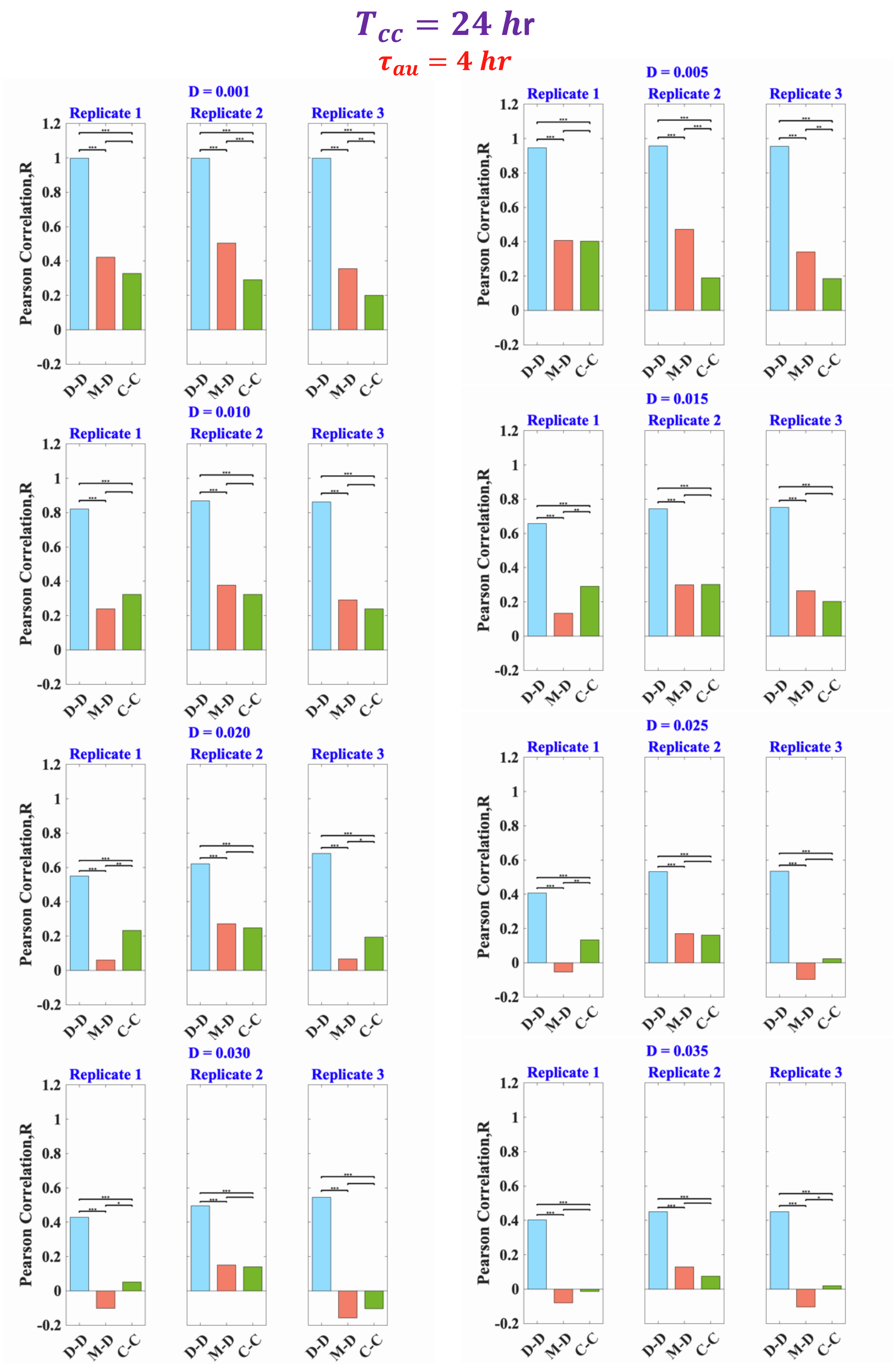

Figure S29 Significance analysis (Related to **Figure 2D(i), Figure 3A(iii) & Figure 3B(iii)**) of correlations between lineage pairs of individual replicates for τ_{au} value 4hr. (***p<0.05**, ****p<0.001, *****p<0.0001**). p indicates the p-value calculated using a Fisher's z transformation of correlation values from lineage pairs. The total cell cycle time is **24 hours** here.

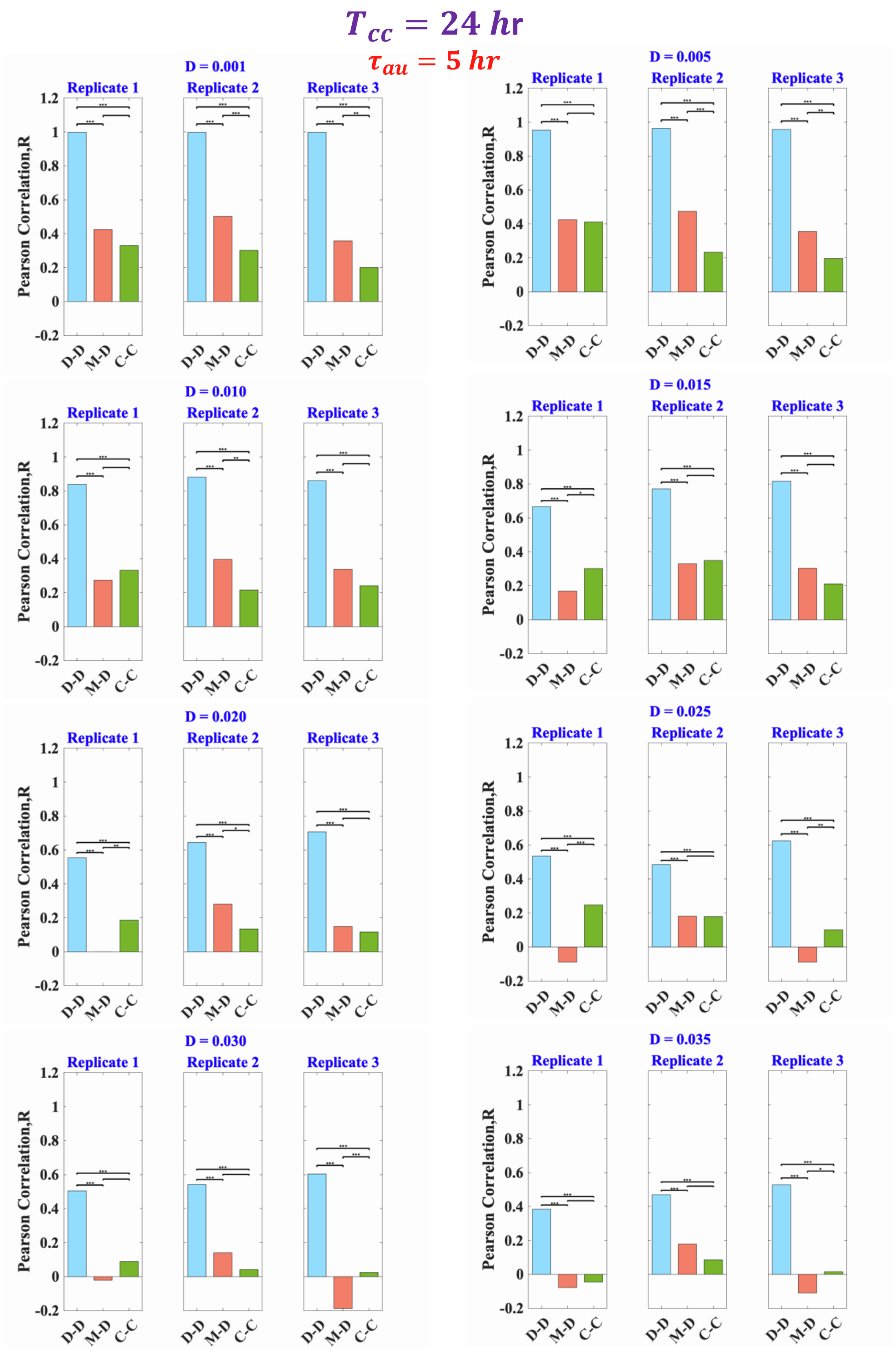

Figure S30 Significance analysis (Related to **Figure 2D(i), Figure 3A(iii) & Figure 3B(iii)**) of correlations between lineage pairs of individual replicates for τ_{au} value 5hr. (***p<0.05**, ****p<0.001, ***p<0.0001**). p indicates the p-value calculated using a Fisher's z transformation of correlation values from lineage pairs. The total cell cycle time is **24 hours** here.

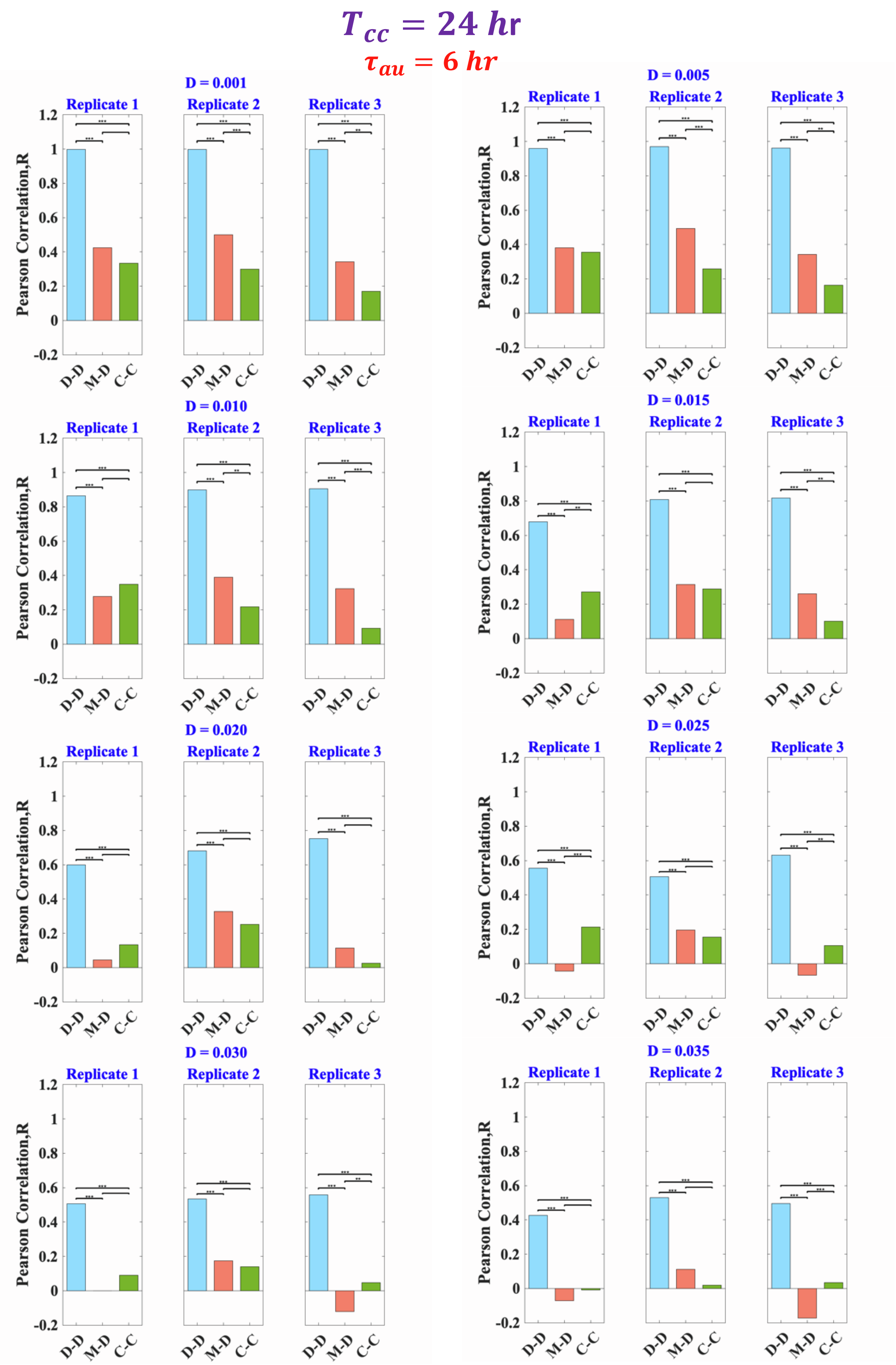

Figure S31 Significance analysis (Related to **Figure 2D(i), Figure 3A(iii) & Figure 3B(iii)**) of correlations between lineage pairs of individual replicates for τ_{au} value 6hr. (***p<0.05**, ****p<0.001, ***p<0.0001**). p indicates the p-value calculated using a Fisher's z transformation of correlation values from lineage pairs. The total cell cycle time is **24 hours** here.

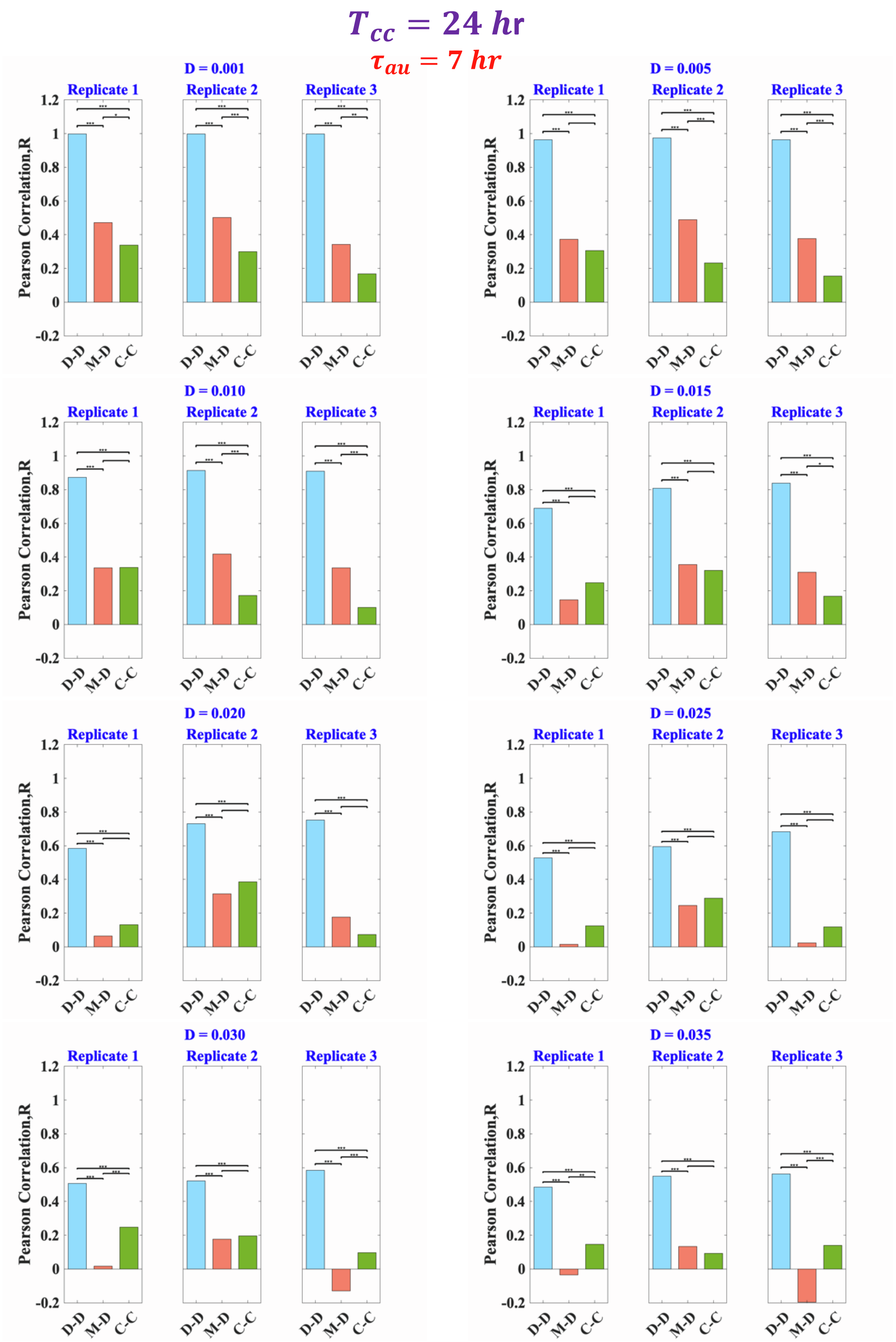

Figure S32 Significance analysis (Related to **Figure 2D(i), Figure 3A(iii) & Figure 3B(iii)**) of correlations between lineage pairs of individual replicates for τ_{au} value 7hr. (***p<0.05**, ****p<0.001, ***p<0.0001**). p indicates the p-value calculated using a Fisher's z transformation of correlation values from lineage pairs. The total cell cycle time is **24 hours** here.

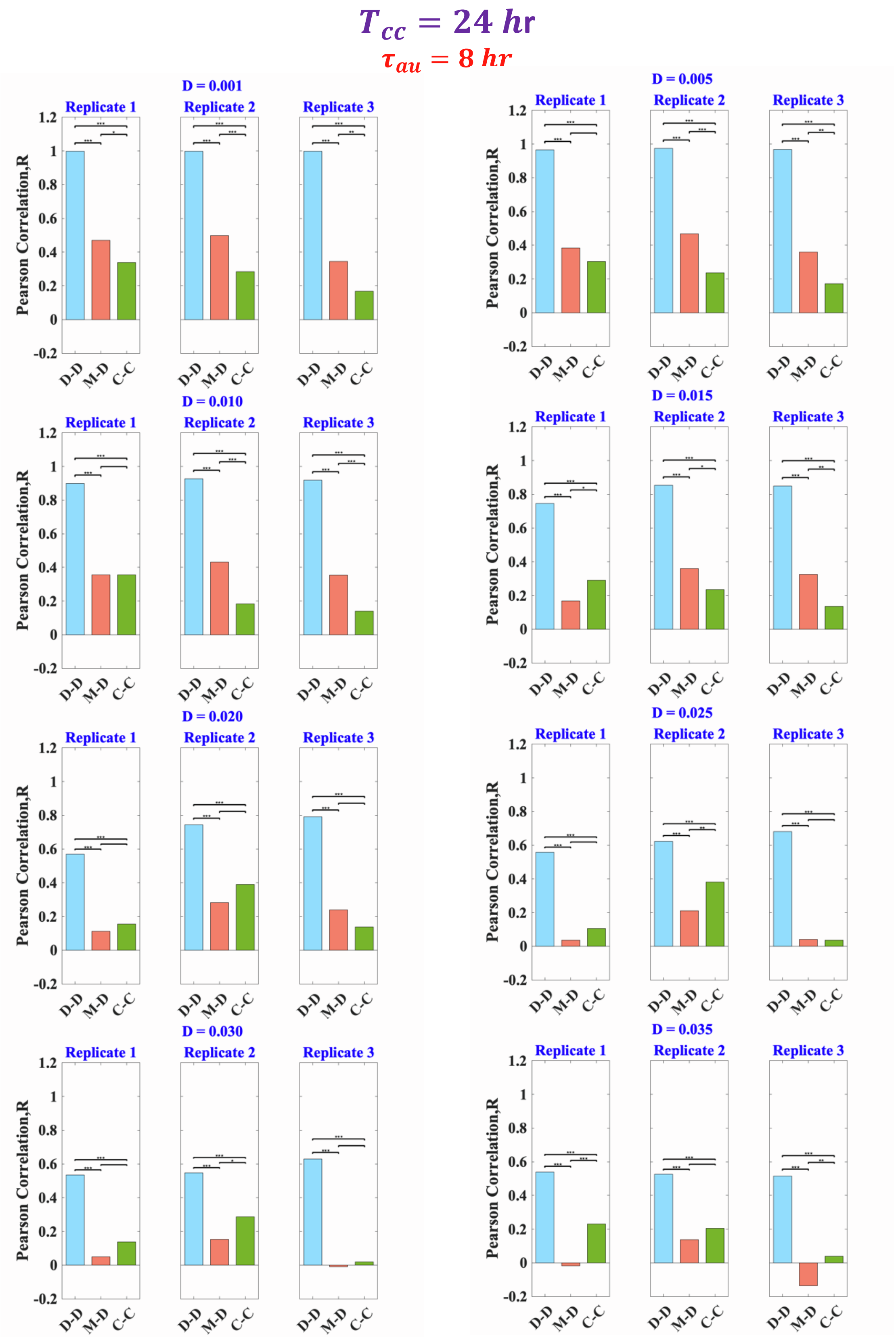

Figure S33 Significance analysis (Related to **Figure 2D(i), Figure 3A(iii) & Figure 3B(iii)**) of correlations between lineage pairs of individual replicates for τ_{au} value 8hr. (***p<0.05**, ****p<0.001, ***p<0.0001**). p indicates the p-value calculated using a Fisher's z transformation of correlation values from lineage pairs. The total cell cycle time is **24 hours** here.

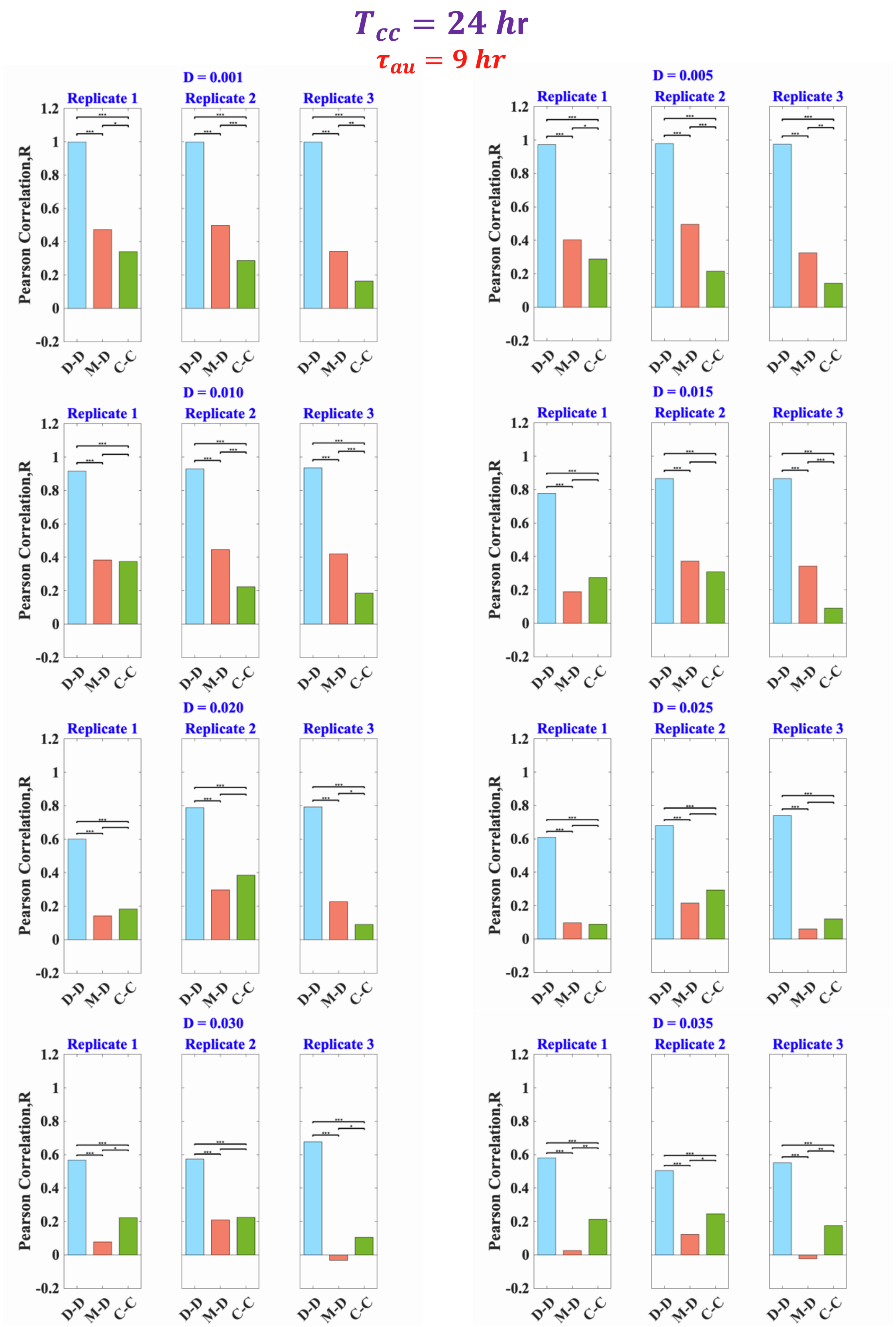

Figure S34 Significance analysis (Related to **Figure 2D(i), Figure 3A(iii) & Figure 3B(iii)**) of correlations between lineage pairs of individual replicates for τ_{au} value 9hr. (***p<0.05**, ****p<0.001, *****p<0.0001**). p indicates the p-value calculated using a Fisher's z transformation of correlation values from lineage pairs. The total cell cycle time is **24 hours** here.

$$T_{cc} = 24\ hr$$
$$\tau_{au} = 10\ hr$$

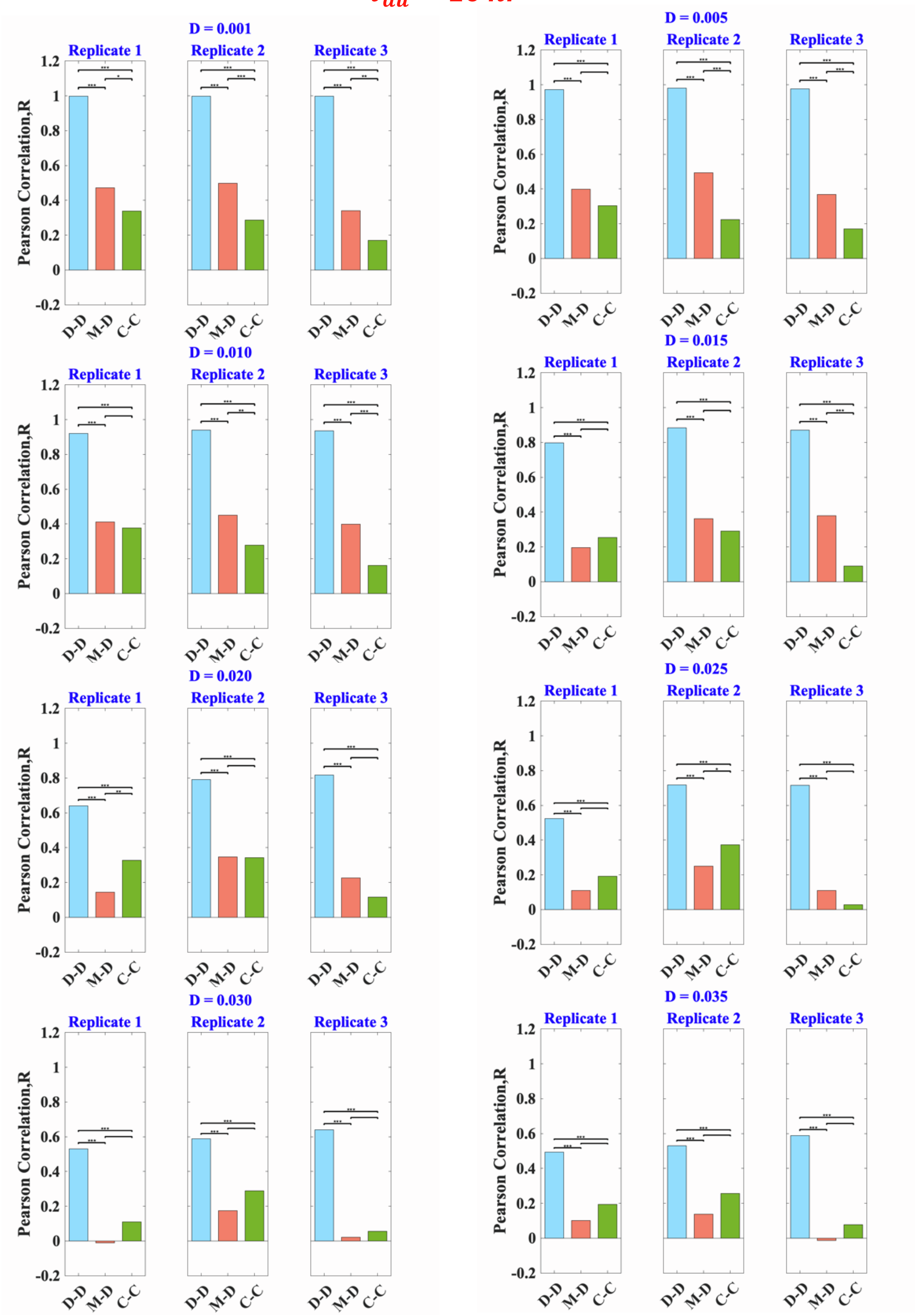

Figure S35 Significance analysis (Related to **Figure 2D(i), Figure 3A(iii) & Figure 3B(iii)**) of correlations between lineage pairs of individual replicates for τ_{au} value 10hr. (***p<0.05**, ****p<0.001, ***p<0.0001**). p indicates the p-value calculated using a Fisher's z transformation of correlation values from lineage pairs. The total cell cycle time is **24 hours** here.

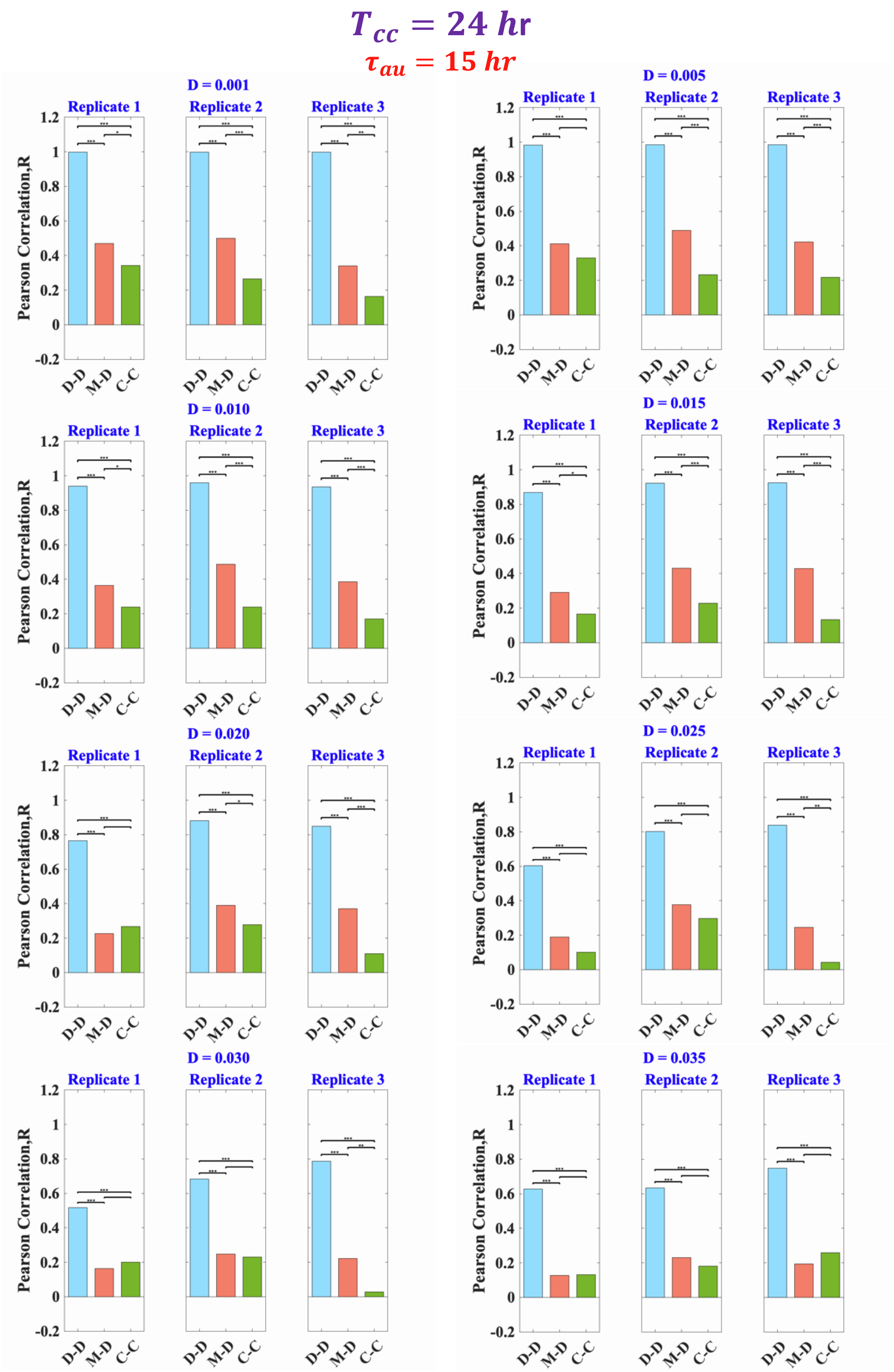

Figure S36 Significance analysis (Related to **Figure 2D(i), Figure 3A(iii) & Figure 3B(iii)**) of correlations between lineage pairs of individual replicates for τ_{au} value 15hr. (***p<0.05**, ****p<0.001, *****p<0.0001**). p indicates the p-value calculated using a Fisher's z transformation of correlation values from lineage pairs. The total cell cycle time is **24 hours** here.

$$T_{cc} = 24\ hr$$
$$\tau_{au} = 24\ hr$$

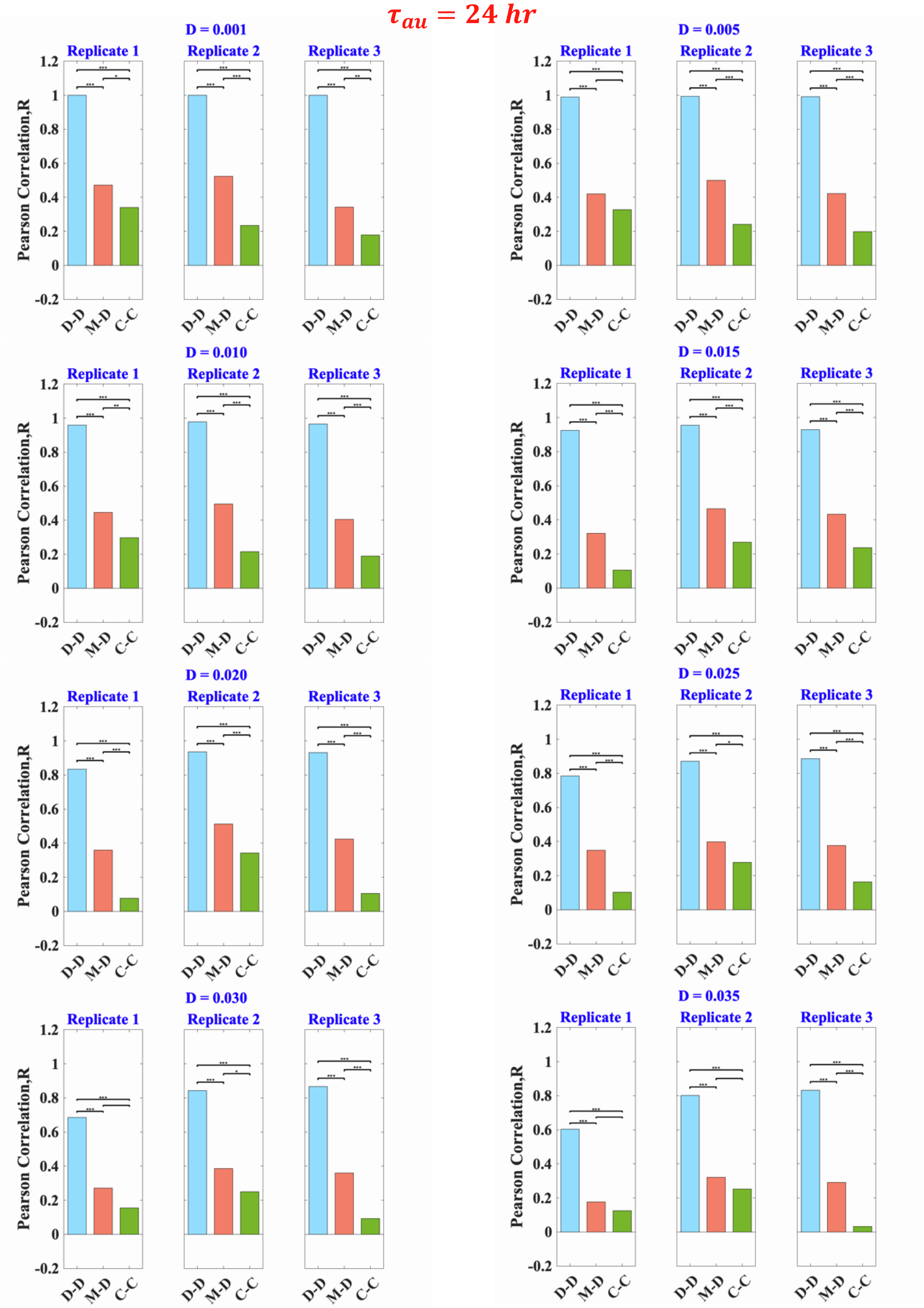

Figure S37 Significance analysis (Related to **Figure 2D(i), Figure 3A(iii) & Figure 3B(iii)**) of correlations between lineage pairs of individual replicates for τ_{au} value 24hr. (***p<0.05**, ****p<0.001, ***p<0.0001**). p indicates the p-value calculated using a Fisher's z transformation of correlation values from lineage pairs. The total cell cycle time is **24 hours** here.

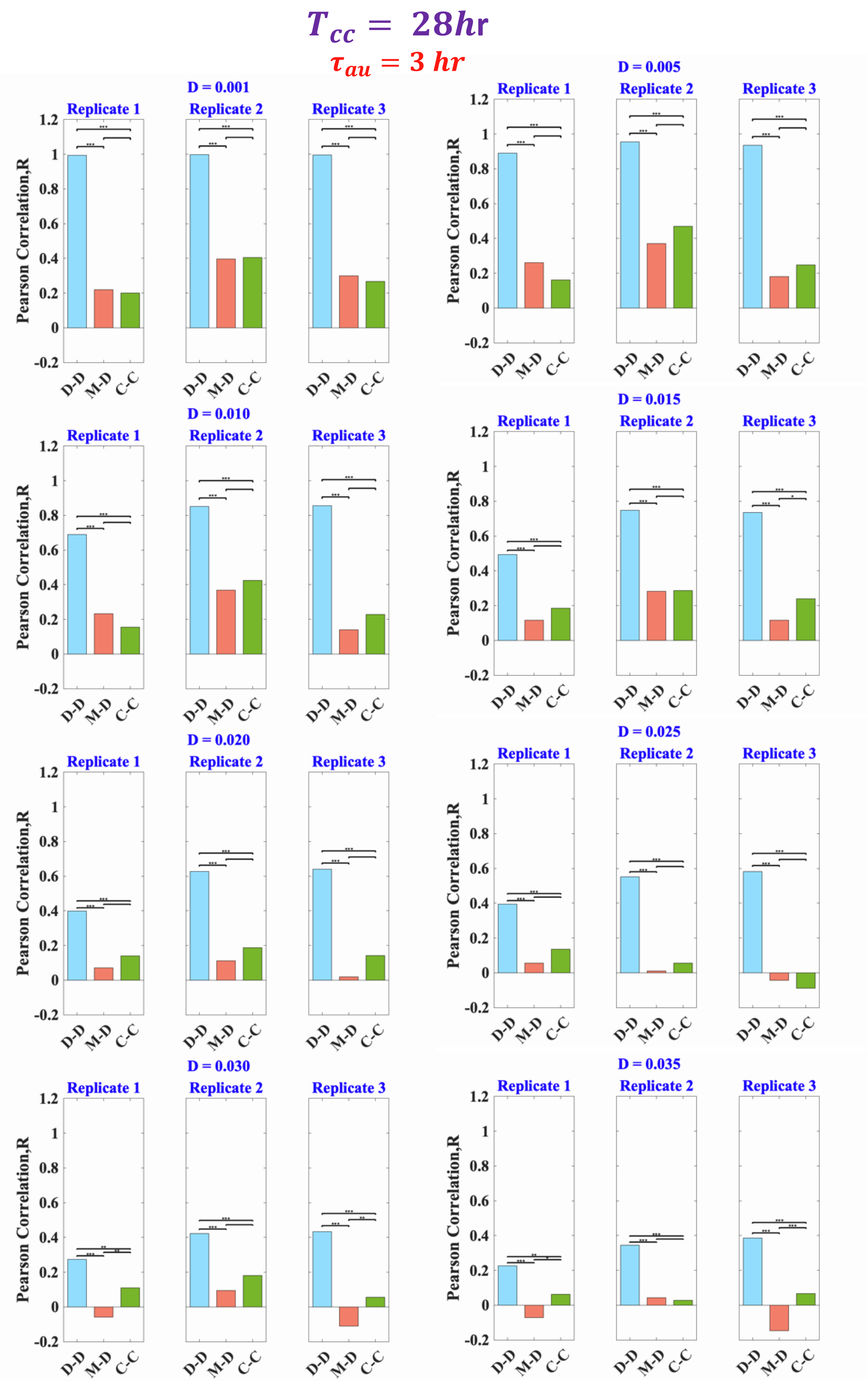

Figure S38 Significance analysis (Related to **Figure 3A(iv) & 3B(iv)**) of correlations between lineage pairs of individual replicates for τ_{au} value 3 hr. (***p<0.05**, ****p<0.001, ***p<0.0001**). p indicates the p-value calculated using a Fisher's z transformation of correlation values from lineage pairs. The total cell cycle time is **28 hours** here.

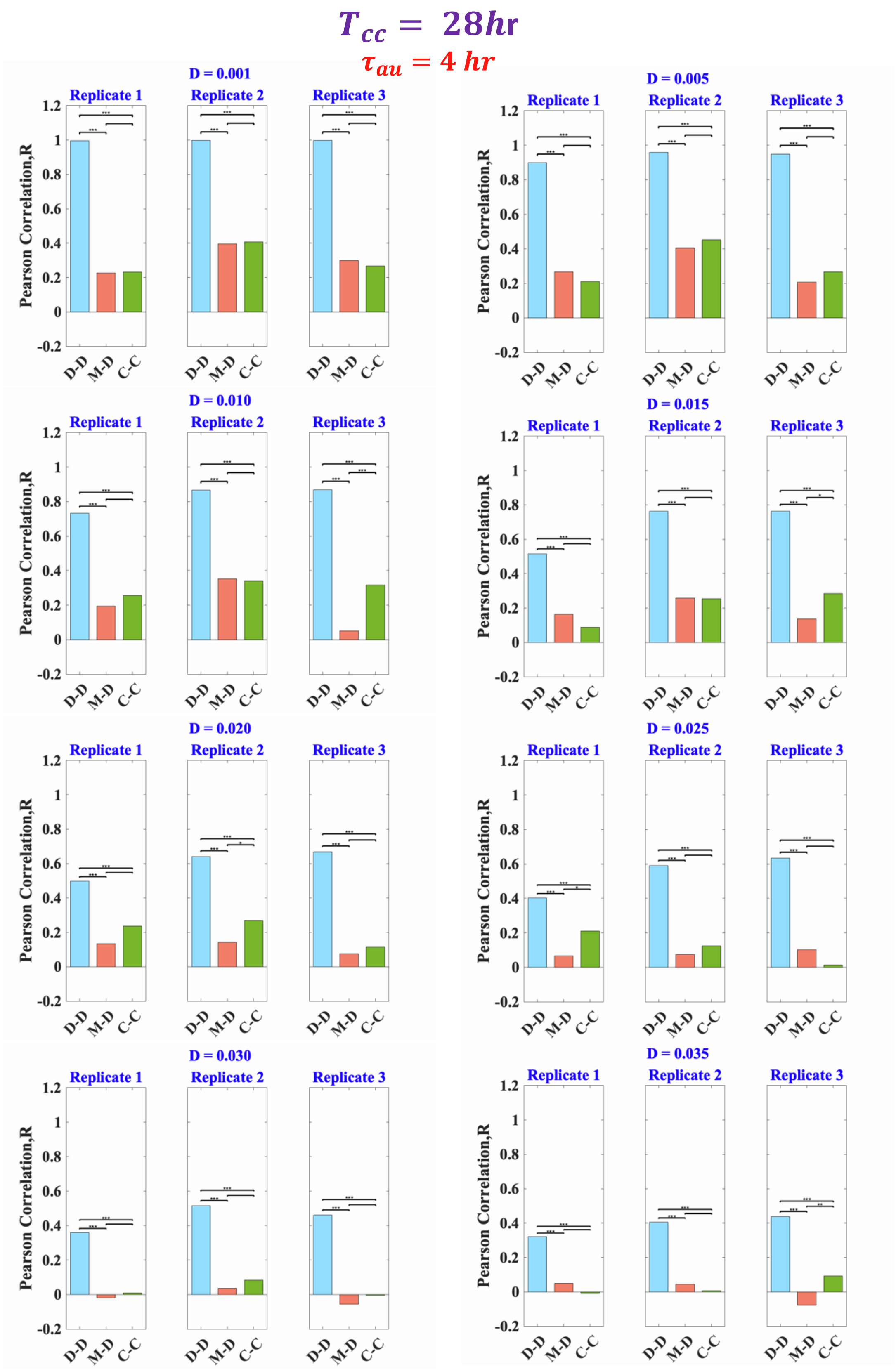

Figure S39 Significance analysis (Related to **Figure 3A(iv) & 3B(iv)**) of correlations between lineage pairs of individual replicates for τ_{au} value 4hr. (***p<0.05**, ****p<0.001, ***p<0.0001**). p indicates the p-value calculated using a Fisher's z transformation of correlation values from lineage pairs. The total cell cycle time is **28 hours** here.

$T_{cc} = 28hr$

$\tau_{au} = 5\ hr$

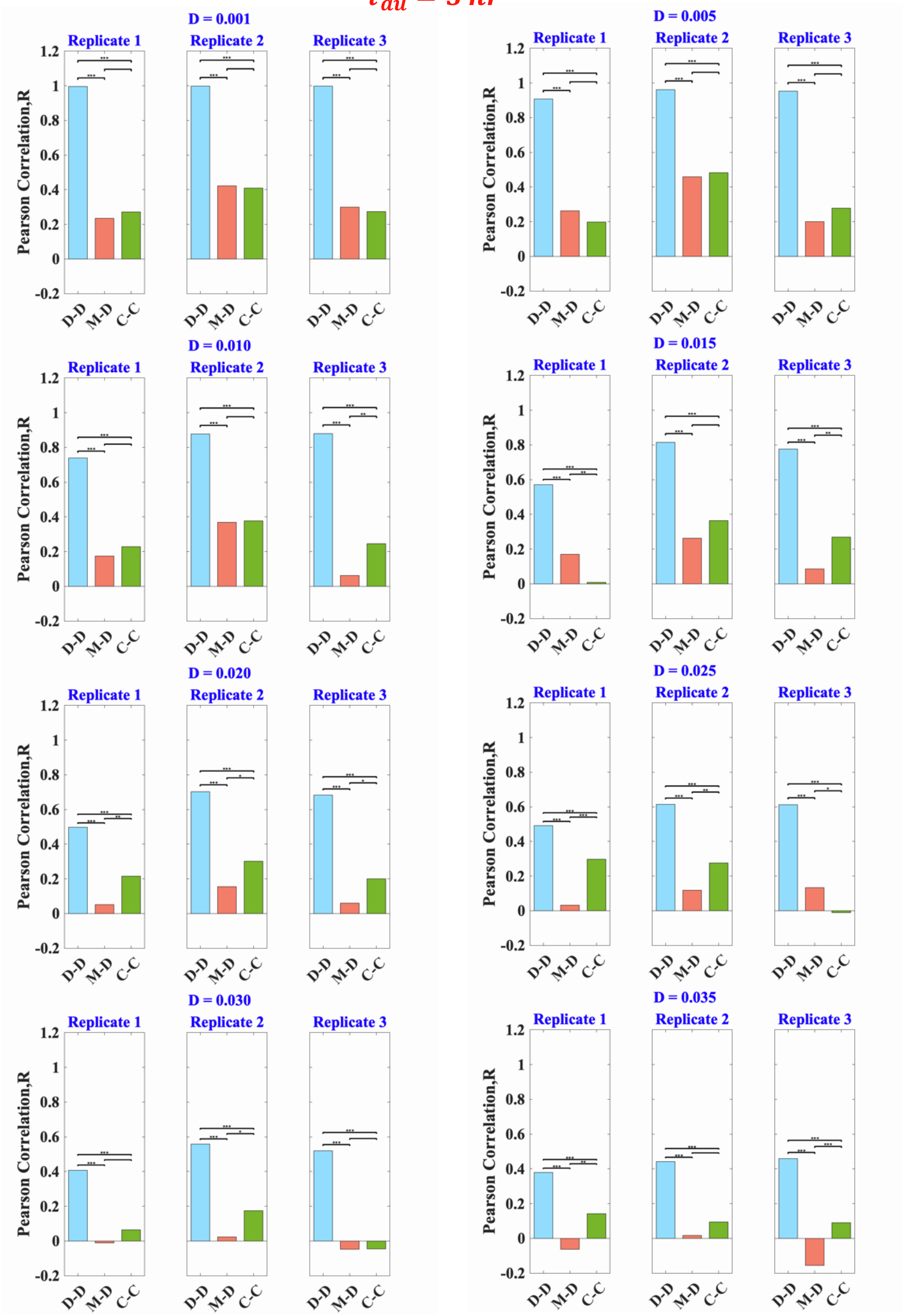

Figure S40 Significance analysis (Related to **Figure 3A(iv) & 3B(iv)**) of correlations between lineage pairs of individual replicates for τ_{au} value 5hr. (***p<0.05**, ****p<0.001, ***p<0.0001**). p indicates the p-value calculated using a Fisher's z transformation of correlation values from lineage pairs. The total cell cycle time is **28 hours** here.

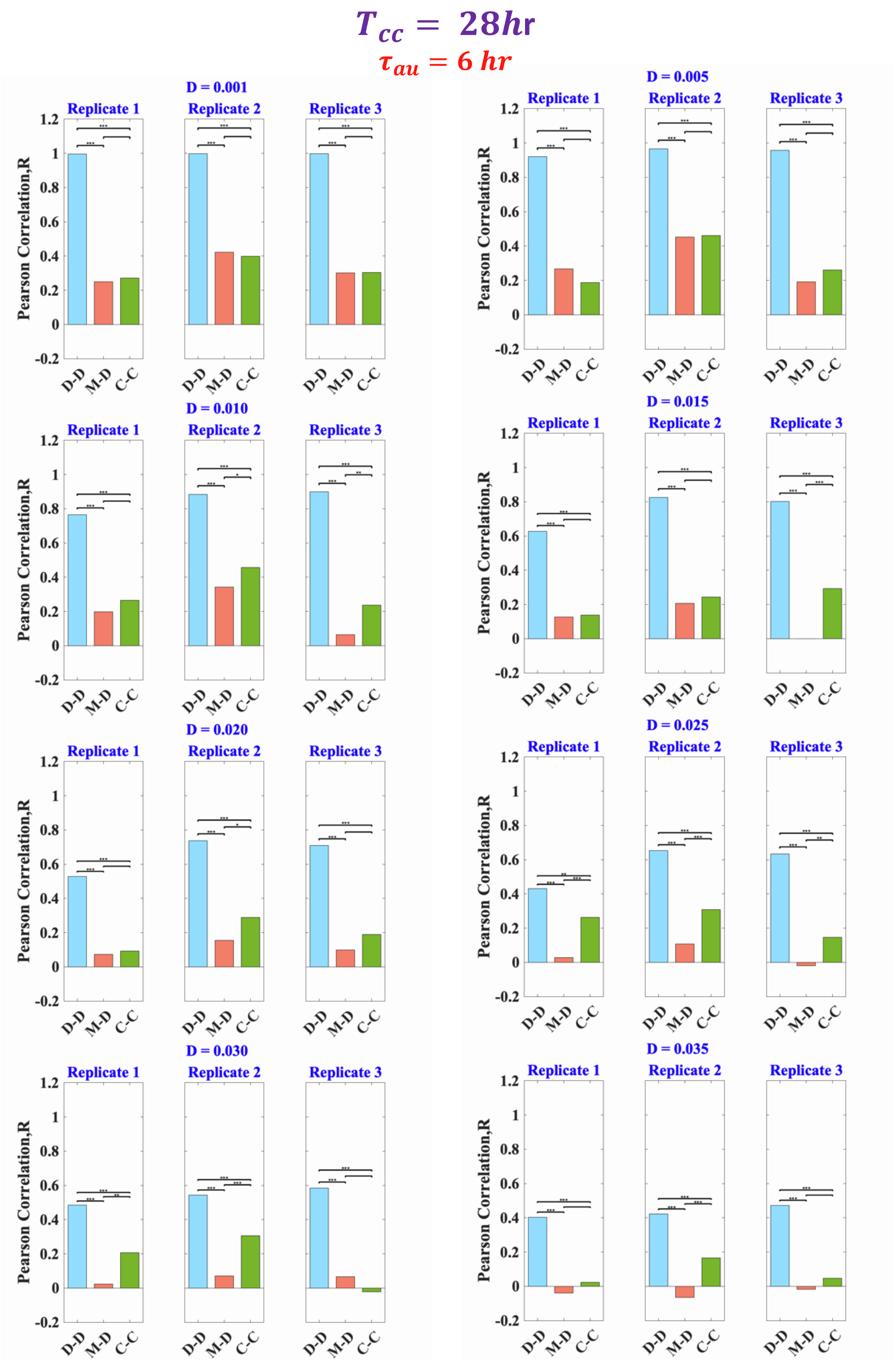

Figure S41 Significance analysis (Related to **Figure 3A(iv) & 3B(iv)**) of correlations between lineage pairs of individual replicates for τ_{au} value 6hr. (***p<0.05**, ****p<0.001, *****p<0.0001**). p indicates the p-value calculated using a Fisher's z transformation of correlation values from lineage pairs. The total cell cycle time is **28 hours** here.

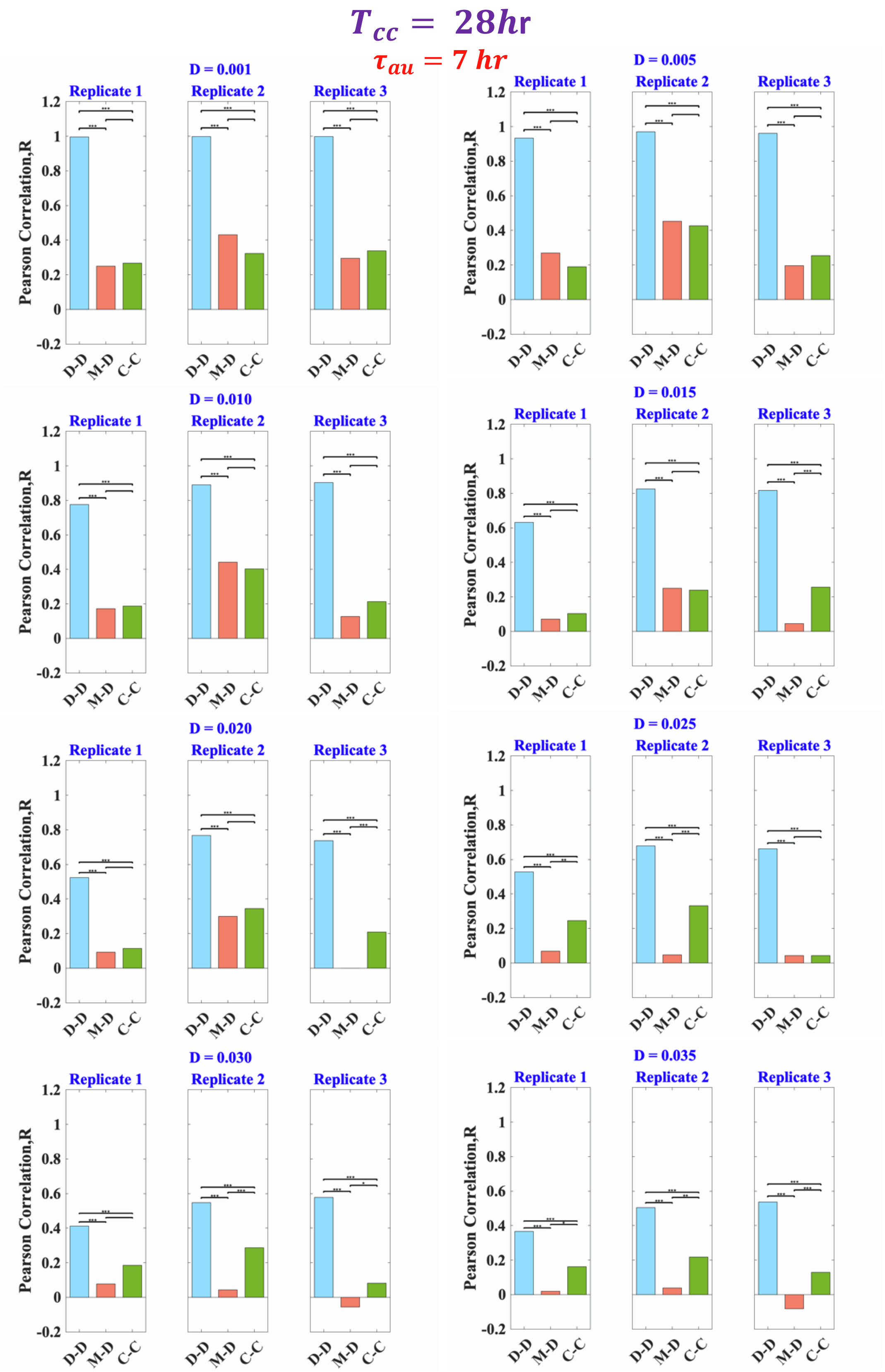

Figure S42 Significance analysis (Related to **Figure 3A(iv) & 3B(iv)**) of correlations between lineage pairs of individual replicates for τ_{au} value 7hr. (***p<0.05**, ****p<0.001, ***p<0.0001**). p indicates the p-value calculated using a Fisher's z transformation of correlation values from lineage pairs. The total cell cycle time is **28 hours** here.

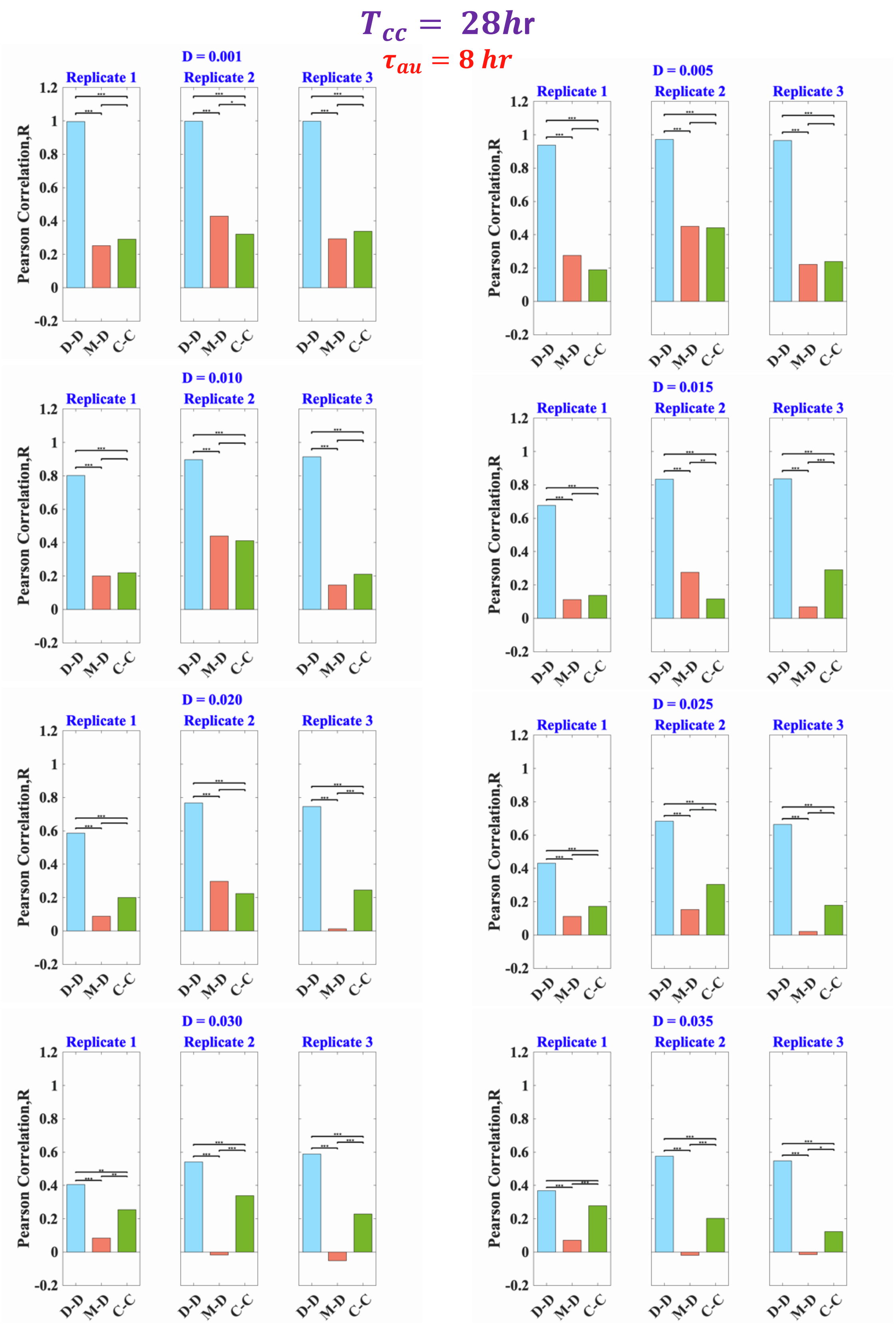

Figure S43 Significance analysis (Related to **Figure 3A(iv) & 3B(iv)**) of correlations between lineage pairs of individual replicates for τ_{au} value 8hr. (***p<0.05**, ****p<0.001, ***p<0.0001**). p indicates the p-value calculated using a Fisher's z transformation of correlation values from lineage pairs. The total cell cycle time is **28 hours** here.

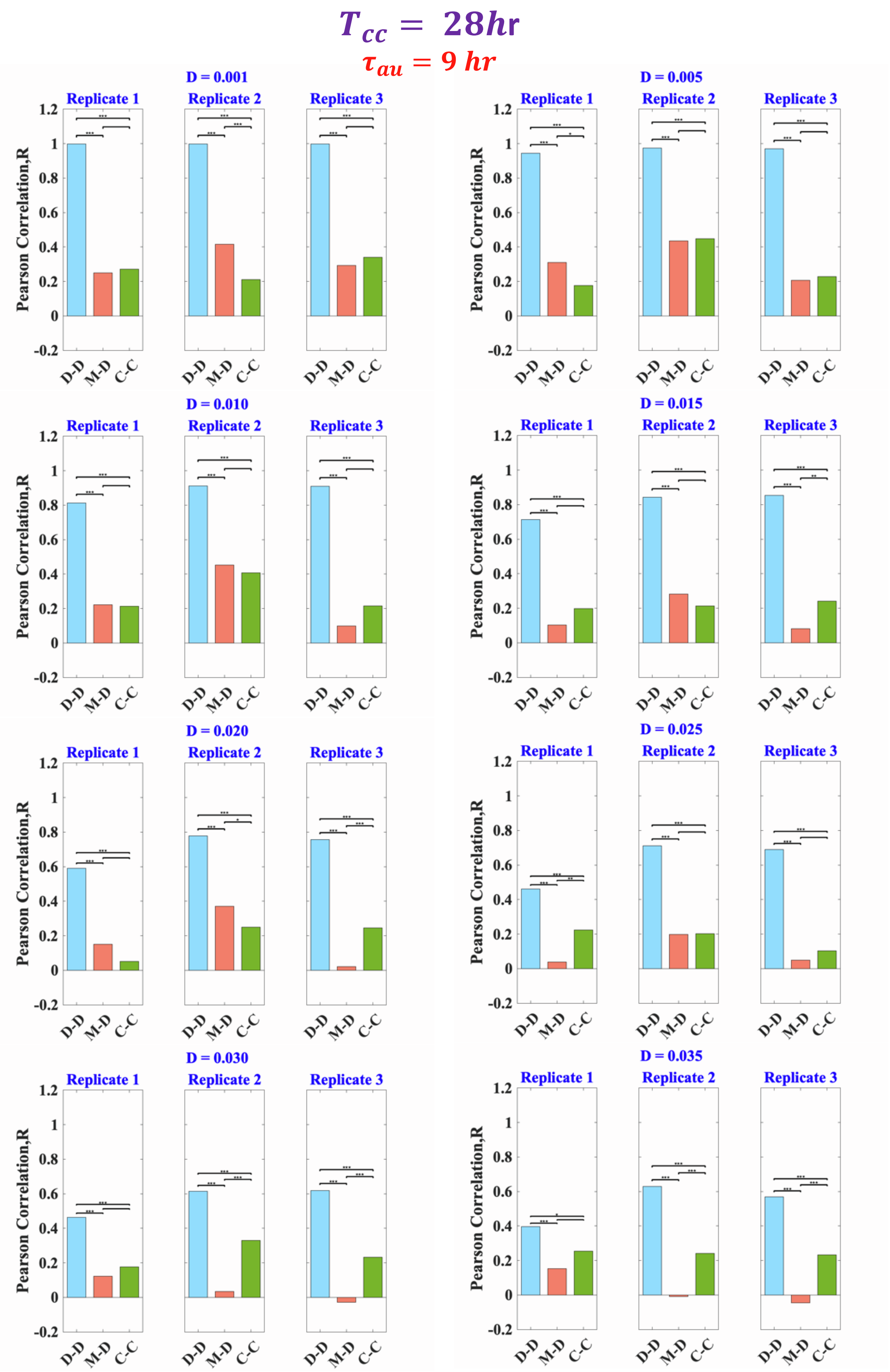

Figure S44 Significance analysis (Related to **Figure 3A(iv) & 3B(iv)**) of correlations between lineage pairs of individual replicates for τ_{au} value 9hr. (***p<0.05**, ****p<0.001, ***p<0.0001**). p indicates the p-value calculated using a Fisher's z transformation of correlation values from lineage pairs. The total cell cycle time is **28 hours** here.

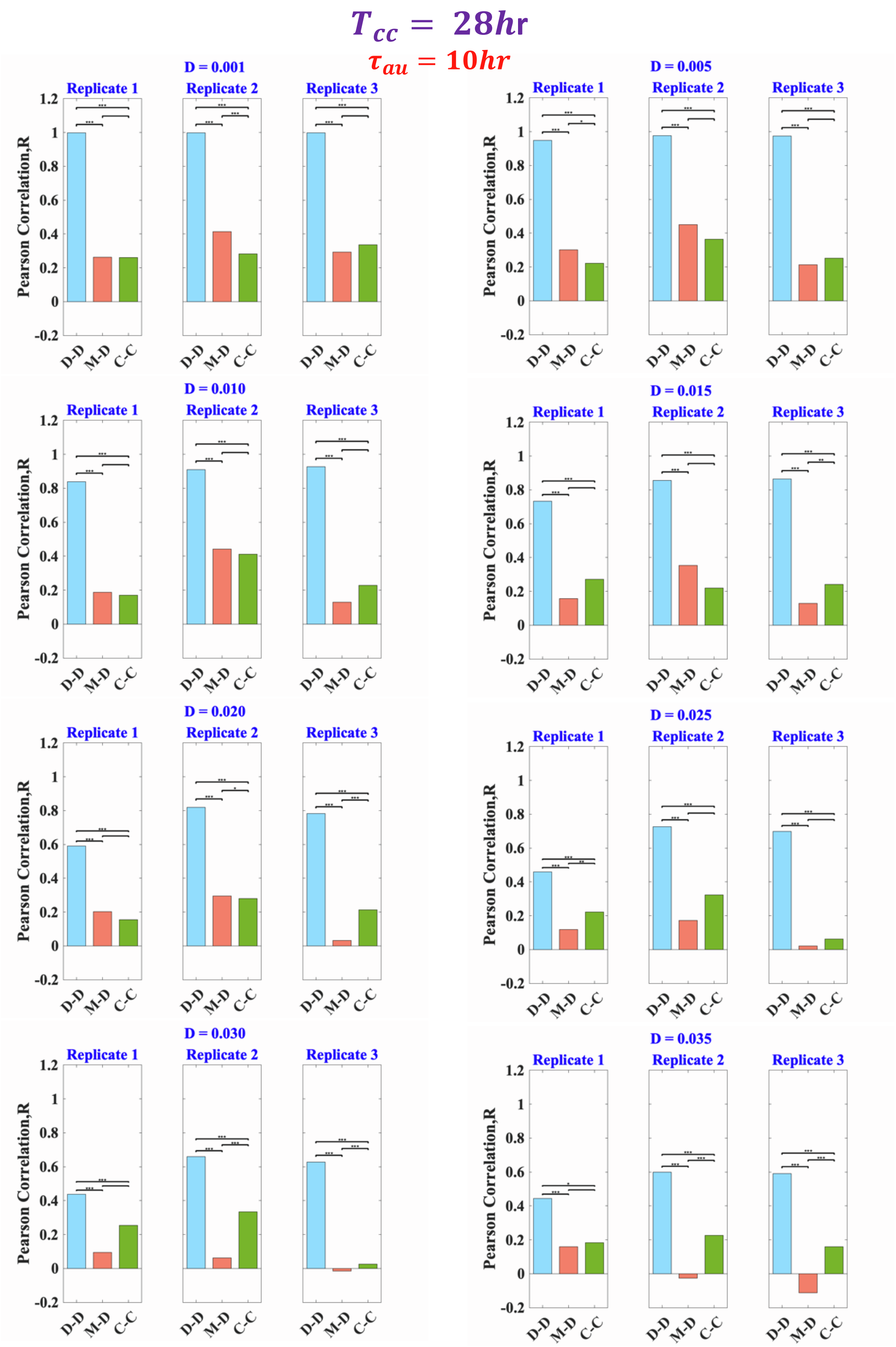

Figure S45 Significance analysis (Related to **Figure 3A(iv) & 3B(iv)**) of correlations between lineage pairs of individual replicates for τ_{au} value 10hr. (**$*p<0.05$, $**p<0.001$, $***p<0.0001$**). p indicates the p-value calculated using a Fisher's z transformation of correlation values from lineage pairs. The total cell cycle time is **28 hours** here.

$T_{cc} = 28hr$

$\tau_{au} = 15\ hr$

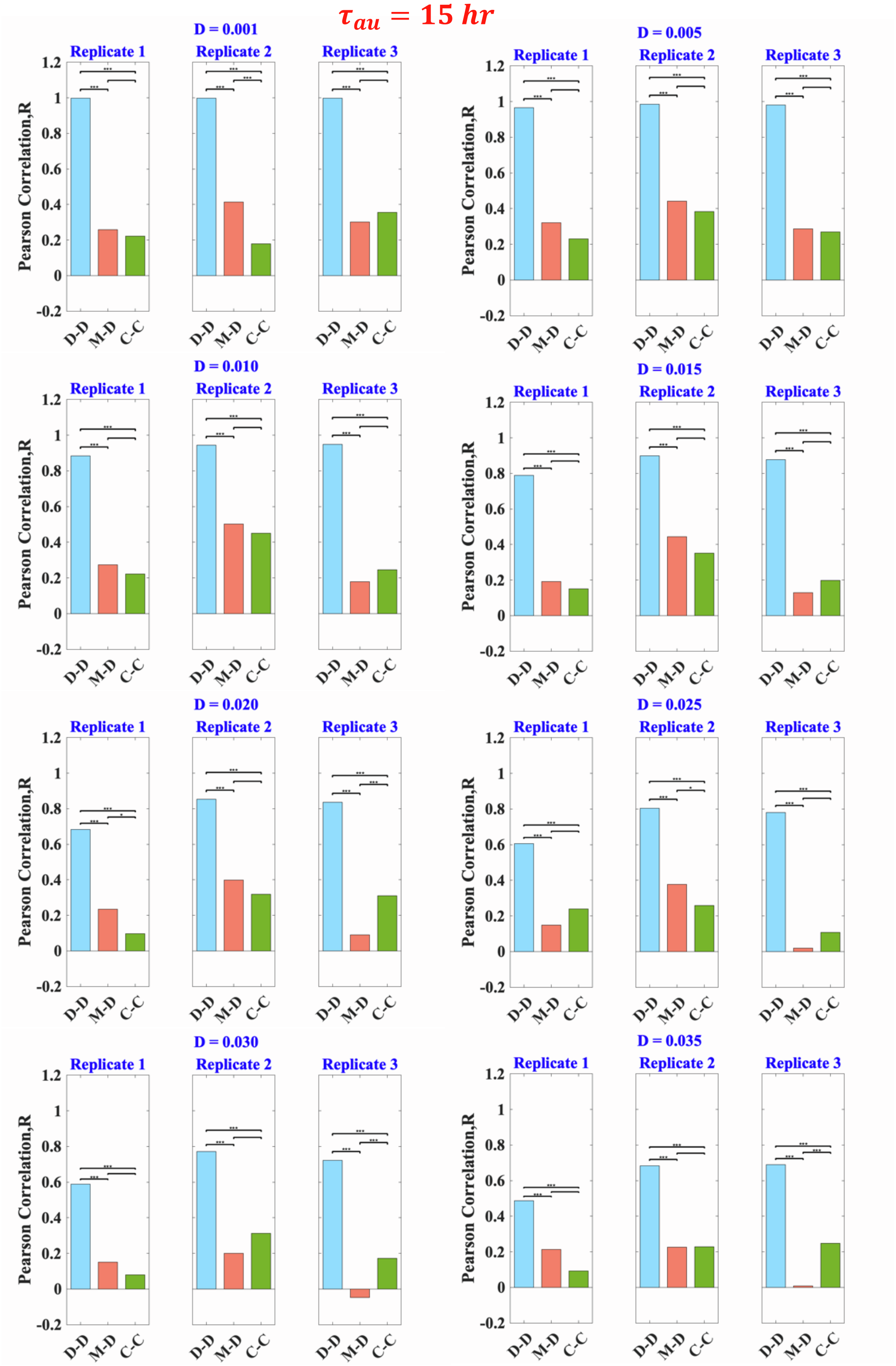

Figure S46 Significance analysis (Related to **Figure 3A(iv) & 3B(iv)**) of correlations between lineage pairs of individual replicates for τ_{au} value 15hr. (***p<0.05**, ****p<0.001, *****p<0.0001**). p indicates the p-value calculated using a Fisher's z transformation of correlation values from lineage pairs. The total cell cycle time is **28 hours** here.

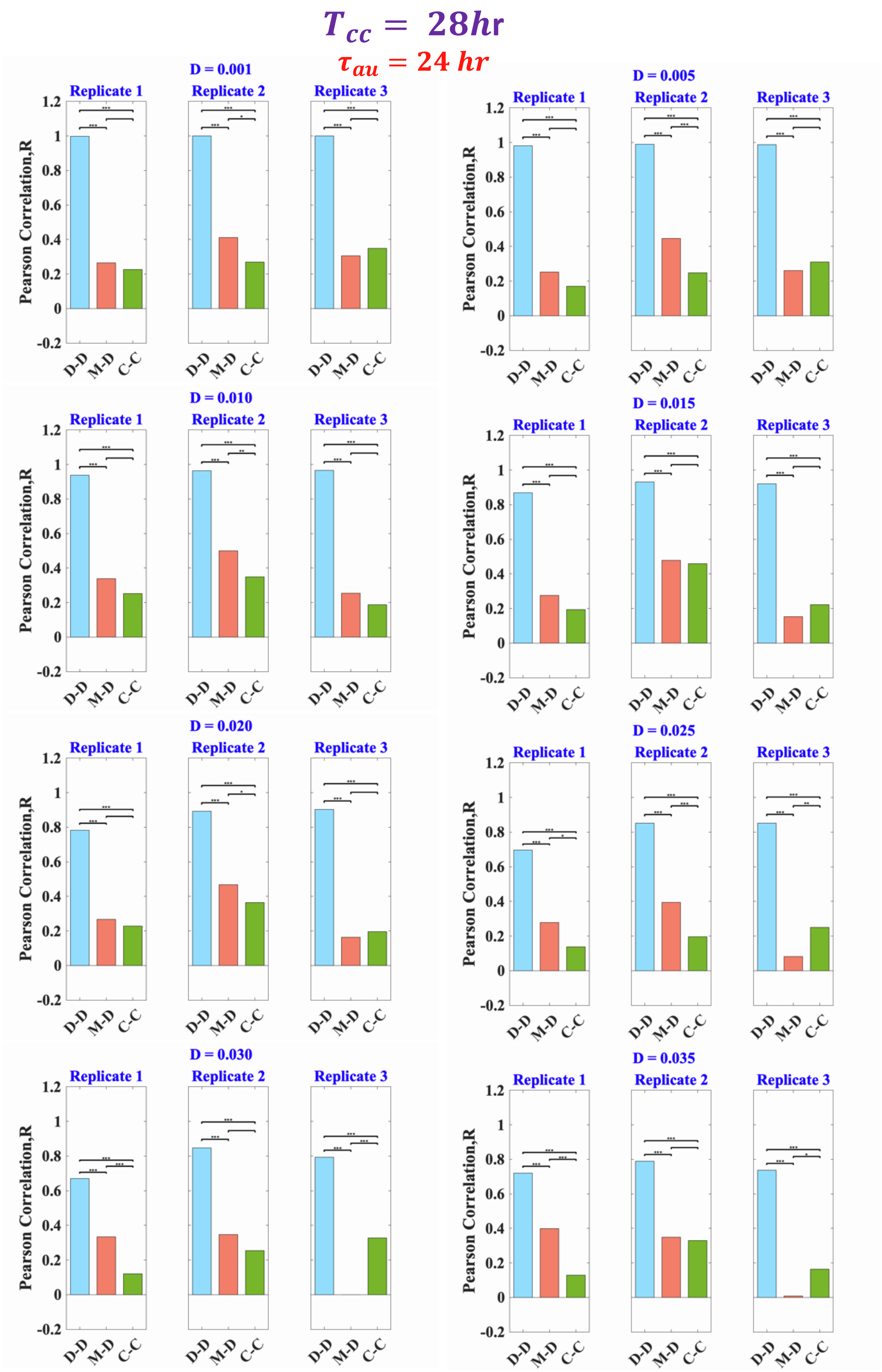

Figure S47 Significance analysis (Related to **Figure 3A(iv) & 3B(iv)**) of correlations between lineage pairs of individual replicates for τ_{au} value 24hr. (***p<0.05**, ****p<0.001, ***p<0.0001**). p indicates the p-value calculated using a Fisher's z transformation of correlation values from lineage pairs. The total cell cycle time is **28 hours** here.

S2 Supplementary tables (Table S1-S7)

Table S1 – Abbreviated form and description of variables governing cell cycle regulatory network in Figure S1A

Abbreviated form	Description	Initial condition (in Concentration unit)
$CycB_m$	CycB mRNA	0.05
$CycB$	CycB protein	0.3
$Cdc20_m$	Cdc20 mRNA	0.07
$CDC20T$	Cdc20 protein total	0.59
Cdh_m	Cdh1 mRNA	0.99
$Cdht$	Cdh1 protein total	0.99
$Cdh1$	The active form of Cdh1	0.006
$Cdc20A$	Active form of Cdc20	0.036
IEP	Intermediary enzyme	0.41

Table S2 - Ordinary Differential Equations governing the cell cycle regulatory network in Figure S1A.

$\frac{dCycBm}{dt} = \frac{(k_{1m} + \varepsilon_{ou1}) \times GF}{K_{mm} + k_{eff} * GF} - k_{1dm} \times CycBm$	**Equation 2**
$\frac{dCycB}{dt} = k_1 \times CycBm - k_{2a} \times CycB - k_{2b} \times CycB \times Cdh1$	**Equation 3**
$\frac{dCdc20m}{dt} = (k_{5am} + \varepsilon_{ou2}) + \frac{(k_{5bm} + \varepsilon_{ou3})}{k_{5cm} + GF \ \times J_{5c}} \times \frac{CycB^n}{J_5^n + CycB^n} - k_{5dm} \times Cdc20m$	**Equation 4**
$\frac{dCdc20T}{dt} = k_{5a} \times Cdc20m - k_6 \times Cdc20T$	**Equation 5**
$\frac{dCdc20A}{dt} = k_7 \times IEP \times \frac{Cdc20T - Cdc20A}{J_7 + Cdc20T \ - \ Cdc20A} - k_8 \times Mad \times \frac{Cdc20A}{J_8 + Cdc20A} - k_6 \times Cdc20A$	**Equation 6**
$\frac{dCdhm}{dt} = (k_{3m} + \varepsilon_{ou4}) - k_{3dm} \times Cdhm$	**Equation 7**

$$\frac{dCdht}{dt} = k_{3a} \times Cdhm - k_{3dt} \times Cdht$$	**Equation 8**
$$\frac{dCdh1}{dt} = (k_3 + k_{3b} \times Cdc20A) \times \frac{Cdht - Cdh1}{J_3 + Cdht - Cdh1} - k_4 \times CycB \times \frac{Cdh1}{J_4 + Cdh1} - k_{3dt} \times Cdh1$$	**Equation 9**
$$\frac{dIEP}{dt} = k_9 \times CycB \times (1 - IEP) - k_{10} \times IEP$$	**Equation 10**

Table S3 - Parameter values used for simulations governing cell cycle regulatory network in Figure S1A.

	**Full Description**	**Parameter value**	**Unit (c=concentration t=hour)**
k_{1m}	Transcription rate of CycB mRNA	$0.0037 \times d$	c t^{-1}
k_{1dm}	Degradation rate of CycB mRNA	$0.058 \times d$	t^{-1}
k_1	Synthesis rate of CycB	$0.4 \times d$	t^{-1}
k_{2a}	Degradation rate of CycB	$0.04 \times d$	t^{-1}
k_{2b}	The Cdh1-dependent degradation rate of CycB	$2.0\times d$	$(c\ t)^{-1}$
k_{3a}	The synthesis rate of Cdh1 total	$1.0 \times d$	t^{-1}
k_{3b}	Cdc20-dependent activation rate of Cdh1	$4.3 \times d$	$(c\ t)^{-1}$
k_4	CycB-induced degradation rate of Cdh1 active form	$40.0 \times d$	t^{-1}
J_3	Michalis-Menten coefficient for Cdc20-dependent activation rate of Cdh1	0.04	c
J_4	Michalis-Menten coefficient for CycB-induced degradation rate of Cdh1 active form	0.04	c
k_{3m}	Transcription rate of Cdh1 mRNA	$0.5 \times d$	c t^{-1}
k_{3dm}	Degradation rate of Cdh1 mRNA	$0.5 \times d$	t^{-1}
k_{3dt}	Degradation rate of Cdh1 total	$1.0 \times d$	t^{-1}
k_{5am}	Basal synthesis rate of Cdc20 mRNA	$0.005 \times d$	c t^{-1}
k_{5bm}	CycB-dependent transcription rate of Cdc20 mRNA	$0.2 \times d$	c t^{-1}
k_{5dm}	Degradation rate of Cdc20 mRNA	$1.386 \times d$	t^{-1}
J_5	Hill coefficient for CycB-dependent transcription rate of Cdc20 mRNA	0.3	c
n	Hill constant for CycB-dependent transcription rate of Cdc20 mRNA	4.0	-
k_{5a}	Synthesis rate of Cdc20 total	$1.0\times d$	t^{-1}
k_6	Degradation rate of Cdc20 total	$0.05 \times d$	t^{-1}

k_7	Activation rate of Cdc20A	$1.4 \times d$	t^{-1}
k_8	Degradation rate of Cdc20A	$0.5 \times d$	c t^{-1}
J_7	Michaelis-Menten for activation rate of Cdc20A	0.001	c
J_8	Michaelis Menten coefficient for inactivation rate of Cdc20A	0.001	c
k_9	Synthesis rate of IEP	$0.1 \times d$	$(c\ t)^{-1}$
k_{10}	Degradation rate of IEP	$0.02 \times d$	t^{-1}
k_{11}	Synthesis rate of X	$0.045 \times d$	c t^{-1}
k_{12}	CycB-mediated degradation rate of X	$2.27 \times d$	$(c\ t)^{-1}$
k_{13}	Degradation rate of X	$0.004 \times d$	t^{-1}
k_3	Basal synthesis rate of Cdh1 A	$1.28 \times d$	c t^{-1}
GF	Growth factor	2.0	
k_{mm}	Michalis-Menten constant for Growth factor-induced transcription of CycB mRNA	0.2	Dimensionless
k_{5cm}	Michalis-Menten constant for Growth Factor-inhibited transcription of Cdc20 mRNA	1.0	Dimensionless
J_{5c}	Effective GF concentration for inhibition of Cdc20 mRNA	0.02	Dimensionless
d	Scaling factor for cell cycle period adjustment	2.8	Dimensionless

Changes in the parameter values (from Table S3)

For **Figure 3**, d value changed as: T_{cc} = 16hr (d=4.35) , T_{cc} = 20hr (d=3.5), T_{cc} = 24hr (d=2.8), T_{cc} = 28hr (d=2.52)

For **Figure 4(A),** T_{cc} =16hr (T_{G1}=9hr+$T_{S\text{-}G2\text{-}M}$=7hr) – $k_4 = 15\ (\mathrm{t}^{-1}), k_1 = 1.1\ (\mathrm{t}^{-1})$,

Figure 4 (B), T_{cc} =16hr (T_{G1}=3hr+ $T_{S\text{-}G2\text{-}M}$ =13hr) $k_4 = 45\ (\mathrm{t}^{-1}),\ k_1 = 0.45\ (\mathrm{t}^{-1})\ k_{2b} = 1.8$,

Figure 4 (C), T_{cc} =28hr (T_{G1}=16hr+ $T_{S\text{-}G2\text{-}M}$ =12hr) – $k_4 = 20\ (\mathrm{t}^{-1}),\ k_1 = 0.6\ (\mathrm{t}^{-1})$

Figure 4 (D), T_{cc} = 28 hr (T_{G1}=9hr+ $T_{S\text{-}G2\text{-}M}$ =19hr) - $k_4 = 45\ (\mathrm{t}^{-1}),\ k_1 = 0.43\ (\mathrm{t}^{-1}), k_{2b} = 1.8\ (\mathrm{c\ t})^{-1}$ d = 1.8

Only the parameters specified here are changed; other parameters are kept the same as in **Table S3**.

Table S4 – Mixing time for different proteins and their functions (Mixing time (τ_m) was defined as the time when autocorrelation function A(t) decayed to one half) from Alex Sigal et al., 2005[1]

**Protein**	**Function**	τ_m $(generations)$
HMGA2	Transcriptional regulation	2.4 ± 0.5
HMGA1	Transcriptional regulation	2.2 ± 0.4
SET	Chromatin remodeling	2.2 ± 0.5
RBBP7	Cell proliferation and differentiation	1.5 ± 0.2
H2AFV	Regulatory histone	1.6 ± 0.4
LMNA	Nuclear membrane structure	1.6 ± 0.3
ANP32B	Pro-apoptotic	2.7 ± 0.4
GTF2F2	Recruitment of RNA polymerase II to promoters	1.2 ± 0.1
USP7	Deubiquitination	0.9 ± 0.2
SFRS10	Alternative splicing regulator	1.0 ± 0.2

Table S5 – Abbreviated form and description of variables for the network in Figure S7A
(Related to Figure S7A)

**Abbreviated form**	**Description**	**Initial condition (in Concentration unit)**
$CycB$	CycB protein	0.52
$CDC20T$	Cdc20 protein total	1.28
$Cdh1$	The active form of Cdh1	0.011
$Cdc20A$	Active form of Cdc20	0.32
IEP	Intermediary enzyme	0.58

Table S6 - Ordinary Differential Equations governing the cell cycle regulatory network in Figure S7A *(Related to Figure S7A)*

$\frac{dCycB}{dt} = \frac{(k_1 + \varepsilon_{ou1}) \times GF}{K_{mm} + k_{eff} * GF} - k_{2a} \times CycB - k_{2b} \times CycB \times Cdh1$	**SEq.1**
$\frac{dCdc20T}{dt} = (k_{5a} + \varepsilon_{ou2}) + \frac{(k_{5b} + \varepsilon_{ou3})}{k_{5cm} + GF \times J_{5c}} \times \frac{CycB^n}{J_5^n + CycB^n} - k_6 \times Cdc20T$	**SEq.2**
$\frac{dCdc20A}{dt} = k_7 \times IEP \times \frac{Cdc20T - Cdc20A}{J_7 + Cdc20T - Cdc20A} - k_8 \times Mad \times \frac{Cdc20A}{J_8 + Cdc20A} - k_6 \times Cdc20A$	**SEq.3**
$\frac{dCdh1}{dt} = ((k_{3a} + \varepsilon_{ou4}) + (k_{3b} + \varepsilon_{ou4}) \times Cdc20A) \times \frac{1 - Cdh1}{J_3 + 1 - Cdh1} - k_4 \times CycB \times \frac{Cdh1}{J_4 + Cdh1} - k_{3dt} \times Cdh1$	**SEq.4**
$\frac{dIEP}{dt} = k_9 \times CycB \times (1 - IEP) - k_{10} \times IEP$	**SEq.5**

Table S7 - Parameter values governing the cell cycle regulatory network in Figure S7A.
(Related to Figure S7A)

**Abbreviated Form**	**Full Description**	**Parameter value**	**Unit (c=concentration t=hour)**
k_1	Synthesis rate of CycB	$0.04 \times d$	$(c\ t)^{-1}$
k_{2a}	Degradation rate of CycB	$0.04 \times d$	t^{-1}
k_{2b}	The Cdh1-dependent degradation rate of CycB	$1.6 \times d$	$(c\ t)^{-1}$
k_{3a}	The synthesis rate of Cdh1	$1.0 \times d$	$(c\ t)^{-1}$
k_{3b}	Cdc20-dependent activation rate of Cdh1	$14.0 \times d$	t^{-1}
k_4	CycB-induced degradation rate of Cdh1 active form	$32.7 \times d$	t^{-1}
J_3	Michalis-Menten coefficient for Cdc20-dependent activation rate of Cdh1	0.04	c
J_4	Michalis-Menten coefficient for CycB-induced degradation rate of Cdh1 active form	0.04	c
J_5	Hill coefficient for CycB-dependent transcription rate of Cdc20	0.3	c
n	Hill constant for CycB-dependent translational rate of Cdc20	4.0	-
k_{5a}	Synthesis rate of Cdc20 total	$0.005\times d$	$(c\ t)^{-1}$
k_{5b}	CycB-dependent synthesis rate of Cdc20 total	$0.2\times d$	$(c\ t)^{-1}$
k_6	Degradation rate of Cdc20 total	$0.1 \times d$	t^{-1}
k_7	Activation rate of Cdc20A	$1.0 \times d$	t^{-1}
k_8	Degradation rate of Cdc20A	$0.5\times d$	$c\ t^{-1}$
J_7	Michaelis-Menten for activation rate of Cdc20A	0.001	c

J_8	Michaelis Menten coefficient for inactivation rate of Cdc20A	0.001	c
k_9	Synthesis rate of IEP	0.1× d	$(c\ t)^{-1}$
k_{10}	Degradation rate of IEP	0.02 × d	t^{-1}
k_3	Basal synthesis rate of Cdh1 A	1.28× d	$c\ t^{-1}$
GF	Growth Factor	2.0	Dimensionless
k_{mm}	Michalis-Menten constant for Growth factor-induced translation of CycB	0.2	Dimensionless
k_{5cm}	Michalis-Menten constant for Growth factor inhibited translation of Cdc20	1.0	Dimensionless
J_{5c}	Effective GF concentration for inhibition of Cdc20	0.02	Dimensionless
d	Scaling factor for cell cycle period adjustment	2.4	Dimensionless

S3 Supplementary references

(1) Sigal, A.; Milo, R.; Cohen, A.; Geva-Zatorsky, N.; Klein, Y.; Liron, Y.; Rosenfeld, N.; Danon, T.; Perzov, N.; Alon, U. Variability and Memory of Protein Levels in Human Cells. *Nature* **2006**, *444* (7119), 643–646. https://doi.org/10.1038/nature05316.

(2) Tyson, J. J.; Novak, B. Regulation of the Eukaryotic Cell Cycle: Molecular Antagonism, Hysteresis, and Irreversible Transitions. *J. Theor. Biol.* **2001**, *210* (2), 249–263. https://doi.org/10.1006/jtbi.2001.2293.
